# Human Transglutaminases: Updated Insights into Activation Mechanisms, Allosteric Regulation and Disease

**DOI:** 10.3390/ijms27072976

**Published:** 2026-03-25

**Authors:** Pablo Moya-Garrido, Laura P. Cano-Gómez, Beatriz Ibarra-Molero, Raquel Godoy-Ruiz, Encarnación Medina-Carmona

**Affiliations:** 1 Departamento de Química Física, Facultad de Ciencias, Unidad de Excelencia de Química Aplicada a Biomedicina y Medioambiente (UEQ), Universidad de Granada, 18071 Granada, Spainbeatriz@ugr.es (B.I.-M.); 2 Department of Biochemistry, Molecular Biology and Immunology, School of Medicine, University of Malaga, 29010 Malaga, Spain; 3 Research Network on Chronicity, Primary Care and Prevention and Health Promotion (RICAPPS), Research and Innovation Unit, Costa del Sol University Hospital, 29603 Marbella, Spain; 4 School of Health Sciences and Sports, Alfonso X University Mare Nostrum, 29004 Malaga, Spain

**Keywords:** human transglutaminases, allosteric regulation, conformational dynamics, calcium binding, protein crosslinking, autoimmune diseases, genetic disorders, rare diseases, structure–function relationship

## Abstract

Human transglutaminases (hTGs) are Ca^2+^-dependent enzymes that catalyze protein crosslinking, deamidation and other post-translational modifications, thus acting as key stabilizers of tissue architecture and modulators of protein function across diverse physiological contexts. This family comprises eight catalytically active members, TG1-7, the blood coagulation factor FXIII, and the inactive structural protein Band 4.2 of the erythrocyte membrane. Recent structural and biochemical advances have refined our understanding of the molecular principles governing transglutaminase function. Thus, current evidence reveals how domain organization and catalytic architecture integrate calcium binding, nucleotide-dependent regulation in TG2 and proteolytic activation in selected isoforms to control enzymatic activity. In this review, we provide an updated and comprehensive overview of the active hTGs, combining structural, biochemical and functional data to explain how closely related enzymes achieve isoform-specific regulation and distinct biological roles. We further examine how disruption of these mechanisms contributes to human pathology, highlighting representative examples in autoimmunity, inherited disorders and complex diseases. By integrating recent biochemical and structural findings with disease-associated evidence, we aim to offer a coherent framework for understanding how TG regulation underlies their diverse biological functions and clinical relevance.

## 1. Human Transglutaminases: A Family Depiction

### 1.1. Introduction

**Figure 1:**
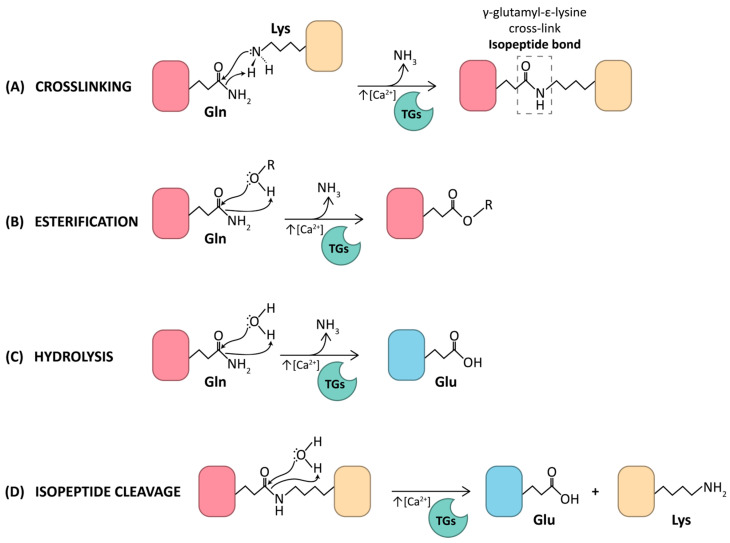
Transglutaminase-catalyzed reactions. (**A**) Transamidation (crosslinking): reaction forms isopeptide bonds between the side chains of reactive Gln residues and Lys residues. (**B**) Esterification: in the presence of alcohol-containing molecules, TGs catalyze the formation of ester bonds. (**C**) Deamidation: in the absence of reactive Lys residues or other primary amines, water acts as a nucleophile, resulting in the conversion of Gln residues to Glu residues. (**D**) Isopeptidolysis: TGs catalyze the cleavage of pre-existing isopeptide bonds, regenerating the original Glu and Lys side chains.

Transglutaminase proteins (EC 2.3.2.13), TGs, are a family of structurally and functionally related enzymes that catalyze permanent post-translational modifications of specific glutamine residues on proteins. These modifications refer, in most cases, to transamidation reactions where an isopeptide bond between the γ-carboxamide group of a protein-bound glutamine and the ε-amino group of a protein-bound lysine is formed. Alternatively, incorporation of non-protein monoamines and polyamines can also occur, generating protein–polyamine conjugates that can be further polymerized (Figure 1). As a result, these crosslinking and polyamidation activities can produce high-molecular-weight protein networks with altered properties regarding stability, solubility, proteolytic and mechanical resistance, and immunogenicity. This has led to the designation of TGs as Nature’s biological glues [1]. Physiological functions related to the activities mentioned include, for instance, formation of blood clots during wound healing [2,3,4,5,6], assembly of the protein cornified envelope, which is critical in the formation and maintenance of the skin [7,8,9], and polyamination of neural tubulins [10] to generate stable microtubules required for neuronal structures and functions. In addition, TGs can catalyze deamidation and esterification reactions of glutamine side chain in case water or alcohol molecules, respectively, replace suitable amine donor substrate (Figure 1). That is the case, for instance, of enzymatic deamidation of gliadin peptides, which has been linked to the immune response associated with gluten sensitivity [11,12,13,14], and the covalent attachment of long-chain ω-hydroxyceramides through esterification of structural cornified envelope proteins in the skin [9,15,16].

TGs are extended across all kingdoms of life [17]. In particular, the study of microbial TGs has attracted the interest of researchers due to the extensive use of their polymerizing properties in industrial applications. Thus, food, textile, pharmaceutical and biotechnology industries have benefited significantly from their utilization as tools not only to improve the quality of the target products but also in a sustainable way. For recent reviews on this topic, see [18,19,20,21,22,23] and chapters 8–13 in [17]. However, the focus of this review will be centered on active human transglutaminases (hTGs). The mammalian TG family comprises eight Ca^2+^-dependent catalytically active enzymes, TG1-7 (genes *TGM1-7*) and the blood coagulation Factor FXIIIA (gene *F13A1*), and the inactive structural protein Band 4.2 of erythrocyte membranes. In terms of tissue distribution, *in vivo* function and mechanisms of regulation, there are significant differences among all hTG family members, as will become evident throughout this review.

### 1.2. The TG Family

TG1 (also known as keratinocyte TG), TG3 (or epidermal TG), and TG5 are mainly expressed in stratified squamous epithelium, such as the epidermis of the skin and the esophageal epithelium. In the skin, they specifically facilitate keratinocyte differentiation and death, thus being crucial for skin and hair barrier assembly [8,16,24,25,26]. In fact, *TGM1* is an essential gene as shown by the unviability of born knockout mice [27]. And beyond the skin, low levels of TG1 expression have also been detected in the brain, lung, heart, kidney and liver. Its emerging role in a number of physio-pathological conditions, such as vasculature, hemostasis, neurodegenerative diseases, cancer, fibrosis, and so on, has been pointed out (see, for an insightful review: [8]).

The ubiquitous TG2 (or tissue TG), the most comprehensively studied member of the family together with FXIII, is actually an unusual and extraordinary multi-faced enzyme involved in a myriad of biological processes [28,29]. In addition to the classical Ca^2+^-dependent crosslinking activity, TG2 also displays GTPase, ATPase, protein kinase and protein disulfide isomerase activities [29]. Interestingly, the binding of GTP allosterically inhibits its transamidation activity [30] and instead enables it to act as a cytosolic G protein, mediating cell signaling transduction [31]. Hence, the bioavailability of Ca^2+^ and GTP, as well as cysteines’ redox state and enzyme location (intracellular or extracellular), appear to be the main determinants of TG2’s role by promoting appropriate competent conformations [32]. Moreover, a greater level of complexity in TG2 emerges due to non-enzymatic protein–protein interactions, identified in the mammalian proteome, that may play a key role in modulating and expanding its cellular functions in a context-specific manner [33,34]. This whole repertoire of different enzymatic and non-enzymatic activities, fine-tuned regulated [35,36], allows TG2 to participate in relevant physiological processes as well as pathological conditions, some of which have yet to be fully understood. Thus, examples include cell proliferation, apoptosis, cancer, neurodegenerative diseases, senescence, wound healing and celiac disease [37,38,39,40,41,42,43,44].

Like TG2, FXIIIA, or catalytic A subunit of Factor XIII, has been thoroughly studied and serves as another example of a multifunctional protein [2,6,45,46]. In fact, FXIII can exist as a plasma protein, consisting of a tetramer composed of two catalytic A subunits and two B subunits (A_2_B_2_), as well as an intracellular protein consisting of two catalytic A subunits. The B subunits play a role in the regulation, protection and stabilization of the enzyme complex and are mosaic proteins made up of 10 tandem repeats called sushi domains [47]. FXIIIA carries the transamidation activity and is able to crosslink fibrin strands during the blood clotting cascade, thus having a pivotal role in hemostasis [48]. In addition, other functions, including wound healing, maintenance of pregnancy, angiogenesis, apoptosis and bacterial entrapment as part of the defense mechanism against pathogens, have been reported [6]. FXIII can also exist in a cellular form, being expressed in platelets and other cell lines, including fibroblasts, chondrocytes, osteoblasts, adipocytes and macrophages. Previous studies have demonstrated the involvement of three transglutaminases, TG1, TG2 and FXIIIA, in bone resorption and osteoclastogenesis and that their inhibition leads to blockage of osteoclastogenesis [49,50]. Several recent reviews provide comprehensive discussions of FXIII implications in human disease [6,46,51,52].

Clearly, the less-studied members of the family are TG4, TG6 and TG7, with their *in vivo* functions remaining unknown. On one hand, TG4, also known as prostate TG, is primarily present in human seminal fluid and its expression is increased in aggressive prostate cancers and has been related to infertility. In fact, recent studies identify TG4 as a potential diagnosis and prognosis opportunity in prostatic diseases and, moreover, a target for immunotherapy [53,54,55]. Recently, its *in vitro* biochemical characterization has been published [56] and, interestingly, it exhibits the lowest transamidation activity among hTGs. It also lacks the GTP-binding site. TG6 (or neuronal TG) expression is associated with neurogenesis in the central nervous system and its role in neurons controlling motor function is strongly suggested by the connection of mutations found in the *TGM6* gene with spinocerebellar ataxia type 35 (SCA35) [57,58], as will be described in Section 3.2.4. In addition, previous studies relate TG6 to a number of neurological disorders such as Parkinson’s, progressive multiple sclerosis and amyotrophic lateral sclerosis [59,60,61,62,63,64,65]. According to The Human Atlas database (https://www.proteinatlas.org/), last accessed on 5 February 2026, the TG6 expression profile is not detected, and no RNA expression is available. Finally, TG7 is, by far, the greatest unknown member in the family. In fact, its expression and localization patterns are not available according to The Human Protein Atlas and tissue RNA expression clusters in testis, being enhanced in lymphoid tissue. As a first step for elucidating its physiological role, the identification of preferred TG7 substrate sequences has been carried out based on the screening of a phage-displayed peptide library [66]. More recently, TG7 has been proposed as a potential biomarker and therapeutic target in testicular cancer [67].

### 1.3. Evolutionary Relationship Among TG Members

**Figure 2:**
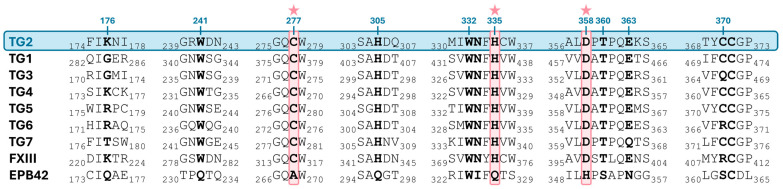
Sequence alignment of selected regions containing functional active-site residues in the hTG family, using TG2 as the query sequence (indicated by the blue box) Following TG2 numbering, the catalytic triad members (Cys277, His335, and Asp358) are highlighted with pink boxes and marked with stars above the alignment. Lys176 and Asn333 in TG2 are central components of an intricate network of primarily backbone-mediated noncovalent interactions that govern substrate specificity. Trp241 stabilizes the oxyanion of the tetrahedral intermediates through hydrogen bonding and, together with Trp332 and Thr360, defines the architecture of the active-site tunnel. Furthermore, His305 and Glu363 maintain a calcium-facilitated spatial orientation, which is pivotal for directing the reaction towards transamidation over deamidation. Finally, Cys370 and Cys371 serve as the key residues responsible for the redox regulation of enzymatic activity (see Section 1.4 for details).

The TG family is closely related to the papain-like cysteine protease family, which makes up part of the cysteine protease superfamily in the Structural Classification of Proteins (SCOP) database [68,69]. As expected, the papain-like catalytic triad Cys-His-Asp is well-conserved among all active members of the family (Figure 2). In contrast, the catalytic triad is actually lacking in the inactive Band 4.2, which has a scaffolding role in the erythrocyte membrane skeleton.

In terms of evolutionary relationships among mammalian TGs, phylogenetic analyses have revealed that an early gene duplication event gave rise to two main branches with slightly different gene organization: one comprises genes encoding TG2-7 and band 4.2, which contain 13 exons, and the second includes genes for TG1 and FXIIIA, with 15 exons. It is believed that exon 9 from the former group was split into exons 10 and 11 in the TG1 and FXIIIA genes, while their non-homologous N-terminal extensions include an additional non-coding exon [7,70]. In these genes, exon 2 encodes for an amino-terminal propeptide sequence that provides specialized properties in terms of membrane association (for TG1) and proteolytic activation (for FXIIIA), as will be detailed in Section 1.6.

An updated phylogenetic analysis of the evolutionary history of TGs in vertebrates has recently been reported [71], identifying TG1 as the phylogenetically oldest epithelial transglutaminase. Furthermore, specific changes in the TG gene family have been associated with relevant evolutionary adaptations of the cornified structures in vertebrate skin.

The overall amino acid sequence similarity among hTGs ranges from 40 to 60%. As expected for homologous proteins, the overall spatial organization is highly conserved and corresponds to four sequential domains: a N-terminal β-sandwich, an α/β-catalytic core that harbors the catalytic and Ca^2+^ binding sites, and two C-terminal β-barrels involved in the regulation of enzyme activity. As will be discussed in Section 2.1, atomic structures have been solved only for TG2 [72,73], TG3 [74] and FXIII [75].

### 1.4. Catalytic Mechanism

**Figure 3:**
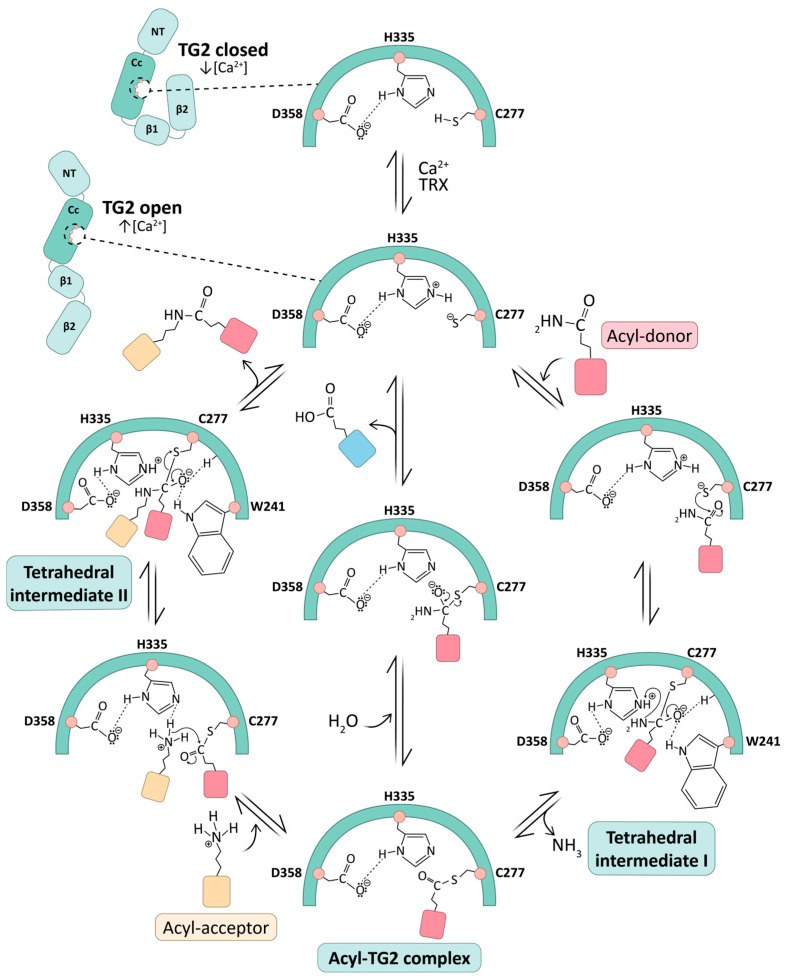
Mechanism of TG2 catalysis and intermediate stabilization. TG2 activation requires thioredoxin (TRX)-mediated reduction of the Cys370-Cys371 disulfide bond and Ca^2+^ binding to sites S1 and S3. Occupancy of these sites prevents TG2 oxidation and promotes formation of the γ-glutamyl thioester intermediate, respectively. Calcium binding triggers a large-scale conformational rearrangement, displacing the β-barrel domains (β1 and β2) away from the catalytic core (Cc) and the β-sandwich (NT), thereby exposing the active site and enabling substrate entry. The catalytically active imidazolium thiolate attacks the amide substrate carbonyl of a reactive Gln, forming the tetrahedral intermediate I. Substrate specificity is ensured by an intricate network of primarily backbone-mediated noncovalent interactions that correctly position the reactive side chain for nucleophilic attack by Cys277 [72]. The resulting acyl–enzyme complex can undergo aminolysis or hydrolysis, depending on the availability of an acyl-acceptor (e.g., Lys side chain) or water molecules in the catalytic channel. The tetrahedral intermediates I and II are stabilized by Trp241 via the formation of an H-bond with the cysteine–glutamine adduct, enabling the formation of the acyl–enzyme complex and the final isopeptide product, respectively. Colored boxes (orange, red and blue) represent the polypeptide backbones of the protein substrates.

A detailed description of the catalytic mechanism of the transamidation reaction of TG2 (Figure 3), for which comprehensive knowledge of its atomic structure is available, has been reported [72,76,77,78] and is assumed to extend to other homologues in the family [79,80], albeit with differences in enzyme regulation and substrate specificity. Recent structural findings by Sewa et al. [72] have shed light on novel details on the nature of regulation of TG2 activity by Ca^2+^ ions, as described later in this section.

In addition to the previously mentioned catalytic triad (Cys277/His335/Asp358 in TG2), there is a strict conservation of residues Trp241, Trp332 and Thr360 (numbering according to TG2), which form the active-site tunnel [81] among all active hTGs. Thus, in the presence of Ca^2+^, transamidation involves two consecutive steps of acylation and deacylation upon binding of a Gln-containing substrate (known as acyl-donor substrate) and an amine acyl-acceptor substrate, respectively, accommodating a modified ping-pong mechanism. Briefly, the thiol group of the active-site Cys (Cys277 in TG2/Cys315 in FXIII/Cys273 in TG3) forms a thiolate–imidazolium ion pair with a nearby His (His335 in TG2/His374 in FXIII/His331 in TG3), which is in turn hydrogen-bonded to an Asp (Asp358 in TG2/Asp397 in FXIII/Asp354 in TG3). This charge-relay system is, in fact, common to cysteine proteases and is essential for activating the catalytic Cys. Then, a nucleophilic attack by the active-site thiolate on the carboxamide group of a protein-bound Gln leads to the formation of a tetrahedral intermediate, which rearranges to produce the acyl–enzyme complex (TG covalently linked to the Gln-substrate via a γ-glutamyl thioester bond) and the simultaneous release of ammonia. This complex can be resolved in two ways to regenerate the free enzyme, either through a transamidation reaction or a hydrolysis reaction.

Recent discoveries by Prof. Khosla’s group have dissected the specific role of Ca^2+^ on the formation and resolution of the acyl–enzyme complex, as well as the relevance of non-active residues, which turn out to be essential for TG2 catalytic activity [72]. For the first time, the crystallographic structure of hTG2 bound to an inhibitory gluten peptidomimetic and two Ca^2+^ ions bound at sites S1, the high-affinity site, and S3, out of the five reported, is now available, providing an improved model for understanding the catalytic mechanism (Figure 3). This intermediate state, as described by the authors, completes the paradigmatic open–closed conformations of allosteric regulation in TGs, in which the open, Ca^2+^-induced conformation is catalytically active, whereas the closed one is inactive. In particular, S1 occupancy is proposed to prevent the Cys370/Cys371 disulfide bond formation, essential for TG2 activity. On the other hand, these structural studies provide experimental evidence of the fundamental role of metal binding to the low-affinity S3 site for γ-glutamyl thioester formation and for promoting the correct orientation of the dyad His305/Glu363, this step being key in resolving this thioester intermediate into an isopeptide bond (transamidation) but not in hydrolysis (deamidation). In fact, Ca^2+^ binding to S3 induces a dramatic reorganization, bringing His305 and Glu363 into close proximity and promoting the formation of a tunnel leading toward the catalytic triad. This rearrangement is predicted to accommodate the amine substrate appropriately during the second half of the transamidation reaction. In addition, the structural analysis highlights fine-tuned regulation of substrate recognition, as exemplified by key noncovalent interactions involving Asn333 and Lys176.

Regarding the catalytic mechanism, once the thioester intermediate is formed, it can react with an acyl-acceptor amine of a second substrate, corresponding to either a protein-bound Lys or a small molecule amine, which binds to the active site tunnel from the opposing direction to that of the first substrate. Specifically, the amine substrate, an alkyl ammonium cation at physiological pH, undergoes a double deprotonation facilitated by the same charge-relay system involved in the acylation step (His335/Asp358) and an additional interacting pair, His305/Glu363. This was actually proposed back in 2013, based on comparative studies with the structure of Ca^2+^-activated FXIII without the benefit of structural information [82], and is now experimentally supported by Khosla and coworkers [72], as mentioned above. Next, a nucleophilic attack on the thioester carbonyl results in the formation of a second tetrahedral intermediate, which has been shown to be rate-limiting in the deacylation step. Finally, its breakdown will generate the crosslinked product and restore the catalytic Cys of the enzyme to its original form. The indole group of Trp241 has been revealed to be essential in stabilizing the oxyanion of both tetrahedral intermediates in the acylation and deacylation steps. Interestingly, this residue is absent in the inactive member of the family Band 4.2 (see Figure 2). Alternatively to transamidation, if water gains access to the active site, the acyl–enzyme complex can undergo hydrolysis to form glutamic acid, resulting in a net deamidation (Figure 3).

These structural and biochemical studies are of great significance, as they have enabled a more detailed and mechanistic understanding of TG2 catalysis. These insights are expected to have a substantial impact on the rational design of next-generation TG2 inhibitors with improved specificity and efficacy.

### 1.5. TG Substrate Proteins

Each member of the TG family exhibits a distinct substrate profile, which has yet to be fully characterized in most cases. Elucidating their substrate specificity in physiological and pathological scenarios is essential to clarify their functional roles, the metabolic pathways in which they are involved, and their spatial and temporal localization *in vivo* across different tissues. Such knowledge is essential not only for advancing our understanding of TG biology but also for its significant translational implications [83], particularly in guiding the development of future therapeutic strategies, as will become clear in this section.

Since the 80s [84,85], considerable effort has been devoted to deciphering the molecular determinants that govern the binding of natural substrate proteins to TGs and, in particular, the factors that confer enhanced reactivity to certain Gln residues in the acyl-donor substrate and to certain Lys residues in the acyl-acceptor substrate in the crosslinking reaction. Most studies have primarily focused on Gln specificity, whose side chain is attacked by the catalytic Cys to produce the acyl–enzyme complex in the transamidation reaction. It is now known that no strict consensus sequence defines a specific target for each TG. Instead, substrate recognition and reactivity are influenced by the presence of preferred amino acids at defined positions around the reactive Gln, as well as by conformational features that facilitate formation of the first tetrahedral intermediate, the rate-limiting step of the reaction (see Figure 3).

Identification of endogenous substrate proteins presents additional challenges arising from *in vitro* cross-reactivity and the colocalization of multiple isozymes in certain tissues, as is the case for TG1, TG3, TG5 and TG6 in the skin [26] or TG1, TG2 and FXIIIA in osteoclasts [49]. At this point, it is pertinent to highlight that since the TRANSDAB (Transglutaminase Substrate Database) wiki (http://genomics.dote.hu/wiki/index.php/Main_Page) ceased to be updated in 2010, there are currently no actively maintained, TG-focused databases available that comprehensively compile substrates and interaction partners of hTGs.

Functional proteomics has been used to identify TG substrates and, when tandem mass spectrometry (MS) coverage allows, map reactive residues by combining activity-based probes (typically exogenous amine or acyl-donor mimics such as 5-(biotinamido)pentylamine, azide/alkyne-tagged “clickable” amines, dansyl-cadaverine, or other fluorescent probes) followed by affinity enrichment and Liquid Chromatography (LC)-tandem MS (LC-MS/MS) analysis [56,86,87,88,89]. It has also been successfully applied in tissue and disease models for the in situ identification of endogenous TG substrates, including pulmonary tissue [90], and to MS-based mapping of TG2-gluten isopeptide crosslinks [91]. The functional relevance of FXIII-mediated substrate incorporation into clots has been further highlighted in trauma-associated fibrinolytic phenotypes [92].

Pioneered studies from Prof. Hitomi’s laboratory using phage display and cDNA display have been seminal in defining the preferred substrate sequences of different TGs. Both techniques allow *in vitro* screening of large peptide libraries, in which genotype and phenotype are stably linked. In the phage display studies, random 12-mer peptides are presented on the surface of M13 phages, each serving as a Gln-donor substrate while biotin-labeled cadaverine acts as the acyl acceptor. When active TG is present, peptides containing suitably positioned reactive Gln residues become biotinylated and are selectively enriched by affinity purification. After five to six rounds of screening and enzymatic selection, the enriched phage pool is analyzed by sequencing the DNA encoding the displayed peptides, enabling the identification of highly TG-reactive peptide motifs. The so-called Hitomi peptides include K5 (TG1), T26 (TG2), E51 (TG3), F11 (FXIIIA), Y25 (TG6) and Z3 (TG7) [66,93,94,95,96,97], where the isozyme specificity is indicated in parentheses. These peptides, labeled with either a fluorophore or biotin, have been widely used for:

(1) *In vitro* and *in situ* detection of TG-specific activity, in cell extracts or tissue sections, even in the presence of different isozymes. This approach has been successfully applied for in situ detection of active TG1, TG2 and TG3 in mouse whole-body sections [98] and during mouse embryonic development [99]; for assessing the roles of TG1, TG3 and TG6 in keratinocyte differentiation (see [26] and references therein); and for detecting TG1, TG2 and FXIIIA activity during osteoclastogenesis [49,100], among other examples.
(2) Identification of Lys-donor substrates, which are incorporated into biotin-tagged peptides through specific endogenous TG activity. This strategy has been applied to TG1 and TG3 in differentiating cultured keratinocytes [101,102]; to TG6 in mouse epidermal extracts [97]; to TG2 and FXIIIA in osteoblastic cell extracts [103]; and to the identification of TG2 substrates in mouse renal glomeruli [104] as well as TG1/TG2 substrates in fibrotic mouse kidney [105] and liver tissues [106]. Notably, cytokeratins 8 and 18, established biomarkers of hepatocyte dysfunction, were identified as TG1/TG2 substrates. More recent mechanistic studies further revealed that TG2-mediated crosslinking of cytokeratin 18 with essential structural and regulatory proteins leads to aggregate formation, which may play a key role in apoptotic cell death [107]. Together, these findings suggest that targeting such TG2-mediated interactions may represent a potential therapeutic avenue for liver fibrosis. More generally, comprehensive functional and substrate-centered studies are required to uncover biologically relevant TG-mediated interactions that may be potential targets for therapeutic intervention in TG-related dysfunctions.
(3) Finally, TG1-specific K5 peptide has been used as a diagnostic tool for lamellar ichthyosis [108,109]. Further applications along this line are expected to be driven by recent methodological progress in the development of highly sensitive peptide-based biosensors for quantifying TG activity, which are also responsive to TG inhibitor treatment [110,111].

A further step forward in substrate profiling and characterization has recently been achieved through the use of ultra-high-throughput mRNA/cDNA display in combination with next-generation sequencing (NGS), for TG2 [112] and TG1 [113]. Briefly, combinatorial libraries comprising approximately 10^12^ variants, based on the Hitomi peptides T26 and K5, respectively, were generated and expressed using mRNA display technology. The resulting peptide-mRNA fusions were then subjected to TG activity-based selection and enrichment. Subsequent analysis of the corresponding cDNA sequences enabled the calculation of enrichment factors, which revealed amino acid preferences at specific positions and allowed the derivation of consensus motifs for each TG isozyme. In particular, for TG2 [112], the evolved sequences were used as query sequences in an NCBI Blastp search to identify potential protein targets. The analysis revealed matches within the variable and junction regions of immunoglobulins, suggesting a potential role of TG2 in the regulation of antibody function, consistent with previous findings showing that IgD molecules can act as TG2 substrates and undergo crosslinking either to themselves or to gluten-derived peptides [114]. Regarding TG1, its substrate profiling revealed already known substrates as well as novel potential targets such as cystatins, type II keratins and cornifin, among others. Further investigations are required to validate these findings *in vivo.*

In general, big data obtained by using this activity-based evolution of peptides platform are expected to support the development of future diagnostic and therapeutic strategies, as well as guide the selection of novel candidate proteins for *in vivo* validation as TG substrates.

### 1.6. Proteolytic Activation

To date, FXIII, TG1, TG3 and TG5 are the only members among hTGs known to undergo proteolytic cleavage to enhance enzyme activity, albeit with different processing pathways. As mentioned in Section 1.3, FXIII and TG1 show a close evolutionary relationship and display N-terminal extensions, clearly different in length and with distinct functional outcomes as a result of their distinct biological roles. A comparative overview of specific proteolytic activation mechanisms is provided in Table 1.

The blood coagulation factor FXIII can exist in two forms. The cellular form is a dimer, FXIIIA_2_, whose activation is proteolysis-independent and driven solely by increased intracellular Ca^2+^ concentrations [115]. By contrast, the circulating form of FXIII in plasma, that is, the plasmatic form, is an inactive heterotetramer (FXIIIA_2_B_2_), which is activated into the proteolyzed-FXIIIA_2_ catalytically competent form at the final stages of the coagulation cascade [46,116,117]. Its activation is a complex process that involves the cleavage of the activation peptide (AP) present in A subunits, comprising residues 1 to 17, by thrombin. This process facilitates Ca^2+^ binding, which in turn causes large conformational changes in FXIIIA subunit structure and also results in the dissociation of B subunits. The AP is, in fact, the major contributor to the dimeric interface of the zymogenic FXIIIA_2_, so its cleavage disrupts dimer stability dramatically [45], thus promoting dissociation as part of the activation process [116,118]. Indeed, inspection of the X-ray structure of FXIIIA_2_ (PDB ID: 1F13) reveals that the AP lies on the catalytic core domain of the opposite subunit, establishing specific interdomain hydrogen-bond interactions, thereby preventing access to the catalytic site. Recent mutational analyses have highlighted the specific role of AP residues in FXIII stability, susceptibility to thrombin cleavage, and TG activity [45,119,120].

TG1 is undoubtedly a unique transglutaminase in many respects. It is synthesized as a 92 kDa protein, being the longest hTG, and further post-translationally modified into a 106 kDa form [36]. TG1 may exist both in a cytosolic form and bound to the inner surface of the keratinocyte plasma membrane via myristyl and palmityl anchors, present at the N-terminal extension (residues 1–104) [121,122,123]. During keratinocyte differentiation, TG1 requires high Ca^2+^ concentration for catalytic activity and, interestingly, undergoes two proteolytic cleavages adjacent to Gly93 and Gly573, which coincide with the C-terminal boundaries of the membrane anchoring domain and the catalytic domain, respectively. As a result, TG1 is split into three fragments of 10, 67 and 33 kDa, corresponding to the membrane anchoring domain, the beta-sandwich and the catalytic domain, and the two beta-barrel domains (inactive), respectively [124].

The majority of TG1 in keratinocytes is bound to the membrane, either as an intact full-length form with low specific activity or as a highly active complex of 10/67/33 kDa interacting fragments. TG1 can also exist in multiple soluble forms, representing up to a third of the total TG1 activity in cultured keratinocytes. Thus, in addition to the full-length enzyme, the 67 kDa and 67/33 kDa proteolytically processed forms are present, which display 5–10-fold higher specific activities compared to the intact form [125,126,127]. TG1 proteolytic activation has also been recently detected in osteoclasts [49].

The identity of the specific proteases responsible for TG1 processing is still an open question, although cathepsin D and calpain are proposed as potential candidates. Thus, based on *in vitro* and *in vivo* studies [128], cathepsin D, a member of the aspartic protease family, has been proposed to play a crucial role in TG1 activation. Specifically, treatment of cultured keratinocytes with cathepsin D enhanced TG1 activity, while inhibiting cathepsin D suppressed TG1 activity. Moreover, cathepsin D-deficient mice showed reduced TG1 activity and alterations in the protein composition of the cornified envelope of the epidermis [128]. More recently, calpain small subunit 1 (CAPNS1), which forms heterodimers with the Ca^2+^-activated cysteine proteases calpain 1 and 2, has been identified as an interaction partner of TG1 in HEK293T cells, based on Virotrap screening experiments [129]. Furthermore, treatment of keratinocytes and transfected HEK293T cells with calpain inhibitors suppressed TG1 activity. The authors conclude that, rather than directly cleaving TG1, calpain may enhance its activity by modifying the surrounding protein environment, thus facilitating TG1-mediated crosslinking during cornified envelope formation.

Likewise, TG3 is expressed as a zymogen and requires proteolytic cleavage to become fully catalytically active. *In vitro* studies suggest that dispase, a bacterial protease, is able to proteolyze TG3, although the activation mechanism *in vivo* is not yet fully understood [130,131]. Cleavage occurs in the loop region comprising residues 461–473 (presumably at Ser469) between the catalytic and the two C-terminal β-barrel domains, resulting in two fragments of 47 and 30 kDa that remain noncovalently associated in the active enzyme. Cheng et al. [132] identified cathepsin L as the first mammalian protease capable of cleaving and activating TG3 *in vitro*, reporting the exact cleavage site between Ala466 and Ala467. Further studies are required to validate these results *in vivo*.

The complex resulting from TG3 proteolysis can bind two Ca^2+^ ions, in addition to the one acquired constitutively during TG3 expression. Recent structural studies by Heggelund et al. [74] have provided a deeper understanding of the conformational changes accompanying the activation mechanism of cleaved TG3, based on a crystal structure with an inhibitor irreversibly bound in the active site as a proxy substrate and the three bound Ca^2+^ ions. In this structure, the C-terminal domains are detached from the rest of the enzyme (see Section 2.1 for details). Moreover, structural rearrangement of a β-sheet at the C-terminal end of the catalytic domain facilitates the exposure of some of the residues involved in metal binding sites 2 and 3, relevant for enzyme activity.

TG5 has also been shown to be proteolytically processed in the baculovirus expression system and in mammal epithelial cells [133], resulting in two fragments: an inactive 28 kDa one, comprising the C-terminal regulatory (two β-barrels), and the N-terminal active 53 kDa fragment. As outlined above for other members of the family, the intact form of TG5 retains low specific activity, while the 53 kDa proteolytically processed fragment is highly active. The exact proteolytic site has not been precisely identified, although the loop region (Ser492–Ser501) has been suggested to be involved in the proteolytic process by analogy to TG3. The *in vitro* TG5 activation mechanism remains unclear, largely due to the fact that TG5’s atomic structure has not been resolved yet. It has been speculated, based on the modeled structure and by comparison to TG3, that Ca^2+^ could interact with the 53 kDa form, leading to a conformational change that exposes buried residues, including Trp241 and Trp333, thereby facilitating substrate access to the active site.

## 2. Structural Landscape of Human Transglutaminases

The structural information available for hTGs has grown significantly in recent years. However, this advance has been clearly unequal, both across individual family members and across the functional states captured experimentally. While some TGs have been characterized using complementary structural approaches, others remain largely unexplored despite their biological and pathological relevance. This heterogeneity directly shapes the mechanistic questions that can be addressed with structural support and necessitates a comparative and critical interpretation of the available evidence.

**Figure 4:**
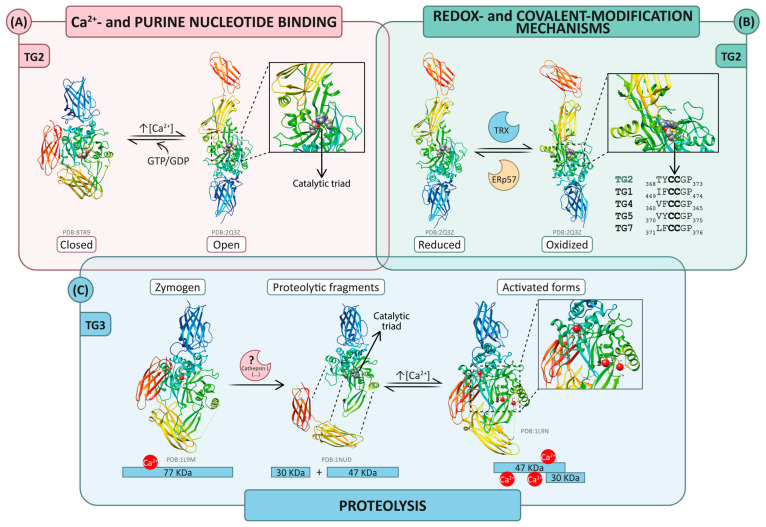
Multilayered regulation of hTGs. Transglutaminase activity is regulated through structurally encoded interdomain rearrangements and additional biochemical inputs. (**A**) Ca^2+^ and purine nucleotide binding shift the equilibrium between closed and open conformations, modulating active-site accessibility. However, catalytic competence is not determined by active-site exposure alone. (**B**) Redox state, covalent modifications and (**C**) proteolytic processing provide additional regulatory layers that can enhance or suppress activity without requiring major global rearrangements. Together, these mechanisms illustrate that activation across the family is multifactorial rather than strictly conformational. The corresponding Protein Data Bank (PDB) IDs are indicated next to each structural model. Question marks indicate that the proteases responsible for TG3 processing remain unidentified *in vivo*, although cathepsin L has been suggested *in vitro*.

In general terms, the experimental structures solved to date confirm a modular architecture conserved in the TG family. As previously mentioned, the common fold comprises a catalytic core flanked by a N-terminal β-sandwich and two C-terminal β-barrel domains and is captured in alternative interdomain arrangements associated with ligand binding, proteolytic processing or complex formation. In several structurally characterized members of the family, these transitions involve substantial repositioning of the C-terminal β-barrels relative to the catalytic core (e.g., after purine nucleotide binding in TG2) and are structurally linked to changes in active-site accessibility, supporting a model of interdomain allosteric regulation (see chapter 1 in [8,38,134]). In this sense, regulation in the TG family is structurally encoded within the architecture of the fold (Figure 4). However, exposure of the active site does not by itself define a catalytically competent state. Across the family, enzymatic activity additionally depends on biochemical determinants (including Ca^2+^ occupancy hierarchy, nucleotide binding, redox state, covalent modifications, and proteolytic processing) that are not necessarily captured in static structural snapshots [32,35,36]. Consistently, the best-resolved family members support a multi-site Ca^2+^ model in which distinct binding pockets contribute differently to stability and activation (e.g., three Ca^2+^ sites in FXIIIA and TG3), while other isoforms are inferred to share homologous sites but remain structurally unmapped [130,131,135].

Another recurring aspect is the presence of structurally flexible or poorly defined regions that consistently reappear in independent structures, even when different methods and crystallographic conditions are used. These dynamic regions are frequently located in peripheral loops and interdomain linkers, often overlapping with segments involved in regulatory transitions, consistent with intrinsic conformational variability rather than crystallographic artefacts. In this context, integrating complementary structural approaches has enabled characterization of biologically relevant conformations, despite persistent resolution constraints and conformational heterogeneity.

Overall, the current structural landscape of hTGs offers a solid basis for understanding general principles of folding and conformational regulation, but it also reveals significant gaps. The uneven availability of structures, the scarcity of catalytically complete states and the absence of key physiological complexes still limit a comprehensive structural description of the functional diversity of the protein family. These considerations provide the necessary framework for analyzing, in the following sections, what is known structurally about each hTG, what aspects remain unsolved and how these structural differences translate into specific biological functions. In addition, detailed information on all available Protein Data Bank (PDB) entries for hTGs is summarized in Table S1.

### 2.1. Structural Evidence Across Individual Human Transglutaminases

The experimental structural information available for human TG1 is very limited compared with other members of the family. To date, the only structure deposited in the PDB corresponds to 2XZZ, which describes an isolated C-terminal β-barrel domain of TG1 determined by X-Ray crystallography at 2.30 Å. The overall quality of the dataset is solid and supports a reliable description of the folding of this domain. However, its usefulness is limited, and it does not provide information on TG1 functional states or interdomain organization.

As a direct consequence of this experimental limitation, structural insight into TG1 has relied on predictive models, initially derived from homology with other better-characterized TGs such as FXIIIA or TG2 [36,134] and, more recently, on the AlphaFold structural model (AF-P22735-F1-v6) available in the AlphaFold Protein Structure Database [136,137]. These approaches consistently support a conserved modular architecture, although they also present important limitations. Inspection of the AlphaFold model indicates high local confidence in the central and C-terminal domains, whereas the N-terminal regions and interdomain loops show lower confidence and uncertain relative orientation. This interpretation is supported by predicted Local Distance Difference Test (pLDDT) confidence scores and Predicted Aligned Error (PAE) estimates provided by the AlphaFold framework. Consequently, these models are useful as general architectural references and for preliminary mapping of variants, but they do not replace experimental structural evidence. This situation highlights a notable structural gap for TG1 and underscores the need for experimental studies addressing the full-length protein under functionally relevant conditions.

TG2 is the most structurally characterized member of the hTG family and provides the clearest structural framework for understanding ligand-dependent regulation. Early purine nucleotide-bound structures (GDP: 1KV3; ATP: 3LY6; GTP: 4PYG) established a compact closed state in which C-terminal domains restrict access to the catalytic core [30,138,139]. Comparative analyses identified recurrently variable segments (e.g., 78–86, 406–412, 462–469) that remain poorly defined across methods, supporting the interpretation that they represent intrinsic mobile elements within TG2’s regulatory landscape. A decisive validation of the closed state as a solution-populated regulatory conformation comes from integrative cryo-EM/SAXS work [40], which confirmed that the closed TG2 conformation is not exclusive to the crystalline environment but represents a stable and biologically relevant regulatory state. The model also provides a coherent framework for interpreting the nucleotide-binding pocket, although the effective resolution limits the detailed description of individual atomic contacts.

In parallel, Ca^2+^-dependent activation of TG2 began to be addressed structurally through crystallographic studies conducted under Ca^2+^-enriched conditions. The first direct structural support comes from structure 6KZB [73], resolved at moderate resolution (~3.6 Å) in the presence of GDP. In this model, Ca^2+^-compatible density is identified in two defined regions of the protein, corresponding to one site in the catalytic domain and a second site in the first β-barrel domain, for which specific sets of coordinating residues are proposed. Functional analyses of these regions, together with earlier mutagenesis studies, support the existence of multiple Ca^2+^- binding sites (often described as S1–S5) in TG2, with differential contributions to transamidase activation and nucleotide regulation [73]. Although the resolution of the dataset does not allow a precise description of the coordination geometry, this structure provided an initial structural basis for discussing the localization of Ca^2+^ binding sites in TG2 and their relationship to the conformational equilibrium of the enzyme in a nucleotide-bound context.

More recently, the availability of structures corresponding to open and intermediate states has substantially expanded the conformational repertoire of TG2. The study by Sewa et al. [72] resolved TG2 structures in the presence of Ca^2+^ and peptidomimetic inhibitors (9BC2, 9BC3, and 9BC4), providing structural evidence that the transition from the closed to the open state can proceed through intermediate conformations stabilized under specific experimental conditions (see Section 1.4 for details). In the intermediate state, Ca^2+^ density is observed at structurally defined positions, whereas in fully open conformations, Ca^2+^ does not appear as a dominant organizing feature of the model. These observations support a model in which occupation of a subset of Ca^2+^ sites is sufficient to bias the conformational equilibrium, without requiring simultaneous saturation of all proposed Ca^2+^-binding pockets [72]. Taken together, these data establish TG2 as a system governed by a continuous conformational landscape rather than a strict binary switch. Conformational opening and catalytic competence are not strictly equivalent, as redox and covalent modifications further modulate activity independently of global domain rearrangement (Figure 4) [32,72].

Comparative analysis of crystallographic and cryo-EM structures reveals a consistently poor definition of the ~462–471 segment. This region is often absent in crystal structures and, although modelled in cryo-EM, it shows reduced Q-score and atom inclusion values relative to the catalytic core, supporting local flexibility or conformational heterogeneity. Predictive models such as AlphaFold (AF-P21980-F1-v6) may help contextualize regions lacking experimental coverage or assist in preliminary variant mapping, but they cannot substitute for experimental evidence when interpreting nucleotide- or Ca^2+^-regulated states.

Available structural data support a model in which TG2 regulation arises from a dynamic equilibrium between nucleotide-, Ca^2+^-, and ligand-modulated conformational states. The capture of closed, open, and intermediate structures establishes a continuous conformational landscape rather than a strict binary transition. However, key limitations remain, including the absence of catalytically complete structures with physiological substrates and the lack of atomic-level definition of Ca^2+^ coordination hierarchy. Addressing these gaps represents a priority for future integrative structural studies.

The first crystallographic structures of human TG3 (1L9M, 1NUD and related models) established its modular architecture, with the catalytic core flanked by the C-terminal β-barrels C1 and C2 [130,131]. In these conformations, C1/C2 associate with the catalytic core and restrict access to the active site, supporting a model of autoinhibition in which TG3 remains catalytically restrained until proteolysis and Ca^2+^ promotes activation (see Section 1.6 for details). Crystallographic analyses further demonstrate that TG3 contains three Ca^2+^ binding sites, with differential occupancy between the zymogen and activated states, supporting a stepwise Ca^2+^-dependent activation mechanism [130,131]. However, mechanistic insight is limited by recurrently unresolved regions near the catalytic center and proteolytic cleavage site.

A substantial advance in the structural understanding of TG3 occurred with the resolution of structures in complex with autoantibodies derived from patients with dermatitis herpetiformis (DH). The study by Heggelund et al. [74] resolved multiple TG3-Fab crystallographic complexes (8OXW, 8OXX, 8OXV and 8OXY), providing a direct characterization of conformational epitopes recognized by human autoantibodies. In these structures, the Fab fragments bind to exposed surfaces of the catalytic core and adjacent regions without inducing a global reorganization of the TG3 fold. The conformations observed are compatible with structurally inactive states previously described, indicating that immune recognition can occur on basal conformations of the enzyme and does not necessarily require a catalytically active state. Additionally, capture of an enzyme-substrate-intermediate-like state using the irreversible peptidomimetic inhibitor Z-DON as a substrate proxy added a key dimension to the TG3 conformational landscape [74]. Under these conditions, C1 and C2 are no longer defined in the crystallographic model, while the catalytic core undergoes coordinated rearrangement of active-site elements. The absence of C1/C2 density is more consistent with conformational decoupling or dynamic heterogeneity than with physical displacement. These structures establish a direct structural link between substrate-like occupation of the active site and global reorganization of TG3, indicating that activation reflects redistribution of conformational equilibria rather than a purely local event. This framework is expanded and partially clarified by subsequent structural work [140], in which structures of cleaved TG3 in complex with functionally activating autoantibodies (8RMX and 8RMY), both in the absence and presence of Z-DON. These structures correspond to a truncated construct lacking the C1/C2 C-terminal domains. Although the truncated constructs lack C1/C2 and therefore preclude direct assessment of their displacement, Fab binding stabilizes catalytic-core conformations compatible with increased catalytic competence. Taken together, these data support a model in which TG3 activation arises from global conformational readjustment involving functional decoupling of the C-terminal domains and stabilization of the catalytic core.

Despite recent advances, structural knowledge of TG3 remains incomplete in several key aspects. There is still no experimental structure of full-length TG3 that simultaneously captures the C1/C2 C-terminal domains and a catalytically active state with physiological substrates. Consequently, coupling between proteolysis, Ca^2+^ binding and interdomain reorganization remains indirectly inferred. Resolving this integration represents a central objective for future structural studies under near-physiological conditions.

Structural knowledge of human factor XIII has largely relied on studies of its catalytic subunit A (FXIIIA), the first hTG to be crystallized and the most structurally characterized member of the family. The first crystallographic structure of FXIIIA (1GGT, 1994) established the conserved global architecture of the enzyme and provided a framework for understanding the stability of the A_2_ dimer and the organization of the catalytic environment [141]. Subsequent studies confirmed this architecture and showed that activation involves conformational changes primarily affecting the relative arrangement of the C-terminal domains, although all structures were determined in the absence of the regulatory B subunit [142,143,144,145]. A key milestone was the resolution of activated FXIIIA in complex with a covalent inhibitor (4KTY) [82]. This structure captured, for the first time, an open conformation compatible with the catalytically active form of the enzyme and revealed the reorganization of the C-terminal β-barrels leading to active site exposure. Structurally, 4KTY provided a framework for describing FXIIIA activation as progressive domain rearrangement rather than global refolding.

This structural model was reinforced by additional crystallographic structures of FXIIIA in complex with different inhibitors (5MHL, 5MHM, 5MHN and 5MHO). Notably, regions of increased flexibility or reduced local definition recur across all these structures, including segments of the C-terminal region and loops proximal to the active site. Furthermore, the absence of subunit B represented an intrinsic limitation: while these models defined the architecture and local plasticity of FXIIIA, they did not directly address regulation within the physiological A_2_B_2_ complex.

This structural gap has recently begun to close by the resolution of the native A_2_B_2_ complex using cryo-EM (8CMT and 8CMU), at overall resolutions in the approximate range of 2.4–3.0 Å [75]. These structures reveal a compact FXIIIA_2_ dimer surrounded by an FXIIIB_2_ dimer organized around the catalytic core. Importantly, the A_2_ dimer, within the complex, closely resembles the conformation observed in isolated A_2_ structures. Thus, FXIIIB does not induce major pre-activation rearrangements but instead appears to regulate FXIIIA primarily through steric masking of regions involved in Ca^2+^ binding and thrombin cleavage (see Section 1.6 for details).

Together, these data provide a coherent structural framework for reinterpreting FXIII regulation in plasma. FXIIIB stabilizes a pre-activated A_2_ dimer and restricts access to key functional sites until thrombin cleavage and Ca^2+^ binding trigger activation. FXIIIA harbors three structurally defined Ca^2+^-binding sites (Cab1–Cab3), with Cab1 constitutively occupied in the zymogen and Cab2/Cab3 engaged during activation to promote the conformational changes associated with catalytic competence [135]. Mapping pathogenic mutations onto the A_2_B_2_ model further reinforces this framework. It allows phenotypic differences between mutations affecting A-A, A-B or B-B interfaces to be rationalized, an analysis not possible using isolated A_2_ structures alone. Nevertheless, major gaps remain. There is still no structure of the FXIIIA_2_B_2_ complex after proteolytic activation, nor is there a model of activated FXIIIA bound to fibrinogen. In addition, persistent flexibility of the C-terminal regions of FXIIIA and the distal sushi domains of FXIIIB continues to limit detailed understanding of substrate recognition and dynamic regulation.

### 2.2. Open Structural Challenges

Despite the structural advances achieved in recent years, a comparative analysis of the hTG family reveals several fundamental structural issues that remain unresolved.

First, multiple isoforms, particularly TG4, TG5, TG6, and TG7, still lack experimental structures entirely, even at the level of isolated domains. This gap severely limits direct structural interpretation of their regulation, specificity and pathological involvement, and forces reliance almost exclusively on predictive models, whose interdomain accuracy and biological relevance remain uncertain.

Second, the structural basis of Ca^2+^-dependent regulation remains incompletely defined. Although Ca^2+^ activation is broadly accepted across the family, structural evidence is uneven and conceptually fragmented. In TG2, multiple (at least five) Ca^2+^-binding sites have been proposed, yet their occupancy hierarchy and functional interdependence remain difficult to resolve structurally. In TG3 and FXIIIA, three Ca^2+^-binding sites have been structurally assigned, but their differential affinities, sequential engagement and mechanistic coupling to conformational transitions are not fully understood. A unifying framework distinguishing structural, regulatory and activation-associated Ca^2+^ sites across isoforms is still lacking, and the extent to which these sites are evolutionarily conserved or mechanistically specialized remains unclear.

Third, structures capturing catalytically complete states with physiological substrates are largely absent. The available structures largely correspond to inactive, preactivated, or artificially trapped states, frequently stabilized by covalent or peptidomimetic inhibitors. This limitation restricts direct structural insight into the catalytic cycle itself and into the recognition of native macromolecular substrates. Consequently, the coupling between activation, interdomain rearrangement, and catalysis continues to be inferred rather than structurally resolved.

Finally, recurrent flexible or poorly defined regions across TGs indicate that conformational dynamics are central to regulation, yet this dimension is insufficiently incorporated into current structural models. Addressing these open questions will require integrative structural approaches that combine high-resolution structures with solution-based and dynamic analyses to move beyond static snapshots toward mechanistic models that account for conformational populations and their modulation.

## 3. Human Diseases Associated with Transglutaminases

The TG family has been widely linked to a diverse spectrum of human pathologies. This section reviews the involvement of TGs in autoimmune disorders, rare Mendelian diseases, and highly prevalent acquired pathologies. An in-depth characterization is carried out in order to examine how specific alterations in each isoform (whether due to autoantibodies, germline mutations, or functional dysregulation) translate into defined pathological phenotypes, with differing levels of evidence and underlying mechanisms that depend on the tissue context and the disease.

### 3.1. Autoimmune Disorders Mediated by Transglutaminases

#### 3.1.1. Gluten-Induced Autoimmune Diseases

Gluten-related disorders constitute a common and increasingly recognized group of immune-mediated diseases that affect a substantial proportion of the global population. This term encompasses a spectrum of heterogeneous clinical manifestations triggered by gluten ingestion in genetically susceptible individuals, predominantly those carrying HLA-DQ2 or HLA-DQ8. Gluten is a complex mixture of hundreds of storage proteins, collectively known as prolamins, which occur naturally in certain cereals. Prolamins are characterized by a high content of glutamine and proline, conferring marked resistance to proteolytic degradation by digestive enzymes in the gastrointestinal tract [146]. In gluten-sensitive individuals, the intake of gluten can induce pathological adaptive immune responses not only against gluten-derived peptides but also against specific TGs, leading to autoimmune damage in multiple organs. These disorders therefore represent a form of gut-driven autoimmunity, in which immune responses initiated in the intestinal mucosa can manifest both locally in the small intestine and in distant tissues such as the skin and the central or peripheral nervous system [147]. Within this context, TGs have at least two key and interconnected functions. First, certain TG isoforms act as target autoantigens [12,13,14]. Second, TGs enzymatically deamidate partially digested gluten peptides, thereby increasing their immunostimulatory capacity and promoting the generation of anti-deamidated gliadin peptide antibodies (anti-DGP). This process is favored by the high glutamine content of gluten, which represents a preferred substrate for TGs.

The clinical phenotype of gluten-related autoimmunity strongly depends on the specific TG isoform targeted by the immune response. Consequently, isoform-specific autoantibodies act as clinically valuable biomarkers and as mechanistic indicators of the pathogenic pathways involved in specific clinical entities, such as anti-TG2 in celiac disease and anti-TG3 in dermatitis herpetiformis. In contrast, anti-TG6 has been proposed as a marker associated with gluten-related neurological disorders, but its use has not been implemented yet. Notably, these entities frequently co-occur, and diversification of anti-TG reactivity has been linked to epitope spreading within a sustained gluten-driven response, while maintaining substantial isoform specificity [148].

##### Celiac Disease (TG2)

Celiac disease (CeD) illustrates, as introduced in the previous section, how a single transglutaminase can act as both an enzyme and an autoantigen. In the small-intestinal lamina propria, catalytically active TG2 deamidates select glutamine residues within gliadin peptides, generating negatively charged epitopes that bind HLA-DQ2/DQ8 with high affinity and amplify CD4^+^ T-cell responses. TG2 can also transamidate gliadin to form TG2–gliadin complexes. These complexes are internalized by TG2-specific B cells and presented to gluten-reactive T cells by linked recognition, thereby initiating anti-TG2 autoantibody production and epitope spreading within the gluten–TG2 axis [149,150].

Clinically, CeD ranges from classic malabsorption (diarrhea, weight loss) to extraintestinal features such as iron-deficiency anemia, osteoporosis, infertility, and neurological complaints. However, many patients are detected by family or risk group screening rather than gastrointestinal symptoms. Global meta-analysis estimates ~1.4% seroprevalence and ~0.7% prevalence confirmed by biopsy, with geographic and demographic variation [150,151].

Diagnosis largely hinges on TG2 serology. The usual starting point is serum IgA anti-TG2 together with total IgA [14,152]. If the patient is IgA-deficient, IgG anti-TG2 and/or IgG anti-DGP tests are used [153]. Most of the activity is in the mucosa. Duodenal tissue can show subepithelial IgA anti-TG2 before villous damage, and ex vivo mucosal explants release anti-TG2. After starting a gluten-free diet, both signals decrease [154,155]. Additionally, these findings support the value of anti-TG2 for diagnosing potential or seronegative CeD. Usually, in adults, the medical pathways typically include duodenal biopsies to document enteropathy, whereas the pediatric guidelines allow a no-biopsy diagnosis when anti-TG2 titers are very high and strict criteria are met [156,157]. HLA-DQ2/DQ8 typing has a very high negative predictive value and is useful to exclude CeD in equivocal cases or after gluten withdrawal [158].

Therapeutic work is now starting to focus on TG2 and the immune cascade it drives [159,160]. In a randomized, double-blind gluten-challenge study, ZED1227, an oral TG2 inhibitor, reduced mucosal injury and improved symptoms compared with placebo [161]. Clinical pharmacology has confirmed intestinal target engagement at therapeutic doses [162]. Beyond histological and clinical endpoints, transcriptomic analyses of duodenal biopsies show that TG2 inhibition with ZED1227 largely suppresses the gluten-induced gene-expression program, including a dominant epithelial IFN-γ-associated signature, providing direct mechanistic evidence in humans [163]. Consistent with clinical trial data and recent integrative reviews, TG2 inhibition appears to mitigate, rather than fully prevent, gluten-induced epithelial injury [160,161,164]. An alternative strategy is currently in phase 2a testing: TAK-101 (gliadin encapsulated in poly(dl-lactide-co-glycolide) (PLGA) antigen nanoparticles) aims to induce antigen-specific tolerance and attenuate gluten-induced immune activation [165]. Similarly, the tight-junction modulator larazotide acetate improved symptoms during gluten challenge in a meta-analysis, yet the program has not succeeded in phase 3 [166]. Another immune-targeted approach is blockade of IL-15 with ordesekimab (AMG-714; PRV-015). In phase 2a trials, the primary histologic endpoints were not achieved, but improvements in symptoms, particularly diarrhea, and favorable intraepithelial lymphocyte (IEL) signals support continued development [167,168].

##### Dermatitis Herpetiformis (TG3)

Dermatitis herpetiformis (DH) (ORPHA: 1656) is a gluten-dependent blistering disorder in which TG3 is the principal cutaneous autoantigen [12,74,140,169]. In DH, anti-TG3 and anti-TG2 antibodies arise from defined plasma cell populations in the gut, with limited evidence of B-cell cross-reactivity, pointing to parallel, isoform-specific immune responses in gluten-sensitive individuals [169]. This organization of the immune response provides a clear example of a gut–skin axis, in which antibody responses generated in the intestinal mucosa ultimately drive pathogenic events in the skin. At the molecular level, TG3-directed antibodies recognize conformation-dependent epitopes that are exposed upon proteolytic activation. TG3-specific B-cell receptors (BCR) can internalize TG3–gluten complexes and present gluten peptides to CD4^+^ T cells (linked recognition). Moreover, recurrent TG3-reactive BCR can even enhance TG3 enzymatic activity, amplifying gluten deamidation and antigen presentation [74,140]. In the skin, granular IgA/TG3 deposits, within dermal papillae, trigger complement activation, neutrophil recruitment, and matrix-metalloproteinase release, supporting a plausible pathway from immune complex to subepidermal blistering [170]. Consistent with this gluten dependence, circulating anti-TG3 levels seem to decrease with a strict gluten-free diet [12].

Clinically, DH presents with intensely pruritic, symmetric papulovesicles on extensor surfaces and frequently coexists with small bowel enteropathy. In fact, CeD is diagnosed in the great majority (>90%) of patients with DH. In general, DH remains uncommon with a reported prevalence between 11 and 75 per 100,000 people in the United States and Europe [171,172]. A conclusive diagnosis of DH starts with a perilesional skin biopsy, for direct immunofluorescence (DIF), showing granular IgA in the dermal papillae [172,173]. In a large international multicenter study of DH, the best single serologic test was endomysial IgA (EMA), which targets TG2, detected by indirect immunofluorescence (IIF) on monkey esophagus. TG2-IgA ELISA demonstrated comparable diagnostic performance, while TG3-IgA ELISA was frequently positive but showed lower sensitivity and specificity overall. IgG assays added little. Currently, the most robust strategy of DH diagnosis integrates high-quality DIF with EMA/IIF and TG2-IgA ELISA, reserving TG3-IgA to increase sensitivity and add clinical specificity when the clinical picture requires it [172,174].

DH management centers on a strict gluten-free diet to suppress the driver antigen. Treatment with dapsone (or sulfasalazine/sulfapyridine) provides rapid symptomatic control but does not treat the underlying gluten-driven autoimmunity [172]. Although there are currently no clinical trials targeting TG3 or the TG3–BCR–gluten axis in humans, recent structural and immunologic advances propose these pathways as future therapeutic targets for the treatment of this disease [74,140].

##### Gluten-Related Neurological Diseases (TG6): Ataxia and Neuropathy

Gluten ataxia (GA) is an immune-mediated cerebellar ataxia in which TG6 is a leading candidate neuronal autoantigen, not yet a universally accepted diagnostic marker. Early prospective work reported anti-TG6 in ~73% of GA and ~32% of newly diagnosed CeD. Furthermore, anti-TG6 titers often decrease on a strict gluten-free diet, supporting their gluten dependence, although their specificity remains imperfect across different neurological cohorts [13,175,176]. Biologically, passive-transfer experiments have shown that patient-derived anti-TG antibodies, including those reactive to TG6, have been reported to induce transient ataxia in mice. This points to a direct antibody effect but also reveals cross-reactivity among isoforms (TG2/TG3/TG6), making it challenging to associate the effect exclusively with TG6 [177]. TG6, like other TGs, can also modify gluten peptides *in vitro*, providing a route to T-cell help and B-cell maturation in a cerebellar context, although *in vivo* cerebellar deamidation remains unproven [178]. However, tissue-level observations reinforce the key role of anti-TG6 antibodies in GA. In cerebrospinal fluid, increased IgA anti-TG6 has been reported, and CD138^+^ plasma cells have been documented in a subset of cases, supporting intrathecal and/or local humoral activity [179]. Neuropathological studies also show marked Purkinje cell loss, microglial activation, and lymphocytic infiltrates, pointing to both humoral and cellular immune mechanisms [180]. Clinically, many GA patients do not present with gastrointestinal symptoms, although some studies have shown that an early and strict gluten-free diet can stabilize or even improve objective cerebellar parameters (such as N-acetylaspartate measured by MR spectroscopy), supporting the view that this is a gluten-dependent and potentially modifiable process [181,182].

Apart from GA, gluten neuropathy (GN) extends this spectrum to the peripheral nervous system [13,176,183]. Case–control studies report increased anti-TG6 (often alongside celiac serology) in otherwise idiopathic axonal or painful sensory neuropathies. Additionally, in some studies, antibody titers decrease on a gluten-free diet, and symptomatic improvement has been reported in subsets [176,184]. However, not all studies have reached the same conclusion. A multicenter cross-sectional study found no difference in the prevalence of anti-TG6 or anti-TG2 antibodies between patients with idiopathic ataxia or neuropathy and healthy controls when using the manufacturer’s cut-off values. Only low-titer antigliadin signals appeared when thresholds were lowered, underscoring the heterogeneity of assays and the need for standardized methods with robust disease control groups [185]. In clinical practice, when GN is suspected, testing for celiac disease is recommended. Anti-TG6 can be considered a supportive marker rather than a stand-alone diagnostic, and in seropositive cases, a strict gluten-free diet may be tried as therapy.

Long-term follow-up studies highlight both the potential and the limitations of TG6 serology. In a cohort with more than five years of observation, prior IgA anti-TG6 was linked to regional brain atrophy, poorer physical function, and higher depression scores. Strict adherence to a gluten-free diet predicted more recent seronegativization, supporting TG6 as a disease-related and diet-responsive signal [183]. In contrast, the negative multicenter study mentioned earlier cautions against over-interpretation in the absence of standardized assays [185]. TG6 antibodies have also been described outside GA and GN (for example, in DH, where they typically fall with a gluten-free diet, and sporadically in other neurological contexts), so results should be interpreted in context and not as a stand-alone diagnostic tool [61,184,186]. Importantly, occasional detection of antigliadin antibodies in healthy adults showed no association with Magnetic Resonance Imaging (MRI) findings, cognition, or quality of life, reinforcing that older gliadin tests should not replace TG-centered assays [187]. In terms of treatment, a strict gluten-free diet remains the first-line approach, with immunotherapy reserved only for selected refractory cases. To date, no TG6-targeted interventions have entered clinical trials [183,185,188].

#### 3.1.2. Other Autoimmune Disorders Involving TGs

Beyond gluten-driven autoimmunity, immune responses involving TGs span a broad spectrum. At one end are diseases with a defined TG autoantigen and pathogenic antibodies, notably the acquired factor XIII A-subunit autoimmunity (AiF13D; ORPHA: 599513) and the Autoimmune Polyendocrine Syndrome type 1 (APS-1; ORPHA: 3453). AiF13D is a potentially life-threatening bleeding disorder that typically presents with normal routine coagulation tests, including prothrombin time (PT) and activated partial thromboplastin time (aPTT). Diagnosis relies on FXIII activity and antigen measurements, followed by inhibitor studies to confirm an immune-mediated mechanism. AiF13D typically responds to FXIII replacement combined with immunosuppression [189,190]. APS-1 is a monogenic autoimmune disorder caused by mutations in the autoimmune regulator (AIRE) gene and characterized by the breakdown of central immune tolerance and multiorgan autoimmunity. In adult male patients with APS-1, TG4 has been identified as a prostate-restricted autoantigen, with anti-TG4 antibodies occurring frequently in association with autoimmune prostatitis and male subfertility. Experimental studies in AIRE-deficient models recapitulate this phenotype, consistent with a link between impaired central tolerance and TG4-directed prostate autoimmunity. In this context, TG4 serves as a mechanistic autoantigen and research biomarker rather than a routine diagnostic target [54,191]. Current research in APS-1 spans targeted immunomodulatory approaches for severe organ-specific disease manifestations, and preclinical efforts are aimed at restoring AIRE-dependent immune tolerance [192,193].

There is another group of conditions in which anti-TG2 antibodies usually indicate coexisting CeD, rather than organ-specific autoimmunity in the affected tissue. This is most often encountered in type 1 diabetes (T1D), autoimmune thyroid disease (AITD), and primary Sjögren’s syndrome (pSS; ORPHA: 289390). Meta-analyses show that biopsy-confirmed CeD is more frequent in T1D and AITD, supporting the use of anti-TG2 as a case-finding tool in these settings rather than as a marker of tissue-directed TG2 autoimmunity [194,195,196]. Similarly, a large multicenter study reported increased CeD prevalence in pSS (and in diffuse systemic sclerosis), indicating that anti-TG2 positivity in pSS should prompt evaluation for CeD [197]. Major CeD guidelines also recognize these groups as higher-risk populations where targeted testing is considered [152]. In IgA nephropathy (IgAN; ORPHA: 34145), TG2 has been associated with pathogenic amplification pathways, and renal IgA anti-TG2 deposits have been described in CeD-linked subsets, although specificity can be context-dependent [198,199,200]. Finally, early exploratory findings, such as anti-TG6 antibodies reported in cohorts of amyotrophic lateral sclerosis (ALS; ORPHA: 803) or multiple sclerosis (MS), remain inconsistent and are not ready for clinical use until assays are standardized and validated against proper disease controls [61,63,201,202]. Table S2 provides an integrated overview of autoimmune and comorbid conditions in which TGs have been implicated, summarizing their proposed immune roles, strength of evidence, and clinical relevance.

### 3.2. Genetic Diseases Due to Mutations in TG Genes

Monogenic disorders linked to hTGs are rare, but they make biological sense: when crosslinking activity is lost, disease shows up in the tissue where that isoform normally works the most. TG1 loss in the cornified envelope causes autosomal recessive congenital ichthyosis (ARCI), classically lamellar ichthyosis (LI). TG3 loss in the inner root sheath causes uncombable hair syndrome type 2 (UHS2); TG5 deficiency in the superficial epidermis causes acral peeling skin syndrome (APSS). TG6 variants in cerebellar neurons have been linked to spinocerebellar ataxia type 35 (SCA35), though some variants need cautious interpretation. Finally, FXIIIA loss in plasma prevents proper fibrin stabilization and leads to congenital factor XIII A-subunit deficiency. By contrast, there are no validated Mendelian disorders for TG2, TG4 and TG7. The next paragraphs outline the phenotype, mechanism and translational status for each gene, and Table S3 provides a concise side-by-side summary.

#### 3.2.1. TGM1—Autosomal Recessive Congenital Ichthyosis (ARCI)

Alterations in *TGM1* are one of the most frequent causes of ARCI and the leading gene associated with lamellar ichthyosis (LI; ORPHA: 313), sometimes starting with a collodion-baby presentation. The clinical spectrum is wider and includes congenital ichthyosiform erythroderma (CIE; ORPHA:79394), thermosensitive bathing-suit ichthyosis (BSI; ORPHA:100976), and self-healing/self-improving collodion variants (SHCB/SICB; ORPHA:281122), sometimes limited to the acral areas (aSHCB; ORPHA:281127) [203,204,205]. In line with emerging gene-centric classifications of inherited cornification disorders, collectively termed epidermal differentiation disorders (EDDs), *TGM1*-associated non-syndromic EDDs may also be referred to as *TGM1-nEDD*, emphasizing the causal gene and phenotype while encompassing the traditional ARCI/LI spectrum [206,207].

ARCI is rare; Orphanet estimates a prevalence of 1–9 per million for ARCI overall and approximately 1 per 100,000–300,000 for LI in European populations. Despite its rarity, it is associated with a substantial clinical and quality-of-life burden [208]. The disease mechanism is a primary epidermal-barrier failure. Biallelic pathogenic mutations on *TGM1* lead to TG1 deficiency, which disrupts cornified-envelope assembly and keratinocyte differentiation, with a particular impairment of TG1-mediated protein crosslinking and insolubilization. A possible role of TG1 in esterification of ω-hydroxyceramides to involucrin, a key step in the formation of the corneocyte lipid envelope, may also be affected, although this function remains controversial [129]. Together, these defects provide a molecular basis for neonatal dehydration/collodion and lifelong scaling [15,129].

In ClinVar (early 2026), more than 200 *TGM1* mutations are classified as pathogenic or likely pathogenic (see Table S3 for details). Interestingly, thermosensitive missense alleles underlie the trunk-predominant distribution characteristic of BSI, reflecting the temperature-dependent instability of TG1 [209,210]. Translational data align with mechanisms. Current clinical management remains largely symptomatic, relying on intensive topical care and, in severe cases, systemic retinoids [211]. In contrast, TG1-directed approaches have shown clear proof-of-principle, as topical recombinant TG1 restored enzyme activity and barrier architecture in humanized TG1-deficient skin [212]. These advances have progressed toward clinical translation, with KB105 (a topical, replication-defective, non-integrating HSV-1-based vector delivering *TGM1*) currently in early clinical testing (phase I/II trial NCT04047732) with an expanded-access program (NCT05735158) [213]. In parallel, experimental platforms continue to expand, including a 2024 CRISPR-Cas9 *TGM1*-knockout human keratinocyte model developed for ex vivo and mechanistic studies, alongside broader therapeutic strategies reviewed elsewhere [214,215].

#### 3.2.2. TGM3—Uncombable Hair Syndrome Type 2 (UHS2)

Biallelic pathogenic variants in *TGM3* cause a recessive form of UHS2, characterized by dry, frizzy, spun-glass hair with triangular or reniform shafts and longitudinal grooves, which often improves with age. The original gene discovery study identified *PADI3* (peptidyl-arginine deiminase 3), *TGM3*, and *TCHH* (trichohyalin) as the three UHS2 genes and showed that *TGM3* loss-of-function variants abolish or severely reduce TG3 activity [216,217]. Orphanet classifies UHS2 as a rare hair-shaft disorder (ORPHA:1410) without a precise prevalence, consistent with the small number of families reported. In the largest cohort to date, most solved cases were explained by *PADI3*, while *TGM3* and *TCHH* accounted for a smaller proportion of cases [218]. Mechanistically, loss of TG3 impairs crosslinking of trichohyalin and keratin substrates within the inner root sheath and cortex, producing the characteristic shaft anomalies [25,219]. *TGM3*-knockout mice show cuticular defects, placing the primary defect in the hair-shaft structure rather than in the epidermal barrier [220]. Consistent with the rarity of UHS2, only a very limited number of pathogenic *TGM3* variants have been curated to date (see Table S3 for details). At present, there are no TG3-targeted or disease-modifying trials, so management is supportive and focused on gentle measures of hair care.

#### 3.2.3. TGM5—Acral Peeling Skin Syndrome (APSS)

APSS is caused by biallelic loss-of-function variants in *TGM5*. APSS is a recessive skin-fragility disorder with painless, superficial detachment of the stratum corneum (SC) at acral sites. Peeling often worsens with heat, humidity, and friction. Clinicopathologic studies localize the split to the stratum granulosum–stratum corneum (SG-SC) interface [221]. TG5 is a suprabasal keratinocyte enzyme responsible for crosslinking cornified-envelope proteins. Loss of TG5 function disrupts cohesion in the outer epidermis, consistent with the level of separation observed in patient skin [222,223]. This enzymatic deficiency has been proposed to increase epidermal protease activity and accelerate corneodesmosome breakdown, resulting in the superficial peeling that characterizes APSS [222]. Differential diagnoses include generalized peeling skin disease caused by mutations in the corneodesmosin gene (*CDSN*) and APSS-like phenotypes resulting from defects in cystatin A. However, the hallmark of classic APSS is its acral, non-scarring presentation in patients with confirmed *TGM5* variants [224,225,226].

Orphanet lists APSS as a rare disease with unknown prevalence (ORPHA:263534). The variant spectrum includes missense, frameshift, and splice-site changes. Previous reports have described a recurrent European founder allele (p.Gly113Cys) as well as different novel *TGM5* mutations. Larger multicenter series have expanded the allelic spectrum of *TGM5* and highlighted that APSS is frequently under-recognized and can be misclassified as other localized blistering/fragility disorders [221,227,228,229]. In ClinVar (early 2026), a limited number of *TGM5* submissions are classified as pathogenic/likely pathogenic, largely in the context of APSS, consistent with the rarity of the condition (see Table S3 for details). This highlights that only a small subset of alleles currently fulfills consensus pathogenicity criteria. APSS management remains supportive, centered on barrier repair and avoidance of heat, moisture, and friction. No TG5-targeted or disease-modifying therapies have been reported to date. However, in selected patients with sweat-exacerbated symptoms, intradermal botulinum toxin A has shown symptomatic benefit in case reports, likely by reducing palmar/plantar hyperhidrosis [230].

#### 3.2.4. TGM6—Spinocerebellar Ataxia Type 35 (SCA35)

Heterozygous pathogenic variants in *TGM6* have been linked to SCA35, an autosomal-dominant, adult-onset cerebellar ataxia. SCA35 is characterized by gait and limb incoordination, dysarthria, variable tremor, and cerebellar atrophy on brain MRI [231,232,233]. Orphanet classifies SCA35 (ORPHA:276193) as ultra-rare with a prevalence of <1/1,000,000 and autosomal-dominant inheritance, consistent with the small number of reported families. The *TGM6* reported variants are predominantly missense with occasional truncating alleles and cluster in functional regions of the enzyme [232,233,234]. Patient-derived mutations reduce TG6 activity, cause protein mislocalization, and activate the unfolded protein response and endoplasmic reticulum (ER) stress pathways, likely increasing Purkinje cell vulnerability [58,235]. Caution is needed in variant interpretation. Independent re-analyses have raised doubts about certain recurrent missense alleles, citing limited segregation data, modest gene-level constraint, and non-negligible population frequencies. These findings argue for careful evaluation of each variant, with co-segregation and functional evidence at the variant level, rather than a wholesale dismissal of the *TGM6*-SCA35 association [234,236]. Publicly available data on *TGM6* remain limited. Only a small number of pathogenic or likely pathogenic variants have been curated to date (see Table S3 for details), underscoring the need for variant-level interpretation integrating segregation and functional data. Because SCA35 is ultra-rare, no TG6-targeted or disease-modifying therapies have been reported to date, and management is supportive, following general standards for spinocerebellar ataxias [232,233].

#### 3.2.5. F13A1—Congenital Factor XIII A-Subunit Deficiency

Pathogenic variants affecting both alleles of the *F13A1* gene cause an exceptionally rare autosomal recessive bleeding disorder. Orphanet classifies congenital factor XIII deficiency (ORPHA:331) as an ultra-rare disorder, with an estimated prevalence of fewer than 1 in 1,000,000 individuals, consistent with the limited number of families reported worldwide and the enrichment of cases in populations with higher consanguinity rates. Diagnosis is often delayed because standard coagulation assays (PT/aPTT) are usually normal [237]. Early clinical signs include umbilical-stump bleeding in the neonatal period, with subsequent soft-tissue or intramuscular hemorrhage, impaired wound healing, and easy bruising. Women may also present with menorrhagia and recurrent pregnancy loss. The most serious complication is the markedly increased risk of life-threatening intracranial hemorrhage [52,238,239].

At the molecular level, FXIII functions in the terminal transglutaminase step of coagulation, catalyzing fibrin crosslinking and clot stabilization. Deficiency of the FXIIIA subunit results in mechanically unstable clots that are prone to delayed re-bleeding despite normal clot formation [52,240]. Accurate diagnosis requires direct measurement of FXIII activity, complemented by FXIIIA antigen testing when available, followed by genetic analysis to confirm *F13A1* involvement and to distinguish it from the much rarer F13B deficiency. Historical qualitative assays such as the urea clot-solubility test may suggest the diagnosis but lack sufficient sensitivity and are no longer recommended as stand-alone screening tools [52,237,241]. The genetic architecture of *F13A1* deficiency is heterogeneous, encompassing missense, nonsense, splice-site, and small insertion/deletion variants distributed across the gene. Clinical variant curation continues to expand as next-generation sequencing is increasingly applied in rare bleeding disorder cohorts [52,242].

Prophylaxis represents the standard of care, as severe bleeding may occur even at very low residual FXIII activity levels. Recombinant FXIIIA_2_ (catridecacog), administered at 35 IU/kg once monthly, maintains protective trough activity and has demonstrated excellent efficacy and tolerability across clinical trials, pediatric studies, and post-authorization cohorts [243]. Real-world data further indicate that pharmacokinetic-guided regimens allow individualized dosing and, in selected patients, extension of dosing intervals while preserving hemostatic protection [244,245]. Plasma-derived FXIII concentrates remain effective alternatives when the recombinant product is unavailable. Overall, current management strategies remain centered on replacement therapy to restore fibrin crosslinking, while gene-based approaches are still confined to preclinical investigation [246].

### 3.3. Protein Aggregation and Neurodegeneration Link to TGs

In several major neurodegenerative diseases, including Alzheimer’s, Parkinson’s, and Huntington’s, available evidence indicates that TG2 modulates the toxicity associated with misfolded proteins in a context-dependent manner, rather than acting as a direct determinant of aggregation. In Alzheimer’s disease, TG2 interacts with Aβ and tau and promotes the stabilization of oligomeric species through transamidation [42,247]. However, genetic studies in mouse models have shown that its elimination does not significantly reduce amyloid burden, indicating that its contribution lies not in regulating the deposition of pathological species but in modulating the persistence, molecular organization, and neurotoxic potential of aggregated species, with downstream effects on synaptic dysfunction, mitochondrial stress, and glial activation [248].

In Parkinson’s disease, the functional relationship between TG2 and α-synuclein is particularly consistent. Increased TG2 expression or activity promotes the formation of high-molecular-weight α-synuclein species associated with neurotoxicity, while genetic or functional reduction of TG2 decreases these pathological conformations and attenuates neurodegeneration in *in vivo* models [42,249]. In Huntington’s disease and other polyglutamine expansion disorders, a direct role for TG2 in huntingtin aggregation has been proposed [42,250]. However, subsequent cellular and genetic studies do not support TG2 as being necessary or sufficient for aggregate formation or phenotypic progression, compelling caution in making causal statements in this context [251,252].

Beyond TG2, other members of the family have been implicated in neurodegeneration, albeit with more limited evidence. In Alzheimer’s disease, isopeptide bonds have been detected in pathological lesions where a possible contribution of TG1 has been suggested, but without unequivocal enzyme assignment or genetic or functional support. Therefore, its role remains undetermined [253]. TG6, on the other hand, appears to display a distinct functional profile. As noted above, mutations in *TGM6* cause SCA35, and cellular data indicate that expression of wild-type TG6 can reduce α-synuclein levels and activate autophagic pathways, suggesting a possible modulatory or protective role in certain contexts [65]. However, interactions between TG6 and polyQ proteins, as well as increased insoluble fractions in overexpression-based cellular systems, including models expressing mutant huntingtin, have also been reported [60,254]. In the absence of *in vivo* genetic studies establishing necessity or sufficiency, these findings should be interpreted as associative and model-dependent, and do not allow TG6 to be assigned a firm causal role in polyQ aggregation.

From a therapeutic perspective, advances in the field have focused almost exclusively on TG2, which has been explored as a modifiable node in preclinical models of neurodegenerative disease. Experimental inhibition of TG2 in Alzheimer’s and Parkinson’s models has been reported to attenuate toxicity associated with misfolded proteins and related cellular dysfunctions, providing proof of principle that TG2 modulation can influence disease-relevant processes when effective inhibition is achieved within the central nervous system [42,247,249]. However, these approaches remain limited to preclinical and tool-compound settings and have not demonstrated sustained modification of disease course. Major challenges, including insufficient brain penetration, lack of validated biomarkers of target engagement, and the pleiotropic roles of TG2 in neuronal homeostasis, currently constrain the therapeutic exploitation of transglutaminase inhibition in neurodegeneration [42,255].

### 3.4. Cardiovascular Disease and Fibrosis

In cardiovascular disease, the involvement of the TG family is largely concentrated on TG2 and, in a distinct biological context, on FXIIIA. Available evidence indicates that both enzymes participate in relevant cardiovascular processes but with clearly differentiated functions.

TG2 contributes to cardiovascular remodeling through two complementary mechanisms. On the one hand, its Ca^2+^-dependent transamidase activity stabilizes fibronectin- and collagen-rich matrices through the formation of ε-(γ-glutamyl)-lysine crosslinks, thereby increasing vascular and myocardial stiffness and promoting diastolic dysfunction and elevated afterload in hypertension and heart failure [256]. On the other hand, independently of its catalytic activity, TG2 binds noncovalently to fibrillar fibronectin, with an affinity modulated by mechanical stress, contributing to the functional organization of the cell-matrix interface [257,258]. Together, these functions position TG2 as a molecular link between extracellular matrix (ECM) mechanics and adhesion-dependent cellular responses in cardiovascular remodeling.

TG2 activity is additionally modulated by redox-dependent mechanisms. Nitric oxide (NO) regulates both the localization and activity of TG2 in vascular cells, with increased NO levels promoting S-nitrosylation and limiting extracellular activity, whereas reduced NO availability favors TG2 association with the ECM, a phenomenon also observed in aged tissues characterized by increased arterial stiffness [259,260]. Consistent with a profibrotic role for TG2, pharmacological inhibition of the enzyme in preclinical models of pressure overload reduces interstitial fibrosis and improves diastolic parameters [261]. Similarly, in models of myocardial infarction, TG2 inhibition attenuates post-ischemic fibrosis and is associated with alterations in TGF-β1/Smad3 signaling, supporting a role for TG2 in stabilizing the fibrotic scar [260]. In line with its functional relevance in the vasculature, allosteric inhibition of TG2 has also been shown to lower blood pressure and improve endothelium-dependent vasodilation in resistance arteries [262]. Taken together, these observations indicate that TG2 can be pharmacologically modulated at different functional levels, while also emphasizing that its cardiovascular effects are highly context-dependent, contributing both to maladaptive matrix stiffening during chronic remodeling and to matrix stabilization during tissue repair [263].

In contrast to the multifaceted and matrix-centered role of TG2, FXIIIA plays a more restricted cardiovascular role linked to coagulation and tissue repair following myocardial infarction. As a plasma transglutaminase, FXIIIA stabilizes the fibrin network and the provisional matrix formed after ischemic injury, a critical step for effective myocardial healing. In murine models, FXIIIA deficiency results in ventricular rupture and defective scar formation after coronary ligation, whereas reconstitution of the factor restores structural integrity and myocardial repair, establishing a causal role for FXIIIA in post-infarction healing [264]. In humans, translational studies have linked FXIII activity to the quality of the repair process after infarction and have described a transient reduction during the acute phase, with potential prognostic relevance [265,266]. However, more recent analyses suggest that the association between plasma FXIII activity and clinical outcome weakens when broader determinants of health and tissue repair capacity are considered, indicating that FXIII primarily reflects the effectiveness of the healing response rather than acting as an independent therapeutic target [267].

From a broader fibrogenic perspective, TG2 also emerges as the transglutaminase with the strongest experimental support as a mediator of matrix stiffness and the maintenance of profibrotic states. In hepatic fibrosis, chronic liver injury is generally associated with increased TG2 expression and extracellular transglutaminase activity, consistent with a role in stabilizing collagen-rich scar matrices and reinforcing stiffness-dependent signaling in activated hepatic stellate cells [263,268]. A distinct increase in TG1 activity has also been reported in fibrotic liver tissue, although its functional contribution appears less clearly defined than that of TG2 [269]. Although evidence remains limited, FXIII has also been associated with hepatic fibrotic remodeling, as the FXIII-A Val34Leu variant has been linked to faster fibrosis progression in patients with chronic viral hepatitis, suggesting a potential context-dependent contribution of plasma transglutaminase activity to hepatic repair and matrix remodeling [270]. These observations are consistent with studies indicating that TG2 couples mechanical cues from the microenvironment to sustained activation of profibrotic programs, while a secondary contribution of TG1 has been described in specific contexts, including cardiac fibroblasts, where TG1 silencing reduces collagen insolubility and modulates connective tissue growth factor-linked profibrotic signaling [107,263]. In bleomycin-induced pulmonary fibrosis, FXIIIA-positive cells have been identified within fibroblastic foci, and genetic studies point to complementary, non-redundant roles for TG2 and FXIIIA in scar stability [271,272]. Consistent with these observations, TG2-driven ECM stabilization and sustained profibrotic signaling have also been described in renal fibrosis, reinforcing the concept of TG2 as a cross-organ mediator of fibrotic remodeling [263]. Notably, tissue-specific compensatory responses among TG isoforms complicate the interpretation of genetic and pharmacological studies, supporting tissue-specific compensatory responses and potential functional overlap within the family [263]. By contrast, the involvement of other TGs in fibrotic processes appears more limited and context-dependent, without reaching the level of functional evidence established for TG2.

### 3.5. Cancer

Altered expression of TGs is a recurring feature in different types of cancer. However, the strength and functional relevance of these associations vary markedly among different members of the family. In fact, although pan-cancer transcriptomic analyses frequently describe the dysregulation of several TGs, only a subset has robust mechanistic evidence directly linking protein alteration to tumor progression, cellular plasticity, or tumor microenvironment remodeling [38,273,274,275].

TG2 is by far the transglutaminase with the most consistent evidence in cancer, yet its contribution is markedly context-dependent and does not respond to a single dominant mechanism. According to the accumulated evidence, the relationship between TG2 and cancer is organized into two non-exclusive functional levels. This duality is articulated, on the one hand, in a regulatory function of cell signaling, linked to non-catalytic conformational states and protein–protein interactions, and, on the other hand, in an extracellular enzymatic function involved in the remodeling of the tumor microenvironment through the modification and stabilization of ECM components and ECM-dependent adhesion circuits [38,276]. This framework helps to rationalize why TG2 is recurrently associated with aggressive phenotypes without implying a uniform role in all tumors, reflecting a marked dependence on the tumor and stromal context [277,278]. From a clinical perspective, increased TG2 expression has been recurrently associated with poorer prognosis in solid tumors, although a substantial portion of the evidence comes from expression associations and preclinical models [279]. Moreover, TG2 is one of the strongest inducers of the Epithelial-to-Mesenchymal Transition. By promoting the expression of transcription factors like Snail, Twist, and Zeb1, TG2 allows differentiated cancer cells to “de-differentiate” back into a stem-like state. This plasticity makes the tumor far more resilient to treatment [280,281]. These insights have motivated therapeutic strategies aimed at direct TG2 modulation, including covalent active-site inhibitor programs designed to lock the enzyme in an open conformation and suppress TG2-dependent signaling in cellular contexts [164,280,282,283]. Finally, recent mechanistic and perspective work has proposed a role for TG2 in adaptive stress responses, including TG2-linked autophagy programs implicated in acquired resistance [284].

TG1 is repeatedly identified in pan-cancer transcriptomic analyses as a marker associated with prognosis and tumor immune composition; however, these associations are strongly conditioned by epithelial differentiation and cell composition, which limits their direct functional interpretation [273]. In specific epithelial carcinomas, experimental studies indicate that TG1 may promote cell survival and more adaptive cell states, although these effects are not consistent across tumor entities and do not define a uniform oncogenic or suppressor role [8,285]. Within this framework, recent mechanistic work demonstrates that TG1 and TG3 directly mediate specific transamidation reactions that facilitate metastatic behavior in defined molecular contexts [286]. TG3, on the other hand, shows a more consistent profile in epithelial tumors. Pan-cancer analyses indicate that its expression is reduced in multiple tumor entities and is associated with clinical and prognostic parameters, largely based on correlative analyses [274]. Consistently, functional studies in epithelial carcinomas demonstrate that TG3 restricts proliferation and invasiveness and attenuates programs associated with epithelial–mesenchymal transition, in line with its physiological role in terminal differentiation [287,288].

TG4 represents a highly tissue-restricted member of the family, with expression largely confined to the prostate. In prostate cancer, interest in *TGM4* has focused primarily on its value as a prostate-specific biomarker, identified in proteomic studies of fluids and useful in diagnostic and stratification contexts [53,289,290]. In addition, its tissue specificity has led to its evaluation as a tumor-associated antigen with translational potential in the development of targeted immunotherapeutic strategies [54].

FXIIIA is a distinct case within the family, with functions predominantly associated with the tumor microenvironment. Transcriptomic and single-cell studies have identified consistent expression of FXIIIA in myeloid subpopulations, particularly tumor-associated macrophages, supporting its use as a marker of specific stromal and immune states [275,291]. At the functional level, the strongest evidence links FXIIIA to fibrin-dependent circuits, which may promote tumor invasion and dissemination in defined contexts, without implying a decisive role in primary tumor growth [292,293].

For the rest of the transglutaminases, particularly TG5 and TG6, the evidence available in cancer is currently indirect and limited. In the case of TG5, genetic associations between regulatory variants of the *TGM5* locus and susceptibility to lung cancer have been described, without demonstration of a direct functional role of the enzyme in tumor progression [294,295]. For TG6, its involvement is mainly limited to transcriptomic observations and the description of a germline mutation in a familial context of acute myeloid leukemia [62,296]. TG7, meanwhile, has recently begun to appear in oncological studies. Thus, its overexpression has been described in testicular germ cell tumors, and it has been shown that its silencing reduces tumor viability and proliferation *in vitro*, supporting its consideration as an emerging candidate in this specific context [67].

## 4. Future Perspectives

The transglutaminase field is entering a phase in which expanding structural and biochemical knowledge can be translated into deeper mechanistic and biomedical insight. Despite significant advances, important questions remain open regarding how individual TG isoforms are activated and regulated under native conditions. For several members of the family (particularly TG3, TG4, TG5, TG6 and TG7), fundamental aspects such as their physiological roles, involvement in metabolic pathways, and, importantly, their *in vivo* interactors remain poorly understood. Substrate-centered proteomics, interactome mapping and quantitative activity-based profiling, combined with targeted biochemical and biophysical analyses, will be instrumental in resolving these gaps.

In parallel, the limited availability of high-resolution structural characterization for less-characterized TG isoforms currently constrains our understanding of their activation mechanisms and ligand interactions. Future work combining cryo-EM, integrative structural biology, and advanced biochemical analyses will be essential to clarify how domain organization and calcium binding coordinate conformational transitions across the family. Beyond structural resolution, defining how regulatory inputs are integrated within distinct cellular environments represents a major challenge. TG2 exemplifies ligand-dependent conformational switching, yet it remains unclear whether additional TGs harbor unrecognized regulatory sites, context-specific interaction partners or non-catalytic functions that shape their activity. Dissecting these layers of control will be essential to understand how TG regulation operates in complex cellular settings. Such knowledge may enable more selective pharmacological modulation, particularly through strategies that stabilize defined conformational states rather than simply blocking catalytic activity. The development of isoform-specific modulators with appropriate pharmacological profiles therefore remains an important unmet goal.

Future research should also address key disease-oriented questions. The molecular triggers that initiate anti-TG autoimmunity beyond gluten-driven disorders remain incompletely understood, as does the contribution of TG-mediated crosslinking to protein aggregation and toxicity in neurodegenerative contexts. In complex diseases such as cancer and fibrosis, identifying isoform-specific substrates and signaling networks will be critical to distinguish primary pathogenic drivers from secondary consequences. The development of isoform-selective modulators, especially those that stabilize defined conformational states, remains an important translational goal. In parallel, innovative strategies for congenital TG deficiencies, including gene-based therapies and targeted enzyme replacement such as topical TG1 delivery for inherited ichthyosis, deserve further investigation.

In summary, the next phase of TG research will require integrating mechanistic resolution with disease-oriented investigation and therapeutic innovation. Systematic dissection of isoform-specific regulatory architectures and substrate repertoires, together with rigorous functional validation, will be essential to better define the therapeutic opportunities associated with hTGs.

## Figures and Tables

**Table 1:** Proteolytic activation mechanisms of hTGs.

TG Isoform	Zymogen/Inactive Form	Cleavage Region	Protease(s)	Active Fragments	Biological Context
FXIII (plasma)	FXIIIA_2_B_2_ heterotetramer	Activation peptide (AP), residues 1–17	Thrombin	Proteolyzed FXIIIA_2_ after B-subunit dissociation	Activation in the final stages of the coagulation cascade. Ca^2+^ binding induces conformational changes that expose the catalytic site
TG1	Full-length 92 kDa enzyme (post-translationally modified to ~106 kDa). Membrane-associated via N-terminal lipid anchors or cytosolic	Cleavages near Gly93 and Gly573	Proposed: cathepsin D, possibly calpain-dependent regulation	10 kDa (membrane anchor), 67 kDa (β-sandwich + catalytic domain), 33 kDa (β-barrel domains). Remain associated in the active complex	Proteolytic processing generates highly active TG1 complexes during keratinocyte differentiation and cornified envelope formation
TG3	Inactive zymogen	Loop 461–473 (between catalytic domain and β-barrels), around Ser469. Alternative cleavage Ala466–Ala467	Dispase (*in vitro*). Mammalian candidate: cathepsin L	47 kDa and 30 kDa fragments (noncovalently associated)	Proteolysis enables Ca^2+^-dependent conformational rearrangements required for catalytic activation in epidermal tissues
TG5	Full-length enzyme with low activity	Proposed loop Ser492–Ser501	Not identified	53 kDa active N-terminal fragment + 28 kDa inactive C-terminal fragment	Proteolytic processing increases catalytic activity in epithelial cells. Structural details remain uncertain due to the lack of experimental TG5 structures

## Data Availability

No new data were created or analyzed in this study. Data sharing is not applicable to this article.

## Supplementary Materials

The following supporting information can be downloaded at: https://www.mdpi.com/article/10.3390/ijms27072976/s1. Refs. [297,298,299,300,301,302] are cited in Supplementary Materials.

## References

[1] Griffin M., Casadio R., Bergamini C.M. (2002). Transglutaminases: Nature’s biological glues. Biochem. J..

[2] Alshehri F.S.M., Whyte C.S., Mutch N.J. (2021). Factor XIII-A: An Indispensable “Factor” in Haemostasis and Wound Healing. Int. J. Mol. Sci..

[3] Boateng N.K.K., Wimberley R., Rose J., D’Alessandro A., Cohen M., Moore E., Schmitt L., Poole L., Luyendyk J., Hansen K. (2026). Tissue transglutaminase drives fibrin beta-chain cross-linking: A novel fibrin modification observed in patients with trauma. Blood.

[4] Awan M., Papez M., Walvekar A.P., Lee S.J., Dasbiswas K., Ramasubramanian A.K. (2025). Surface-bound FXIII enhances deposition and straightness of fibrin fibers. Biophys. Rep..

[5] Ramanujam R.K., Lavi Y., Poole L.G., Bassani J.L., Tutwiler V. (2025). Understanding blood clot mechanical stability: The role of factor XIIIa-mediated fibrin crosslinking in rupture resistance. Res. Pract. Thromb. Haemost..

[6] Luyendyk J.P., Flick M.J., Wolberg A.S. (2025). Factor XIII: Driving (cross-)links in hemostasis, thrombosis, and disease. Blood.

[7] Lorand L., Graham R.M. (2003). Transglutaminases: Crosslinking enzymes with pleiotropic functions. Nat. Rev. Mol. Cell Biol..

[8] Ebrahimi Samani S., Tatsukawa H., Hitomi K., Kaartinen M.T. (2024). Transglutaminase 1: Emerging Functions beyond Skin. Int. J. Mol. Sci..

[9] Gutierrez-Cerrajero C., Sprecher E., Paller A.S., Akiyama M., Mazereeuw-Hautier J., Hernandez-Martin A., Gonzalez-Sarmiento R. (2023). Ichthyosis. Nat. Rev. Dis. Primers.

[10] Song Y., Kirkpatrick L.L., Schilling A.B., Helseth D.L., Chabot N., Keillor J.W., Johnson G.V., Brady S.T. (2013). Transglutaminase and polyamination of tubulin: Posttranslational modification for stabilizing axonal microtubules. Neuron.

[11] Martucciello S., Sposito S., Esposito C., Paolella G., Caputo I. (2020). Interplay between Type 2 Transglutaminase (TG2), Gliadin Peptide 31-43 and Anti-TG2 Antibodies in Celiac Disease. Int. J. Mol. Sci..

[12] Kaunisto H., Salmi T., Lindfors K., Kemppainen E. (2022). Antibody Responses to Transglutaminase 3 in Dermatitis Herpetiformis: Lessons from Celiac Disease. Int. J. Mol. Sci..

[13] Velikova T., Vasilev G., Shumnalieva R., Chervenkov L., Miteva D.G., Gulinac M., Priftis S., Lazova S. (2024). Autoantibodies related to ataxia and other central nervous system manifestations of gluten enteropathy. World J. Clin. Cases.

[14] Iversen M.N., Stribolt K., Hvas C.L., Dige A. (2025). Diagnostic accuracy of IgA anti-tissue transglutaminase for the diagnosis of coeliac disease. Dan. Med. J..

[15] Nemes Z., Marekov L.N., Fesus L., Steinert P.M. (1999). A novel function for transglutaminase 1: Attachment of long-chain omega-hydroxyceramides to involucrin by ester bond formation. Proc. Natl. Acad. Sci. USA.

[16] Rawlings A.V., Lane M.E., Voegeli R. (2024). The importance of stratum corneum omega-linoleoyloxyacylceramides in human skin barrier health: Their biochemistry, processing enzymes and metabolites involved in corneocyte lipid envelope maturation. Int. J. Cosmet. Sci..

[17] Zhang Y.S., Benjami K. (2024). Transglutaminase: Fundamentals and Applications.

[18] Duarte L., Matte C.R., Bizarro C.V., Ayub M.A.Z. (2019). Review transglutaminases: Part II-industrial applications in food, biotechnology, textiles and leather products. World J. Microbiol. Biotechnol..

[19] Savoca M.P., Tonoli E., Atobatele A.G., Verderio E.A.M. (2018). Biocatalysis by Transglutaminases: A Review of Biotechnological Applications. Micromachines.

[20] Duarte L., Matte C.R., Bizarro C.V., Ayub M.A.Z. (2020). Transglutaminases: Part I-origins, sources, and biotechnological characteristics. World J. Microbiol. Biotechnol..

[21] Kolotylo V., Piwowarek K., Kieliszek M. (2023). Microbiological transglutaminase: Biotechnological application in the food industry. Open Life Sci..

[22] Doti N., Caporale A., Monti A., Sandomenico A., Selis F., Ruvo M. (2020). A recent update on the use of microbial transglutaminase for the generation of biotherapeutics. World J. Microbiol. Biotechnol..

[23] Lerner A., Benzvi C., Vojdani A. (2025). The Frequently Used Industrial Food Process Additive, Microbial Transglutaminase: Boon or Bane. Nutr. Rev..

[24] Esposito C., Caputo I. (2005). Mammalian transglutaminases. Identification of substrates as a key to physiological function and physiopathological relevance. FEBS J..

[25] Chermnykh E.S., Alpeeva E.V., Vorotelyak E.A. (2020). Transglutaminase 3: The Involvement in Epithelial Differentiation and Cancer. Cells.

[26] Teshima H., Kato M., Tatsukawa H., Hitomi K. (2020). Analysis of the expression of transglutaminases in the reconstructed human epidermis using a three-dimensional cell culture. Anal. Biochem..

[27] Matsuki M., Yamashita F., Ishida-Yamamoto A., Yamada K., Kinoshita C., Fushiki S., Ueda E., Morishima Y., Tabata K., Yasuno H. (1998). Defective stratum corneum and early neonatal death in mice lacking the gene for transglutaminase 1 (keratinocyte transglutaminase). Proc. Natl. Acad. Sci. USA.

[28] Tatsukawa H., Hitomi K. (2021). Role of Transglutaminase 2 in Cell Death, Survival, and Fibrosis. Cells.

[29] Yao Z., Fan Y., Lin L., Kellems R.E., Xia Y. (2024). Tissue transglutaminase: A multifunctional and multisite regulator in health and disease. Physiol. Rev..

[30] Liu S., Cerione R.A., Clardy J. (2002). Structural basis for the guanine nucleotide-binding activity of tissue transglutaminase and its regulation of transamidation activity. Proc. Natl. Acad. Sci. USA.

[31] Beninati S., Piacentini M., Bergamini C.M. (2017). Transglutaminase 2, a double face enzyme. Amino Acids.

[32] Navals P., Rangaswamy A.M.M., Kasyanchyk P., Berezovski M.V., Keillor J.W. (2024). Conformational Modulation of Tissue Transglutaminase via Active Site Thiol Alkylating Agents: Size Does Not Matter. Biomolecules.

[33] Zhuang R., Khosla C. (2020). Substrates, inhibitors, and probes of mammalian transglutaminase 2. Anal. Biochem..

[34] Kanchan K., Fuxreiter M., Fesus L. (2015). Physiological, pathological, and structural implications of non-enzymatic protein-protein interactions of the multifunctional human transglutaminase 2. Cell Mol. Life Sci..

[35] Eckert R.L., Kaartinen M.T., Nurminskaya M., Belkin A.M., Colak G., Johnson G.V., Mehta K. (2014). Transglutaminase regulation of cell function. Physiol. Rev..

[36] Klock C., Khosla C. (2012). Regulation of the activities of the mammalian transglutaminase family of enzymes. Protein Sci..

[37] Gundemir S., Colak G., Tucholski J., Johnson G.V. (2012). Transglutaminase 2: A molecular Swiss army knife. Biochim. Biophys. Acta.

[38] Zaltron E., Vianello F., Ruzza A., Palazzo A., Brillo V., Celotti I., Scavezzon M., Rossin F., Leanza L., Severin F. (2024). The Role of Transglutaminase 2 in Cancer: An Update. Int. J. Mol. Sci..

[39] Buccarelli M., Castellani G., Fiorentino V., Pizzimenti C., Beninati S., Ricci-Vitiani L., Scattoni M.L., Mischiati C., Facchiano F., Tabolacci C. (2024). Biological Implications and Functional Significance of Transglutaminase Type 2 in Nervous System Tumors. Cells.

[40] Aplin C., Zielinski K.A., Pabit S., Ogunribido D., Katt W.P., Pollack L., Cerione R.A., Milano S.K. (2024). Distinct conformational states enable transglutaminase 2 to promote cancer cell survival versus cell death. Commun. Biol..

[41] Liao Y.W., Hsieh H.H., Yeh J.W., Wang H.C., Huang S.Y., Wang P.Y., Lin J.J. (2025). Transglutaminase 2-mediated glutamine deamidation enhances p21 stability during senescence. Proc. Natl. Acad. Sci. USA.

[42] Liu J., Mouradian M.M. (2024). Pathogenetic Contributions and Therapeutic Implications of Transglutaminase 2 in Neurodegenerative Diseases. Int. J. Mol. Sci..

[43] Ha K.S. (2024). Transglutaminase 2 in diabetes mellitus: Unraveling its multifaceted role and therapeutic implications for vascular complications. Theranostics.

[44] O’Day D.H. (2024). The Search for a Universal Treatment for Defined and Mixed Pathology Neurodegenerative Diseases. Int. J. Mol. Sci..

[45] Syed Mohammed R.D., Gutierrez Luque L., Maurer M.C. (2024). Factor XIII Activation Peptide Residues Play Important Roles in Stability, Activation, and Transglutaminase Activity. Biochemistry.

[46] Dull K., Fazekas F., Torocsik D. (2021). Factor XIII-A in Diseases: Role Beyond Blood Coagulation. Int. J. Mol. Sci..

[47] Byrnes J.R., Lee T., Sharaby S., Campbell R.A., Dobson D.A., Holle L.A., Luo M., Kangro K., Homeister J.W., Aleman M.M. (2024). Reciprocal stabilization of coagulation factor XIII-A and -B subunits is a determinant of plasma FXIII concentration. Blood.

[48] Zunic M., Vreca N., Bevc S. (2024). The role of factor XIII in patient blood management. Blood Coagul. Fibrinolysis.

[49] Ebrahimi Samani S., Kaartinen M.T. (2023). Increased Osteoclastogenesis in Absence of TG2 Is Reversed by Transglutaminase Inhibition-Evidence for the Role for TG1 in Osteoclast Formation. Cells.

[50] Sun H., Kaartinen M.T. (2018). Transglutaminase activity regulates differentiation, migration and fusion of osteoclasts via affecting actin dynamics. J. Cell Physiol..

[51] Jacobs J.W., Booth G.S., Costa V., Figueroa Villalba C.A., Savani B.N., Adkins B.D. (2026). Factor XIII Deficiency: A Review of Biology, Testing, and Treatment. Clin. Hematol. Int..

[52] Dorgalaleh A., Jozdani S., Zadeh M.K. (2025). Factor XIII Deficiency: Laboratory, Molecular, and Clinical Aspects. Semin. Thromb. Hemost..

[53] Ye L., Sanders A.J., Jiang W.G. (2023). Transglutaminase-4 (Prostate Transglutaminase), a Potential Biological Factor and Clinical Indicator for the Diagnosis and Prognosis of Prostate Cancer. Anticancer Res..

[54] Lopez-Bujanda Z.A., Obradovic A., Nirschl T.R., Crowley L., Macedo R., Papachristodoulou A., O’Donnell T., Laserson U., Zarif J.C., Reshef R. (2021). TGM4: An immunogenic prostate-restricted antigen. J. Immunother. Cancer.

[55] Savoca M.P., Inferrera A., Verderio E.A.M., Caccamo D. (2019). Search for Novel Diagnostic Biomarkers of Prostate Inflammation-Related Disorders: Role of Transglutaminase Isoforms as Potential Candidates. Mediat. Inflamm..

[56] Csoban-Szabo Z., Becsi B., El Alaoui S., Fesus L., Korponay-Szabo I.R., Kiraly R. (2021). Biochemical Characterisation of Human Transglutaminase 4. Int. J. Mol. Sci..

[57] Thomas H., Beck K., Adamczyk M., Aeschlimann P., Langley M., Oita R.C., Thiebach L., Hils M., Aeschlimann D. (2013). Transglutaminase 6: A protein associated with central nervous system development and motor function. Amino Acids.

[58] Tripathy D., Vignoli B., Ramesh N., Polanco M.J., Coutelier M., Stephen C.D., Canossa M., Monin M.L., Aeschlimann P., Turberville S. (2017). Mutations in TGM6 induce the unfolded protein response in SCA35. Hum. Mol. Genet..

[59] Cascella N.G., Santora D., Gregory P., Kelly D.L., Fasano A., Eaton W.W. (2013). Increased prevalence of transglutaminase 6 antibodies in sera from schizophrenia patients. Schizophr. Bull..

[60] Guan W.J., Xia K.D., Ma Y.T., Liu Y.T., Shi Y.T., Jiang H., Shen L., Xia K., Li J.D., Tang B.S. (2013). Transglutaminase 6 interacts with polyQ proteins and promotes the formation of polyQ aggregates. Biochem. Biophys. Res. Commun..

[61] Gadoth A., Nefussy B., Bleiberg M., Klein T., Artman I., Drory V.E. (2015). Transglutaminase 6 Antibodies in the Serum of Patients With Amyotrophic Lateral Sclerosis. JAMA Neurol..

[62] Pan L.L., Huang Y.M., Wang M., Zhuang X.E., Luo D.F., Guo S.C., Zhang Z.S., Huang Q., Lin S.L., Wang S.Y. (2015). Positional cloning and next-generation sequencing identified a TGM6 mutation in a large Chinese pedigree with acute myeloid leukaemia. Eur. J. Hum. Genet..

[63] Cristofanilli M., Gratch D., Pagano B., McDermott K., Huang J., Jian J., Bates D., Sadiq S.A. (2017). Transglutaminase-6 is an autoantigen in progressive multiple sclerosis and is upregulated in reactive astrocytes. Mult. Scler..

[64] Zheng R., Li Z., He F., Liu H., Chen J., Chen J., Xie X., Zhou J., Chen H., Wu X. (2018). Genome-wide association study identifies two risk loci for tuberculosis in Han Chinese. Nat. Commun..

[65] Chen K., Lu Y., Peng F., Yu H.L., Wu J.Y., Tan Y., Zhao Y.X. (2020). TGM6 variants in Parkinson’s disease: Clinical findings and functional evidence. J. Integr. Neurosci..

[66] Kuramoto K., Yamasaki R., Shimizu Y., Tatsukawa H., Hitomi K. (2013). Phage-displayed peptide library screening for preferred human substrate peptide sequences for transglutaminase 7. Arch. Biochem. Biophys..

[67] Altuwayjiri R.S., Almami I.S. (2025). Transglutaminase 7 Silencing Inhibits Proliferation and Modulates Inflammatory and Apoptotic Markers in Testicular Germ Cell Tumors. Oncol. Res..

[68] Lorand L., Murthy S.N., Parameswaran K.N., Velasco P.T., Wilson J. (1992). Amide bond cleavage monitored continuously through detection of a dansylcadaverine leaving group. Biochem. Biophys. Res. Commun..

[69] Murzin A.G., Brenner S.E., Hubbard T., Chothia C. (1995). SCOP: A structural classification of proteins database for the investigation of sequences and structures. J. Mol. Biol..

[70] Grenard P., Bates M.K., Aeschlimann D. (2001). Evolution of transglutaminase genes: Identification of a transglutaminase gene cluster on human chromosome 15q15. Structure of the gene encoding transglutaminase X and a novel gene family member, transglutaminase Z. J. Biol. Chem..

[71] Sachslehner A.P., Surbek M., Holthaus K.B., Steinbinder J., Golabi B., Hess C., Eckhart L. (2024). The Evolution of Transglutaminases Underlies the Origin and Loss of Cornified Skin Appendages in Vertebrates. Mol. Biol. Evol..

[72] Sewa A.S., Besser H.A., Mathews I.I., Khosla C. (2024). Structural and mechanistic analysis of Ca^2+^-dependent regulation of transglutaminase 2 activity using a Ca^2+^-bound intermediate state. Proc. Natl. Acad. Sci. USA.

[73] Jeong E.M., Lee K.B., Kim G.E., Kim C.M., Lee J.H., Kim H.J., Shin J.W., Kwon M.A., Park H.H., Kim I.G. (2020). Competitive Binding of Magnesium to Calcium Binding Sites Reciprocally Regulates Transamidase and GTP Hydrolysis Activity of Transglutaminase 2. Int. J. Mol. Sci..

[74] Heggelund J.E., Das S., Stamnaes J., Iversen R., Sollid L.M. (2023). Autoantibody binding and unique enzyme-substrate intermediate conformation of human transglutaminase 3. Nat. Commun..

[75] Singh S., Hagelueken G., Ugurlar D., Urs S.U.R., Sharma A., Mahapatra M., Drepper F., Imhof D., Huesgen P.F., Oldenburg J. (2024). Cryo-EM structure of the human native plasma coagulation factor XIII complex. Blood.

[76] Murthy S.N., Iismaa S., Begg G., Freymann D.M., Graham R.M., Lorand L. (2002). Conserved tryptophan in the core domain of transglutaminase is essential for catalytic activity. Proc. Natl. Acad. Sci. USA.

[77] Iismaa S.E., Holman S., Wouters M.A., Lorand L., Graham R.M., Husain A. (2003). Evolutionary specialization of a tryptophan indole group for transition-state stabilization by eukaryotic transglutaminases. Proc. Natl. Acad. Sci. USA.

[78] Keillor J.W., Clouthier C.M., Apperley K.Y.P., Akbar A., Mulani A. (2014). Acyl transfer mechanisms of tissue transglutaminase. Bioorg. Chem..

[79] Ahvazi B., Steinert P.M. (2003). A model for the reaction mechanism of the transglutaminase 3 enzyme. Exp. Mol. Med..

[80] Hettasch J.M., Greenberg C.S. (1994). Analysis of the catalytic activity of human factor XIIIa by site-directed mutagenesis. J. Biol. Chem..

[81] Pinkas D.M., Strop P., Brunger A.T., Khosla C. (2007). Transglutaminase 2 undergoes a large conformational change upon activation. PLoS Biol..

[82] Stieler M., Weber J., Hils M., Kolb P., Heine A., Buchold C., Pasternack R., Klebe G. (2013). Structure of active coagulation factor XIII triggered by calcium binding: Basis for the design of next-generation anticoagulants. Angew. Chem. Int. Ed. Engl..

[83] Pasternack R., Hils M. (2020). Editorial for the special issue on transglutaminases in translation-Novel tools and methods impacting on diagnostics and therapeutics. Anal. Biochem..

[84] Coussons P.J., Price N.C., Kelly S.M., Smith B., Sawyer L. (1992). Factors that govern the specificity of transglutaminase-catalysed modification of proteins and peptides. Biochem. J..

[85] Gorman J.J., Folk J.E. (1984). Structural features of glutamine substrates for transglutaminases. Role of extended interactions in the specificity of human plasma factor XIIIa and of the guinea pig liver enzyme. J. Biol. Chem..

[86] Orru S., Caputo I., D’Amato A., Ruoppolo M., Esposito C. (2003). Proteomics identification of acyl-acceptor and acyl-donor substrates for transglutaminase in a human intestinal epithelial cell line. Implications for celiac disease. J. Biol. Chem..

[87] Nikolajsen C.L., Dyrlund T.F., Poulsen E.T., Enghild J.J., Scavenius C. (2014). Coagulation factor XIIIa substrates in human plasma: Identification and incorporation into the clot. J. Biol. Chem..

[88] Andre W., Nondier I., Valensi M., Guillonneau F., Federici C., Hoffner G., Djian P. (2017). Identification of brain substrates of transglutaminase by functional proteomics supports its role in neurodegenerative diseases. Neurobiol. Dis..

[89] Boroumand M., Olianas A., Manconi B., Serrao S., Iavarone F., Desiderio C., Pieroni L., Faa G., Messana I., Castagnola M. (2020). Mapping of Transglutaminase-2 Sites of Human Salivary Small Basic Proline-Rich Proteins by HPLC-High-Resolution ESI-MS/MS. J. Proteome Res..

[90] Takeuchi T., Tatsukawa H., Shinoda Y., Kuwata K., Nishiga M., Takahashi H., Hase N., Hitomi K. (2021). Spatially Resolved Identification of Transglutaminase Substrates by Proteomics in Pulmonary Fibrosis. Am. J. Respir. Cell Mol. Biol..

[91] Lexhaller B., Ludwig C., Scherf K.A. (2020). Identification of Isopeptides Between Human Tissue Transglutaminase and Wheat, Rye, and Barley Gluten Peptides. Sci. Rep..

[92] Moore P.K., Moore E.E., Garner R., Hansen K., Barrett C.D., Sauaia A., Chandler J., Janssen W.J., Moore H.B. (2026). Coagulation factor XIII: An unrecognized regulator of fibrinolytic phenotypes in trauma-A potential link to cysteine cathepsin degradation of plasminogen. J. Trauma Acute Care Surg..

[93] Hitomi K., Kitamura M., Sugimura Y. (2009). Preferred substrate sequences for transglutaminase 2: Screening using a phage-displayed peptide library. Amino Acids.

[94] Sugimura Y., Hosono M., Wada F., Yoshimura T., Maki M., Hitomi K. (2006). Screening for the preferred substrate sequence of transglutaminase using a phage-displayed peptide library: Identification of peptide substrates for TGASE 2 and Factor XIIIA. J. Biol. Chem..

[95] Sugimura Y., Hosono M., Kitamura M., Tsuda T., Yamanishi K., Maki M., Hitomi K. (2008). Identification of preferred substrate sequences for transglutaminase 1--development of a novel peptide that can efficiently detect cross-linking enzyme activity in the skin. FEBS J..

[96] Yamane A., Fukui M., Sugimura Y., Itoh M., Alea M.P., Thomas V., El Alaoui S., Akiyama M., Hitomi K. (2010). Identification of a preferred substrate peptide for transglutaminase 3 and detection of in situ activity in skin and hair follicles. FEBS J..

[97] Fukui M., Kuramoto K., Yamasaki R., Shimizu Y., Itoh M., Kawamoto T., Hitomi K. (2013). Identification of a highly reactive substrate peptide for transglutaminase 6 and its use in detecting transglutaminase activity in the skin epidermis. FEBS J..

[98] Itoh M., Kawamoto T., Tatsukawa H., Kojima S., Yamanishi K., Hitomi K. (2011). In situ detection of active transglutaminases for keratinocyte type (TGase 1) and tissue type (TGase 2) using fluorescence-labeled highly reactive substrate peptides. J. Histochem. Cytochem..

[99] Kuribayashi M., Kawaguchi Y., Teshima H., Yamaguchi H., Tatsukawa H., Hitomi K. (2021). Investigation of mouse amniotic fluid for stimulating ability of keratinocyte differentiation depending on the fetal stage. Arch. Biochem. Biophys..

[100] Sun H., Kaartinen M.T. (2020). Assessment of expression and specific activities of transglutaminases TG1, TG2, and FXIII-A during osteoclastogenesis. Anal. Biochem..

[101] Yamane M., Sugimura K., Kawasaki H., Tatsukawa H., Hitomi K. (2016). Analysis on transglutaminase 1 and its substrates using specific substrate peptide in cultured keratinocytes. Biochem. Biophys. Res. Commun..

[102] Tanabe Y., Yamane M., Kato M., Teshima H., Kuribayashi M., Tatsukawa H., Takama H., Akiyama M., Hitomi K. (2019). Studies on differentiation-dependent expression and activity of distinct transglutaminases by specific substrate peptides using three-dimensional reconstituted epidermis. FEBS J..

[103] Watanabe K., Tsunoda K., Itoh M., Fukui M., Mori H., Hitomi K. (2013). Transglutaminase 2 and Factor XIII catalyze distinct substrates in differentiating osteoblastic cell line: Utility of highly reactive substrate peptides. Amino Acids.

[104] Ito Y., Tatsukawa H., Yamaguchi H., Takahashi K., Hitomi K., Yuzawa Y. (2018). Detection and identification of potential transglutaminase 2 substrates in the mouse renal glomeruli. Arch. Biochem. Biophys..

[105] Tatsukawa H., Otsu R., Tani Y., Wakita R., Hitomi K. (2018). Isozyme-specific comprehensive characterization of transglutaminase-crosslinked substrates in kidney fibrosis. Sci. Rep..

[106] Tatsukawa H., Tani Y., Otsu R., Nakagawa H., Hitomi K. (2017). Global identification and analysis of isozyme-specific possible substrates crosslinked by transglutaminases using substrate peptides in mouse liver fibrosis. Sci. Rep..

[107] Tatsukawa H., Nakagawa H., Yee C.M., Kuwata K., Hitomi K. (2025). Transglutaminase-mediated cytokeratin modifications implicated in bile-acid-induced hepatocyte death. FEBS J..

[108] Akiyama M., Sakai K., Yanagi T., Fukushima S., Ihn H., Hitomi K., Shimizu H. (2010). Transglutaminase1 preferred substrate peptide K5 is an efficient tool in diagnosis of lamellar ichthyosis. Am. J. Pathol..

[109] Suga Y., Tsuda T., Nagai M., Sakaguchi Y., Jitsukawa O., Yamamoto M., Hitomi K., Yamanishi K. (2015). Lamellar ichthyosis with pseudoexon activation in the transglutaminase 1 gene. J. Dermatol..

[110] Tatsukawa H., Aoyama R., Hitomi K. (2023). Development of peptide-based biosensors for detecting cross-linking and deamidation activities of transglutaminases. Amino Acids.

[111] Tatsukawa H., Liu H.H., Oba S., Kamiya N., Nakanishi Y., Hitomi K. (2017). FRET-based detection of isozyme-specific activities of transglutaminases. Amino Acids.

[112] Damnjanovic J., Odake N., Fan J., Camagna M., Jia B., Kojima T., Nemoto N., Hitomi K., Nakano H. (2022). Comprehensive analysis of transglutaminase substrate preference by cDNA display coupled with next-generation sequencing and bioinformatics. Sci. Rep..

[113] Munaweera T.I.K., Damnjanovic J., Camagna M., Nezu M., Jia B., Hitomi K., Nemoto N., Nakano H. (2024). Substrate profiling of human transglutaminase 1 using cDNA display and next-generation sequencing. Biosci. Biotechnol. Biochem..

[114] Iversen R., Fleur du Pre M., Di Niro R., Sollid L.M. (2015). Igs as Substrates for Transglutaminase 2: Implications for Autoantibody Production in Celiac Disease. J. Immunol..

[115] Somodi L., Beke Debreceni I., Kis G., Cozzolino M., Kappelmayer J., Antal M., Panyi G., Bardos H., Mutch N.J., Muszbek L. (2022). Activation mechanism dependent surface exposure of cellular factor XIII on activated platelets and platelet microparticles. J. Thromb. Haemost..

[116] Anokhin B.A., Dean W.L., Smith K.A., Flick M.J., Ariens R.A.S., Philippou H., Maurer M.C. (2020). Proteolytic and nonproteolytic activation mechanisms result in conformationally and functionally different forms of coagulation factor XIII A. FEBS J..

[117] Gupta S., Biswas A., Akhter M.S., Krettler C., Reinhart C., Dodt J., Reuter A., Philippou H., Ivaskevicius V., Oldenburg J. (2016). Revisiting the mechanism of coagulation factor XIII activation and regulation from a structure/functional perspective. Sci. Rep..

[118] Anokhin B.A., Stribinskis V., Dean W.L., Maurer M.C. (2017). Activation of factor XIII is accompanied by a change in oligomerization state. FEBS J..

[119] Li B., Billur R., Maurer M.C., Kohler H.P., Raddatz Muller P., Alberio L., Schroeder V. (2018). Proline 36 of the Factor XIII Activation Peptide Plays a Crucial Role in Substrate Recognition and Zymogen Activation. Thromb. Haemost..

[120] Mohammed R.D.S., Piell K.M., Maurer M.C. (2024). Identification of Factor XIII beta-Sandwich Residues Mediating Glutamine Substrate Binding and Activation Peptide Cleavage. Thromb. Haemost..

[121] Phillips M.A., Qin Q., Mehrpouyan M., Rice R.H. (1993). Keratinocyte transglutaminase membrane anchorage: Analysis of site-directed mutants. Biochemistry.

[122] Rice R.H., Rong X.H., Chakravarty R. (1990). Proteolytic release of keratinocyte transglutaminase. Biochem. J..

[123] Ta B.M., Gallagher G.T., Chakravarty R., Rice R.H. (1990). Keratinocyte transglutaminase in human skin and oral mucosa: Cytoplasmic localization and uncoupling of differentiation markers. J. Cell Sci..

[124] Eckert R.L., Sturniolo M.T., Jans R., Kraft C.A., Jiang H., Rorke E.A. (2009). TIG3: A regulator of type I transglutaminase activity in epidermis. Amino Acids.

[125] Kim S.Y., Chung S.I., Steinert P.M. (1995). Highly active soluble processed forms of the transglutaminase 1 enzyme in epidermal keratinocytes. J. Biol. Chem..

[126] Steinert P.M., Chung S.I., Kim S.Y. (1996). Inactive zymogen and highly active proteolytically processed membrane-bound forms of the transglutaminase 1 enzyme in human epidermal keratinocytes. Biochem. Biophys. Res. Commun..

[127] Steinert P.M., Kim S.Y., Chung S.I., Marekov L.N. (1996). The transglutaminase 1 enzyme is variably acylated by myristate and palmitate during differentiation in epidermal keratinocytes. J. Biol. Chem..

[128] Egberts F., Heinrich M., Jensen J.M., Winoto-Morbach S., Pfeiffer S., Wickel M., Schunck M., Steude J., Saftig P., Proksch E. (2004). Cathepsin D is involved in the regulation of transglutaminase 1 and epidermal differentiation. J. Cell Sci..

[129] Surbek M., Van de Steene T., Sachslehner A.P., Golabi B., Griss J., Eyckerman S., Gevaert K., Eckhart L. (2023). Cornification of keratinocytes is associated with differential changes in the catalytic activity and the immunoreactivity of transglutaminase-1. Sci. Rep..

[130] Ahvazi B., Boeshans K.M., Idler W., Baxa U., Steinert P.M. (2003). Roles of calcium ions in the activation and activity of the transglutaminase 3 enzyme. J. Biol. Chem..

[131] Ahvazi B., Kim H.C., Kee S.H., Nemes Z., Steinert P.M. (2002). Three-dimensional structure of the human transglutaminase 3 enzyme: Binding of calcium ions changes structure for activation. EMBO J..

[132] Cheng T., Hitomi K., van Vlijmen-Willems I.M., de Jongh G.J., Yamamoto K., Nishi K., Watts C., Reinheckel T., Schalkwijk J., Zeeuwen P.L. (2006). Cystatin M/E is a high affinity inhibitor of cathepsin V and cathepsin L by a reactive site that is distinct from the legumain-binding site. A novel clue for the role of cystatin M/E in epidermal cornification. J. Biol. Chem..

[133] Pietroni V., Di Giorgi S., Paradisi A., Ahvazi B., Candi E., Melino G. (2008). Inactive and highly active, proteolytically processed transglutaminase-5 in epithelial cells. J. Investig. Dermatol..

[134] Hitomi K., Kojima S., Fesus L. (2015). Transglutaminases. Multiple Functional Modifiers and Targets for New Drug Discovery.

[135] Singh S., Dodt J., Volkers P., Hethershaw E., Philippou H., Ivaskevicius V., Imhof D., Oldenburg J., Biswas A. (2019). Structure functional insights into calcium binding during the activation of coagulation factor XIII A. Sci. Rep..

[136] Jumper J., Evans R., Pritzel A., Green T., Figurnov M., Ronneberger O., Tunyasuvunakool K., Bates R., Zidek A., Potapenko A. (2021). Highly accurate protein structure prediction with AlphaFold. Nature.

[137] Varadi M., Anyango S., Deshpande M., Nair S., Natassia C., Yordanova G., Yuan D., Stroe O., Wood G., Laydon A. (2022). AlphaFold Protein Structure Database: Massively expanding the structural coverage of protein-sequence space with high-accuracy models. Nucleic Acids Res..

[138] Han B.G., Cho J.W., Cho Y.D., Jeong K.C., Kim S.Y., Lee B.I. (2010). Crystal structure of human transglutaminase 2 in complex with adenosine triphosphate. Int. J. Biol. Macromol..

[139] Jang T.H., Lee D.S., Choi K., Jeong E.M., Kim I.G., Kim Y.W., Chun J.N., Jeon J.H., Park H.H. (2014). Crystal structure of transglutaminase 2 with GTP complex and amino acid sequence evidence of evolution of GTP binding site. PLoS ONE.

[140] Iversen R., Heggelund J.E., Das S., Hoydahl L.S., Sollid L.M. (2025). Enzyme-activating B-cell receptors boost antigen presentation to pathogenic T cells in gluten-sensitive autoimmunity. Nat. Commun..

[141] Yee V.C., Pedersen L.C., Le Trong I., Bishop P.D., Stenkamp R.E., Teller D.C. (1994). Three-dimensional structure of a transglutaminase: Human blood coagulation factor XIII. Proc. Natl. Acad. Sci. USA.

[142] Yee V.C., Pedersen L.C., Bishop P.D., Stenkamp R.E., Teller D.C. (1995). Structural evidence that the activation peptide is not released upon thrombin cleavage of factor XIII. Thromb. Res..

[143] Weiss M.S., Metzner H.J., Hilgenfeld R. (1998). Two non-proline cis peptide bonds may be important for factor XIII function. FEBS Lett..

[144] Fox B.A., Yee V.C., Pedersen L.C., Le Trong I., Bishop P.D., Stenkamp R.E., Teller D.C. (1999). Identification of the calcium binding site and a novel ytterbium site in blood coagulation factor XIII by x-ray crystallography. J. Biol. Chem..

[145] Sadasivan C., Yee V.C. (2000). Interaction of the factor XIII activation peptide with alpha -thrombin. Crystal structure of its enzyme-substrate analog complex. J. Biol. Chem..

[146] Wieser H. (2007). Chemistry of gluten proteins. Food Microbiol..

[147] Taraghikhah N., Ashtari S., Asri N., Shahbazkhani B., Al-Dulaimi D., Rostami-Nejad M., Rezaei-Tavirani M., Razzaghi M.R., Zali M.R. (2020). An updated overview of spectrum of gluten-related disorders: Clinical and diagnostic aspects. BMC Gastroenterol..

[148] Karpati S., Sardy M., Nemeth K., Mayer B., Smyth N., Paulsson M., Traupe H. (2018). Transglutaminases in autoimmune and inherited skin diseases: The phenomena of epitope spreading and functional compensation. Exp. Dermatol..

[149] Besser H.A., Khosla C. (2023). Celiac disease: Mechanisms and emerging therapeutics. Trends Pharmacol. Sci..

[150] Catassi C., Verdu E.F., Bai J.C., Lionetti E. (2022). Coeliac disease. Lancet.

[151] Singh P., Arora A., Strand T.A., Leffler D.A., Catassi C., Green P.H., Kelly C.P., Ahuja V., Makharia G.K. (2018). Global Prevalence of Celiac Disease: Systematic Review and Meta-analysis. Clin. Gastroenterol. Hepatol..

[152] Rubio-Tapia A., Hill I.D., Semrad C., Kelly C.P., Greer K.B., Limketkai B.N., Lebwohl B. (2023). American College of Gastroenterology Guidelines Update: Diagnosis and Management of Celiac Disease. Am. J. Gastroenterol..

[153] Korponay-Szabo I.R., Dahlbom I., Laurila K., Koskinen S., Woolley N., Partanen J., Kovacs J.B., Maki M., Hansson T. (2003). Elevation of IgG antibodies against tissue transglutaminase as a diagnostic tool for coeliac disease in selective IgA deficiency. Gut.

[154] Fontana G., Ziberna F., Barbi E., Di Leo G., De Leo L. (2025). Intestinal celiac disease-related autoantibodies. Front. Immunol..

[155] Mandile R., Maglio M., Mosca C., Marano A., Discepolo V., Troncone R., Auricchio R. (2022). Mucosal Healing in Celiac Disease: Villous Architecture and Immunohistochemical Features in Children on a Long-Term Gluten Free Diet. Nutrients.

[156] Nestares T., Martin-Masot R. (2025). Advances in the Prevention and Management of Celiac Disease. Nutrients.

[157] Pumar M., Choo S., Rosenbaum J., Alex G., Ho S.S.C. (2025). No-Biopsy Diagnosis of Coeliac Disease in Children Without Anti-Endomysial IgA Antibody Testing: Combining Anti-Tissue Transglutaminase IgA and Anti-Deamidated Gliadin IgG Antibodies. J. Paediatr. Child. Health.

[158] Makharia G.K., Singh P., Catassi C., Sanders D.S., Leffler D., Ali R.A.R., Bai J.C. (2022). The global burden of coeliac disease: Opportunities and challenges. Nat. Rev. Gastroenterol. Hepatol..

[159] Patel V., Joharapurkar A., Jain M. (2025). Recent Development in Celiac Disease: Pathophysiology, Animal Models and Treatments. Drug Dev. Res..

[160] Girbal-Gonzalez M., Perez-Cano F.J. (2025). Is There a Future Without Gluten Restrictions for Celiac Patients? Update on Current Treatments. Nutrients.

[161] Schuppan D., Maki M., Lundin K.E.A., Isola J., Friesing-Sosnik T., Taavela J., Popp A., Koskenpato J., Langhorst J., Hovde O. (2021). A Randomized Trial of a Transglutaminase 2 Inhibitor for Celiac Disease. N. Engl. J. Med..

[162] Isola J., Maki M., Hils M., Pasternack R., Viiri K., Dotsenko V., Montonen T., Zimmermann T., Mohrbacher R., Greinwald R. (2023). The Oral Transglutaminase 2 Inhibitor ZED1227 Accumulates in the Villous Enterocytes in Celiac Disease Patients during Gluten Challenge and Drug Treatment. Int. J. Mol. Sci..

[163] Dotsenko V., Tewes B., Hils M., Pasternack R., Isola J., Taavela J., Popp A., Sarin J., Huhtala H., Hiltunen P. (2024). Transcriptomic analysis of intestine following administration of a transglutaminase 2 inhibitor to prevent gluten-induced intestinal damage in celiac disease. Nat. Immunol..

[164] Ryan B., Tribe S., Keillor J.W. (2026). Tissue transglutaminase inhibitors over the past decade. Bioorg. Med. Chem..

[165] Kelly C.P., Murray J.A., Leffler D.A., Getts D.R., Bledsoe A.C., Smithson G., First M.R., Morris A., Boyne M., Elhofy A. (2021). TAK-101 Nanoparticles Induce Gluten-Specific Tolerance in Celiac Disease: A Randomized, Double-Blind, Placebo-Controlled Study. Gastroenterology.

[166] Hoilat G.J., Altowairqi A.K., Ayas M.F., Alhaddab N.T., Alnujaidi R.A., Alharbi H.A., Alyahyawi N., Kamal A., Alhabeeb H., Albazee E. (2022). Larazotide acetate for treatment of celiac disease: A systematic review and meta-analysis of randomized controlled trials. Clin. Res. Hepatol. Gastroenterol..

[167] Cellier C., Bouma G., van Gils T., Khater S., Malamut G., Crespo L., Collin P., Green P.H.R., Crowe S.E., Tsuji W. (2019). Safety and efficacy of AMG 714 in patients with type 2 refractory coeliac disease: A phase 2a, randomised, double-blind, placebo-controlled, parallel-group study. Lancet Gastroenterol. Hepatol..

[168] Lahdeaho M.L., Scheinin M., Vuotikka P., Taavela J., Popp A., Laukkarinen J., Koffert J., Koivurova O.P., Pesu M., Kivela L. (2019). Safety and efficacy of AMG 714 in adults with coeliac disease exposed to gluten challenge: A phase 2a, randomised, double-blind, placebo-controlled study. Lancet Gastroenterol. Hepatol..

[169] Das S., Stamnaes J., Kemppainen E., Hervonen K., Lundin K.E.A., Parmar N., Jahnsen F.L., Jahnsen J., Lindfors K., Salmi T. (2023). Correction to: Separate Gut Plasma Cell Populations Produce Auto-Antibodies against Transglutaminase 2 and Transglutaminase 3 in Dermatitis Herpetiformis. Adv. Sci..

[170] Rybak-d’Obyrn J., Placek W. (2022). Etiopathogenesis of dermatitis herpetiformis. Postepy Dermatol. Alergol..

[171] Didona D., Maglie R., Solimani F. (2025). Gluten-related skin disorders: Clinical presentation, diagnostic and treatments. J. Dtsch. Dermatol. Ges..

[172] Nguyen C.N., Kim S.J. (2021). Dermatitis Herpetiformis: An Update on Diagnosis, Disease Monitoring, and Management. Medicina.

[173] Dmochowski M., Gornowicz-Porowska J., Bowszyc-Dmochowska M. (2019). An update on direct immunofluorescence for diagnosing dermatitis herpetiformis. Postepy Dermatol. Alergol..

[174] Boch K., Heck F., Hammers C.M., Antiga E., Caproni M., Juhl D., Goletz S., Horvath O.N., Huilaja L., Khil’chenko S. (2023). Serum reactivity in dermatitis herpetiformis: An international multicentre study. Clin. Exp. Dermatol..

[175] Hadjivassiliou M., Aeschlimann P., Sanders D.S., Maki M., Kaukinen K., Grunewald R.A., Bandmann O., Woodroofe N., Haddock G., Aeschlimann D.P. (2013). Transglutaminase 6 antibodies in the diagnosis of gluten ataxia. Neurology.

[176] Hadjivassiliou M., Croall I.D., Zis P., Sarrigiannis P.G., Sanders D.S., Aeschlimann P., Grunewald R.A., Armitage P.A., Connolly D., Aeschlimann D. (2019). Neurologic Deficits in Patients With Newly Diagnosed Celiac Disease Are Frequent and Linked With Autoimmunity to Transglutaminase 6. Clin. Gastroenterol. Hepatol..

[177] Boscolo S., Lorenzon A., Sblattero D., Florian F., Stebel M., Marzari R., Not T., Aeschlimann D., Ventura A., Hadjivassiliou M. (2010). Anti transglutaminase antibodies cause ataxia in mice. PLoS ONE.

[178] Stamnaes J., Dorum S., Fleckenstein B., Aeschlimann D., Sollid L.M. (2010). Gluten T cell epitope targeting by TG3 and TG6; implications for dermatitis herpetiformis and gluten ataxia. Amino Acids.

[179] Floare M.L., Wharton S.B., Simpson J.E., Aeschlimann D., Hadjivassiliou M. (2025). CSF Markers of TG6 Autoimmunity in Gluten Ataxia. Cerebellum.

[180] Floare M.L., Wharton S.B., Simpson J.E., Aeschlimann D., Hoggard N., Hadjivassiliou M. (2024). Cerebellar degeneration in gluten ataxia is linked to microglial activation. Brain Commun..

[181] Hadjivassiliou M., Grunewald R.A., Sanders D.S., Shanmugarajah P., Hoggard N. (2017). Effect of gluten-free diet on cerebellar MR spectroscopy in gluten ataxia. Neurology.

[182] Wilkinson I.D., Hadjivassiliou M., Dickson J.M., Wallis L., Grunewald R.A., Coley S.C., Widjaja E., Griffiths P.D. (2005). Cerebellar abnormalities on proton MR spectroscopy in gluten ataxia. J. Neurol. Neurosurg. Psychiatry.

[183] Croall I.D., Hadjivassiliou M., Sanders D.S., Teh K., Biancardi A.M., Trott N., Hoggard N. (2025). Persisting Transglutaminase 6 Antibodies in Neurological Gluten-Related Disorders. Ann. Neurol..

[184] Zis P., Rao D.G., Sarrigiannis P.G., Aeschlimann P., Aeschlimann D.P., Sanders D., Grunewald R.A., Hadjivassiliou M. (2017). Transglutaminase 6 antibodies in gluten neuropathy. Dig. Liver Dis..

[185] Rouvroye M.D., Warendorf J., Vrancken A., Eftimov F., Wieske L., Faber C.G., Hoeijmakers J.G.J., Damoiseaux J., van de Warrenburg B., van Gaalen J. (2025). Serological analysis of gluten-related antibodies in idiopathic neuropathies and cerebellar ataxia. J. Neurol..

[186] Hadjivassiliou M., Reunala T., Hervonen K., Aeschlimann P., Aeschlimann D. (2020). TG6 Auto-Antibodies in Dermatitis Herpetiformis. Nutrients.

[187] Croall I.D., Armitage P.A., Hadjivassiliou M., Sanders D.S., Hoggard N. (2025). Anti-gliadin Antibodies and the Brain in People Without Celiac Disease: A Case-Control Study. Am. J. Gastroenterol..

[188] Zis P., Hadjivassiliou M. (2019). Treatment of Neurological Manifestations of Gluten Sensitivity and Coeliac Disease. Curr. Treat. Options Neurol..

[189] Duranteau O., Tatar G., Demulder A., Tuna T. (2023). Acquired factor XIII deficiency: A scoping review. Eur. J. Anaesthesiol. Intensive Care.

[190] Souri M., Osaki T., Ichinose A. (2024). Detection of factor XIII inhibitors in 33 patients with autoimmune factor XIII deficiency in Japan. Int. J. Hematol..

[191] Iismaa S.E. (2016). The prostate-specific protein, transglutaminase 4 (TG4), is an autoantigen associated with male subfertility. Ann. Transl. Med..

[192] Ferre E.M.N., Schmitt M.M., Lionakis M.S. (2021). Autoimmune Polyendocrinopathy-Candidiasis-Ectodermal Dystrophy. Front. Pediatr..

[193] Besnard M., Padonou F., Provin N., Giraud M., Guillonneau C. (2021). AIRE deficiency, from preclinical models to human APECED disease. Dis. Model. Mech..

[194] Elfstrom P., Sundstrom J., Ludvigsson J.F. (2014). Systematic review with meta-analysis: Associations between coeliac disease and type 1 diabetes. Aliment. Pharmacol. Ther..

[195] Karimzadhagh S., Abbaspour E., Shahriarinamin M., Shamsi P., Poursadrolah S., Khorasani M., Daghighi M., Malek A., Talesh J.T., Makharia G.K. (2025). Meta-Analysis: Global Prevalence of Coeliac Disease in Type 1 Diabetes. Aliment. Pharmacol. Ther..

[196] Roy A., Laszkowska M., Sundstrom J., Lebwohl B., Green P.H., Kampe O., Ludvigsson J.F. (2016). Prevalence of Celiac Disease in Patients with Autoimmune Thyroid Disease: A Meta-Analysis. Thyroid.

[197] Bartoloni E., Bistoni O., Alunno A., Cavagna L., Nalotto L., Baldini C., Priori R., Fischetti C., Fredi M., Quartuccio L. (2019). Celiac Disease Prevalence is Increased in Primary Sjogren’s Syndrome and Diffuse Systemic Sclerosis: Lessons from a Large Multi-Center Study. J. Clin. Med..

[198] Abbad L., Monteiro R.C., Berthelot L. (2020). Food antigens and Transglutaminase 2 in IgA nephropathy: Molecular links between gut and kidney. Mol. Immunol..

[199] Dutta R., Rawat R., Das P., Singh G., Kumari A., Ahmad M., Chauhan A., Ahuja V., Agrawal S.K., Makharia G.K. (2023). Identification of celiac disease associated IgA nephropathy by IgA anti-tissue transglutaminase2 antibody deposits in archived formalin-fixed tissues. Saudi J. Gastroenterol..

[200] Nurmi R., Korponay-Szabo I., Laurila K., Huhtala H., Niemela O., Mustonen J., Makela S., Kaukinen K., Lindfors K. (2021). Celiac Disease-Type Tissue Transglutaminase Autoantibody Deposits in Kidney Biopsies of Patients with IgA Nephropathy. Nutrients.

[201] Emami M.H., Najafi M.R., Allahdadian S., Mohammadzadeh S., Jamali N., Lalazarian A., Shaygan Nejad V., Maghool F. (2024). Evaluation of the Prevalence of Anti-transglutaminase 2 and 6 Antibodies in Patients with Sero-Positive Multiple Sclerosis. Middle East. J. Dig. Dis..

[202] Visser A.E., Pazoki R., Pulit S.L., van Rheenen W., Raaphorst J., van der Kooi A.J., Ricano-Ponce I., Wijmenga C., Otten H.G., Veldink J.H. (2017). No association between gluten sensitivity and amyotrophic lateral sclerosis. J. Neurol..

[203] Sun Q., Burgren N.M., Cheraghlou S., Paller A.S., Larralde M., Bercovitch L., Levinsohn J., Ren I., Hu R.H., Zhou J. (2022). The Genomic and Phenotypic Landscape of Ichthyosis: An Analysis of 1000 Kindreds. JAMA Dermatol..

[204] Diociaiuti A., Corbeddu M., Rossi S., Pisaneschi E., Cesario C., Condorelli A.G., Samela T., Giancristoforo S., Angioni A., Zambruno G. (2024). Cross-Sectional Study on Autosomal Recessive Congenital Ichthyoses: Association of Genotype with Disease Severity, Phenotypic, and Ultrastructural Features in 74 Italian Patients. Dermatology.

[205] Richard G., Adam M.P., Feldman J., Mirzaa G.M., Pagon R.A., Wallace S.E., Amemiya A. (1993). Autosomal Recessive Congenital Ichthyosis. GeneReviews^®^.

[206] Paller A.S., Akiyama M., Hernandez-Martin A., Mazereeuw-Hautier J., Sprecher E., Reclassifying Epidermal Differentiation Disorders I. (2026). New gene-based classification of ichthyoses and palmoplantar keratodermas: Hereditary epidermal differentiation disorders. J. Am. Acad. Dermatol..

[207] Akiyama M., Choate K., Hernandez-Martin A., Aldwin-Easton M., Bodemer C., Gostynski A., Hovnanian A., Ishida-Yamamoto A., Malovitski K., O’Toole E.A. (2025). Nonsyndromic epidermal differentiation disorders: A new classification toward pathogenesis-based therapy. Br. J. Dermatol..

[208] El Hachem M., De Marco R., Soria de Francisco J.M., Audouze A., Aldwin-Easton M., Skayem C., Taieb C., Saint Aroman M., Ghienne H., Baissac C. (2024). Ichthyosis: Multinational European study on patient characteristics, involved body sites and impact on quality of life. Br. J. Dermatol..

[209] Gulnerman E.K., Hanedan N., Akillioglu M., Kayhan G., Adisen E., Erdem O., Hirfanoglu I.M., Ergenekon E., Onal E.E., Turkyilmaz C. (2023). Novel Compound Heterozygous Mutations of TGM1 Gene Identified in a Turkish Collodion Baby Diagnosed with Non-Bullous Congenital Ichthyosiform Erythroderma. Ann. Dermatol..

[210] Oji V., Hautier J.M., Ahvazi B., Hausser I., Aufenvenne K., Walker T., Seller N., Steijlen P.M., Kuster W., Hovnanian A. (2006). Bathing suit ichthyosis is caused by transglutaminase-1 deficiency: Evidence for a temperature-sensitive phenotype. Hum. Mol. Genet..

[211] Zaenglein A.L., Levy M.L., Stefanko N.S., Benjamin L.T., Bruckner A.L., Choate K., Craiglow B.G., DiGiovanna J.J., Eichenfield L.F., Elias P. (2022). Executive summary: Consensus recommendations for the use of retinoids in ichthyosis and other disorders of cornification in children and adolescents. J. Am. Acad. Dermatol..

[212] Aufenvenne K., Larcher F., Hausser I., Duarte B., Oji V., Nikolenko H., Del Rio M., Dathe M., Traupe H. (2013). Topical enzyme-replacement therapy restores transglutaminase 1 activity and corrects architecture of transglutaminase-1-deficient skin grafts. Am. J. Hum. Genet..

[213] Freedman J.C., Parry T.J., Zhang P., Majumdar A., Krishnan S., Regula L.K., O’Malley M., Coghlan S., Yogesha S.D., Ramasamy S. (2021). Preclinical Evaluation of a Modified Herpes Simplex Virus Type 1 Vector Encoding Human TGM1 for the Treatment of Autosomal Recessive Congenital Ichthyosis. J. Investig. Dermatol..

[214] Sercia L., Romano O., Marini G., Enzo E., Forcato M., De Rosa L., De Luca M. (2024). A cellular disease model toward gene therapy of TGM1-dependent lamellar ichthyosis. Mol. Ther. Methods Clin. Dev..

[215] Chulpanova D.S., Shaimardanova A.A., Ponomarev A.S., Elsheikh S., Rizvanov A.A., Solovyeva V.V. (2022). Current Strategies for the Gene Therapy of Autosomal Recessive Congenital Ichthyosis and Other Types of Inherited Ichthyosis. Int. J. Mol. Sci..

[216] FB U.B., Cau L., Tafazzoli A., Mechin M.C., Wolf S., Romano M.T., Valentin F., Wiegmann H., Huchenq A., Kandil R. (2016). Mutations in Three Genes Encoding Proteins Involved in Hair Shaft Formation Cause Uncombable Hair Syndrome. Am. J. Hum. Genet..

[217] Roberson J.L., Farzaneh C., Neylan C.J., Judy R., Walker V., Damrauer S.M., Levin M.G., Maguire L.H., Regeneron Genetics C., Penn Medicine B. (2024). Genome-Wide Association Study Identifies Genes for Hair Growth and Patterning are Associated With Pilonidal Disease. Dis. Colon Rectum.

[218] Basmanav F.B., Cesarato N., Kumar S., Borisov O., Kokordelis P., Ralser D.J., Wehner M., Axt D., Xiong X., Thiele H. (2022). Assessment of the Genetic Spectrum of Uncombable Hair Syndrome in a Cohort of 107 Individuals. JAMA Dermatol..

[219] Lorand L., Iismaa S.E. (2019). Transglutaminase diseases: From biochemistry to the bedside. FASEB J..

[220] John S., Thiebach L., Frie C., Mokkapati S., Bechtel M., Nischt R., Rosser-Davies S., Paulsson M., Smyth N. (2012). Epidermal transglutaminase (TGase 3) is required for proper hair development, but not the formation of the epidermal barrier. PLoS ONE.

[221] van der Velden J.J., van Geel M., Nellen R.G., Jonkman M.F., McGrath J.A., Nanda A., Sprecher E., van Steensel M.A., McLean W.H., Cassidy A.J. (2015). Novel TGM5 mutations in acral peeling skin syndrome. Exp. Dermatol..

[222] Pampalakis G., Kiritsi D., Zingkou E., Franzke C.W., Valari M., Sotiropoulou G. (2017). Enhanced Proteolytic Activities in Acral Peeling Skin Syndrome: A Role of Transglutaminase 5 in Epidermal Homeostasis. J. Invest. Dermatol..

[223] Hitomi K. (2005). Transglutaminases in skin epidermis. Eur. J. Dermatol..

[224] Chen Y., Geng J., Xiao Y., Zhou X., Li M., Li W. (2025). A case of peeling skin syndrome type 1 with novel CDSN gene variation successfully treated with upadacitinib. J. Dermatol..

[225] Sarika G.M., Ibrahim R., Zlotogorski A., Molho-Pessach V. (2021). Acral peeling skin syndrome resulting from a novel homozygous mutation in the CSTA gene-A report of two cases. Pediatr. Dermatol..

[226] Canueto J., Bueno E., Rodriguez-Diaz E., Vicente-Diaz M.A., Alvarez-Cuesta C.C., Gonzalvo-Rodriguez P., Gonzalez-Sarmiento R. (2016). Acral peeling skin syndrome resulting from mutations in TGM5. J. Eur. Acad. Dermatol. Venereol..

[227] Pigors M., Kiritsi D., Cobzaru C., Schwieger-Briel A., Suarez J., Faletra F., Aho H., Makela L., Kern J.S., Bruckner-Tuderman L. (2012). TGM5 mutations impact epidermal differentiation in acral peeling skin syndrome. J. Investig. Dermatol..

[228] Szczecinska W., Nesteruk D., Wertheim-Tysarowska K., Greenblatt D.T., Baty D., Browne F., Liu L., Ozoemena L., Terron-Kwiatkowski A., McGrath J.A. (2014). Under-recognition of acral peeling skin syndrome: 59 new cases with 15 novel mutations. Br. J. Dermatol..

[229] Sticova E., Kveton M., Dubska M., Kubatova A. (2019). Acral peeling skin syndrome: An underdiagnosed skin disorder. Indian J. Dermatol. Venereol. Leprol..

[230] Stjernbrandt A.L., Burstedt M., Holmbom E., Shayesteh A. (2024). Acral Peeling Skin Syndrome: Two Unusual Cases and the Therapeutic Potential of Botulinum Toxin. Acta Derm. Venereol..

[231] Maass F., Jamous A., Biskup S., Eisenberg H., D’Hedouville Z., Bahr M., van Riesen C. (2023). Spinocerebellar Ataxia Type 35 Caused by a New TGM6 Variant: Video Documentation of a German Family. Mov. Disord. Clin. Pract..

[232] Guo Y.C., Lin J.J., Liao Y.C., Tsai P.C., Lee Y.C., Soong B.W. (2014). Spinocerebellar ataxia 35: Novel mutations in TGM6 with clinical and genetic characterization. Neurology.

[233] Manini A., Bocci T., Migazzi A., Monfrini E., Ronchi D., Franco G., De Rosa A., Sartucci F., Priori A., Corti S. (2020). A case report of late-onset cerebellar ataxia associated with a rare p.R342W TGM6 (SCA35) mutation. BMC Neurol..

[234] Sharawat I.K., Panda P.K., Bhunia N.S., Dawman L. (2021). Clinical Spectrum of TGM6-Related Movement Disorders: A New Report with a Pooled Analysis of 48 Patients. J. Neurosci. Rural Pract..

[235] Guan W.J., Wang J.L., Liu Y.T., Ma Y.T., Zhou Y., Jiang H., Shen L., Guo J.F., Xia K., Li J.D. (2013). Spinocerebellar ataxia type 35 (SCA35)-associated transglutaminase 6 mutants sensitize cells to apoptosis. Biochem. Biophys. Res. Commun..

[236] Cheng H.L., Dong H.L., Liu D.S., Ni W., Ma Y., Yang L., Du Y.C., Chen D.F., Dong Y., Wu Z.Y. (2021). TGM6 might not be a specific causative gene for spinocerebellar ataxia resulting from genetic analysis and functional study. Gene.

[237] Dorgalaleh A., Tabibian S., Hosseini M.S., Farshi Y., Roshanzamir F., Naderi M., Kazemi A., Zaker F., Aghideh A.N., Shamsizadeh M. (2016). Diagnosis of factor XIII deficiency. Hematology.

[238] Ito Y., Tsuji S., Kasahara M., Tokoro S., Murakami T., Takayama H. (2024). Successful perinatal management of a woman with congenital factor XIII deficiency using recombinant factor XIII: A case report and literature review. J. Obstet. Gynaecol. Res..

[239] Ejaz M., Saleem A., Ali N., Tariq F. (2019). Factor XIII deficiency with intracranial haemorrhage. BMJ Case Rep..

[240] Souri M., Yee V.C., Fujii N., Ichinose A. (2012). Molecular modeling predicts structural changes in the A subunit of factor XIII caused by two novel mutations identified in a neonate with severe congenital factor XIII deficiency. Thromb. Res..

[241] Al Sharif M.A., Mathews N., Tasneem S., Moffat K.A., Carlino S.A., Mithoowani S., Hayward C.P.M. (2025). Measurement of factor XIII for the diagnosis and management of deficiencies: Insights from a retrospective review of 10 years of data on consecutive samples and patients. Res. Pract. Thromb. Haemost..

[242] Louhichi N., Haj Salem I., Medhaffar M., Miled N., Hadji A.F., Keskes L., Fakhfakh F. (2017). Original tandem duplication in FXIIIA gene with splicing site modification and four amino acids insertion causes factor XIII deficiency. Blood Coagul. Fibrinolysis.

[243] Poulsen L.H., Kerlin B.A., Castaman G., Molinari A.C., Menegatti M., Nugent D., Dey S., Garly M.L., Carcao M. (2022). Safety and effectiveness of recombinant factor XIII-A(2) in congenital factor XIII deficiency: Real-world evidence. Res. Pract. Thromb. Haemost..

[244] Pasca S., PierGiorgio C., Pea F., Ezio Z., The Italian rFXIII Study Group (2022). The pharmacokinetics of recombinant FXIII (catridecacog) from the MENTOR(TM)2 trial to a real-world study: A head-to-head comparison. J. Thromb. Thrombolysis.

[245] Zanon E., Pasca S., Sottilotta G., Molinari A.C., Ferretti A., Di Gregorio P., Pollio B., Pizzuti M., Notarangelo L.D., Biasoli C. (2023). A multicenter, real-world experience with recombinant FXIII for the treatment of patients with FXIII deficiency: From pharmacokinetics to clinical practice. The Italian FXIII Study. Blood Transfus..

[246] Dorgalaleh A., Kiani J., Zaker F., Safa M. (2022). The most common disease-causing mutation of factor XIII deficiency is corrected by CRISPR/CAS9 gene editing system. Blood Coagul. Fibrinolysis.

[247] Panes-Fernandez J., Marileo A.M., Espinoza-Rubilar N., Meza M.E., Salgado-Martinez B.A., Gaete-Riquelme K., Moraga-Cid G., Castro P.A., Burgos C.F., Fuentealba J. (2025). The Alkaloid Gelsemine Reduces Abeta Peptide Toxicity by Targeting Transglutaminase Type 2 Enzyme. Plants.

[248] Wilhelmus M.M.M., Tonoli E., Coveney C., Boocock D.J., Jongenelen C.A.M., Breve J.J.P., Verderio E.A.M., Drukarch B. (2022). The Transglutaminase-2 Interactome in the APP23 Mouse Model of Alzheimer’s Disease. Cells.

[249] Zhang J., Grosso Jasutkar H., Yan R., Woo J.M., Lee K.W., Im J.Y., Junn E., Iismaa S.E., Mouradian M.M. (2020). Transglutaminase 2 Depletion Attenuates alpha-Synuclein Mediated Toxicity in Mice. Neuroscience.

[250] Mastroberardino P.G., Piacentini M. (2010). Type 2 transglutaminase in Huntington’s disease: A double-edged sword with clinical potential. J. Intern. Med..

[251] Menalled L.B., Kudwa A.E., Oakeshott S., Farrar A., Paterson N., Filippov I., Miller S., Kwan M., Olsen M., Beltran J. (2014). Genetic deletion of transglutaminase 2 does not rescue the phenotypic deficits observed in R6/2 and zQ175 mouse models of Huntington’s disease. PLoS ONE.

[252] Kumar A., Kneynsberg A., Tucholski J., Perry G., van Groen T., Detloff P.J., Lesort M. (2012). Tissue transglutaminase overexpression does not modify the disease phenotype of the R6/2 mouse model of Huntington’s disease. Exp. Neurol..

[253] Wilhelmus M.M., Grunberg S.C., Bol J.G., van Dam A.M., Hoozemans J.J., Rozemuller A.J., Drukarch B. (2009). Transglutaminases and transglutaminase-catalyzed cross-links colocalize with the pathological lesions in Alzheimer’s disease brain. Brain Pathol..

[254] Schulze-Krebs A., Canneva F., Stemick J., Plank A.C., Harrer J., Bates G.P., Aeschlimann D., Steffan J.S., von Horsten S. (2021). Transglutaminase 6 Is Colocalized and Interacts with Mutant Huntingtin in Huntington Disease Rodent Animal Models. Int. J. Mol. Sci..

[255] Keillor J.W., Johnson G.V.W. (2021). Transglutaminase 2 as a therapeutic target for neurological conditions. Expert. Opin. Ther. Targets.

[256] Chen S., Ma J., Chi J., Zhang B., Zheng X., Chen J., Liu J. (2022). Roles and potential clinical implications of tissue transglutaminase in cardiovascular diseases. Pharmacol. Res..

[257] Selcuk K., Leitner A., Braun L., Le Blanc F., Pacak P., Pot S., Vogel V. (2024). Transglutaminase 2 has higher affinity for relaxed than for stretched fibronectin fibers. Matrix Biol..

[258] Wang H., Chen J., Jandu S., Melucci S., Savage W., Nandakumar K., Kang S.K., Barreto-Ortiz S., Poe A., Rastogi S. (2021). Probing tissue transglutaminase mediated vascular smooth muscle cell aging using a novel transamidation-deficient Tgm2-C277S mouse model. Cell Death Discov..

[259] Jandu S.K., Webb A.K., Pak A., Sevinc B., Nyhan D., Belkin A.M., Flavahan N.A., Berkowitz D.E., Santhanam L. (2013). Nitric oxide regulates tissue transglutaminase localization and function in the vasculature. Amino Acids.

[260] Steppan J., Sikka G., Jandu S., Barodka V., Halushka M.K., Flavahan N.A., Belkin A.M., Nyhan D., Butlin M., Avolio A. (2014). Exercise, vascular stiffness, and tissue transglutaminase. J. Am. Heart Assoc..

[261] Shinde A.V., Su Y., Palanski B.A., Fujikura K., Garcia M.J., Frangogiannis N.G. (2018). Pharmacologic inhibition of the enzymatic effects of tissue transglutaminase reduces cardiac fibrosis and attenuates cardiomyocyte hypertrophy following pressure overload. J. Mol. Cell Cardiol..

[262] Pinilla E., Comerma-Steffensen S., Prat-Duran J., Rivera L., Matchkov V.V., Buus N.H., Simonsen U. (2021). Transglutaminase 2 Inhibitor LDN 27219 Age-Dependently Lowers Blood Pressure and Improves Endothelium-Dependent Vasodilation in Resistance Arteries. Hypertension.

[263] Soltani F., Kaartinen M.T. (2023). Transglutaminases in fibrosis-overview and recent advances. Am. J. Physiol. Cell Physiol..

[264] Nahrendorf M., Hu K., Frantz S., Jaffer F.A., Tung C.H., Hiller K.H., Voll S., Nordbeck P., Sosnovik D., Gattenlohner S. (2006). Factor XIII deficiency causes cardiac rupture, impairs wound healing, and aggravates cardiac remodeling in mice with myocardial infarction. Circulation.

[265] Nahrendorf M., Aikawa E., Figueiredo J.L., Stangenberg L., van den Borne S.W., Blankesteijn W.M., Sosnovik D.E., Jaffer F.A., Tung C.H., Weissleder R. (2008). Transglutaminase activity in acute infarcts predicts healing outcome and left ventricular remodelling: Implications for FXIII therapy and antithrombin use in myocardial infarction. Eur. Heart J..

[266] Gemmati D., Zeri G., Orioli E., Mari R., Moratelli S., Vigliano M., Marchesini J., Grossi M.E., Pecoraro A., Cuneo A. (2015). Factor XIII-A dynamics in acute myocardial infarction: A novel prognostic biomarker?. Thromb. Haemost..

[267] Traub J., Abu Hussein M., Schmitt D., Frey A. (2025). Linking factor XIII activity to all-cause mortality after myocardial infarction: The overlooked role of serum albumin. Int. J. Cardiol. Heart Vasc..

[268] Wang M., Xu Y., Meng Y., Xie W., Chen J., Du J. (2025). Stiffness-activated hepatic stellate cells boost HCC migration via TGM2/ITGB1-mediated matrix remodeling and mitochondrial transfer. JHEP Rep..

[269] Tatsukawa H., Takeuchi T., Shinoda Y., Hitomi K. (2020). Identification and characterization of substrates crosslinked by transglutaminases in liver and kidney fibrosis. Anal. Biochem..

[270] Dik K., de Bruijne J., Takkenberg R.B., Roelofs J.J., Tempelmans M.J., Dijkgraaf M.G., Gelderblom H.C., Reesink H.W., Meijers J.C., Jansen P.L. (2012). Factor XIII Val34Leu mutation accelerates the development of fibrosis in patients with chronic hepatitis B and C. Hepatol. Res..

[271] Toida M., Okumura Y., Takami T. (1991). Cells containing factor XIIIa and pulmonary fibrosis induced by bleomycin. J. Clin. Pathol..

[272] Griffin K.J., Newell L.M., Simpson K.R., Beckers C.M.L., Drinkhill M.J., Standeven K.F., Cheah L.T., Iismaa S.E., Grant P.J., Jackson C.L. (2020). Transglutaminase 2 limits the extravasation and the resultant myocardial fibrosis associated with factor XIII-A deficiency. Atherosclerosis.

[273] Wu R., Li D., Zhang S., Wang J., Chen K., Tuo Z., Miyamoto A., Yoo K.H., Wei W., Zhang C. (2024). A pan-cancer analysis of the oncogenic and immunological roles of transglutaminase 1 (TGM1) in human cancer. J. Cancer Res. Clin. Oncol..

[274] Zhang W., Wu C., Zhou K., Cao Y., Zhou W., Zhang X., Deng D. (2022). Clinical and immunological characteristics of TGM3 in pan-cancer: A potential prognostic biomarker. Front. Genet..

[275] Coulton A., Murai J., Qian D., Thakkar K., Lewis C.E., Litchfield K. (2024). Using a pan-cancer atlas to investigate tumour associated macrophages as regulators of immunotherapy response. Nat. Commun..

[276] Sima L.E., Matei D., Condello S. (2022). The Outside-In Journey of Tissue Transglutaminase in Cancer. Cells.

[277] Li M., Wang X., Hong J., Mao J., Chen J., Chen X., Du Y., Song D. (2024). Transglutaminase 2 in breast cancer metastasis and drug resistance. Front. Cell Dev. Biol..

[278] Tempest R., Guarnerio S., Maani R., Cooper J., Peake N. (2021). The Biological and Biomechanical Role of Transglutaminase-2 in the Tumour Microenvironment. Cancers.

[279] Gao J., Wang S., Wan H., Lan J., Yan Y., Yin D., Zhou W., Hun S., He Q. (2023). Prognostic Value of Transglutaminase 2 in Patients with Solid Tumors: A Meta-analysis. Genet. Test. Mol. Biomark..

[280] Eckert R.L., Fisher M.L., Grun D., Adhikary G., Xu W., Kerr C. (2015). Transglutaminase is a tumor cell and cancer stem cell survival factor. Mol. Carcinog..

[281] Chen X., Adhikary G., Newland J.J., Xu W., Ma E., Naselsky W., Eckert R.L. (2023). The transglutaminase 2 cancer cell survival factor maintains mTOR activity to drive an aggressive cancer phenotype. Mol. Carcinog..

[282] Akbar A., McNeil N.M.R., Albert M.R., Ta V., Adhikary G., Bourgeois K., Eckert R.L., Keillor J.W. (2017). Structure-Activity Relationships of Potent, Targeted Covalent Inhibitors That Abolish Both the Transamidation and GTP Binding Activities of Human Tissue Transglutaminase. J. Med. Chem..

[283] Kerr C., Szmacinski H., Fisher M.L., Nance B., Lakowicz J.R., Akbar A., Keillor J.W., Lok Wong T., Godoy-Ruiz R., Toth E.A. (2017). Transamidase site-targeted agents alter the conformation of the transglutaminase cancer stem cell survival protein to reduce GTP binding activity and cancer stem cell survival. Oncogene.

[284] Kim S.Y. (2026). Targeting the Primordial Chaperone to Overcome Acquired Drug Resistance in Cancer: TG2-Mediated Autophagy. Biomol. Ther..

[285] Huang H., Chen Z., Ni X. (2017). Tissue transglutaminase-1 promotes stemness and chemoresistance in gastric cancer cells by regulating Wnt/beta-catenin signaling. Exp. Biol. Med..

[286] Hou J., Mei K., Wang D., Ke S., Chen X., Shang J., Li G., Gao Y., Xiong H., Zhang H. (2024). TGM1/3-mediated transamidation of Exo70 promotes tumor metastasis upon LKB1 inactivation. Cell Rep..

[287] Feng Y., Ji D., Huang Y., Ji B., Zhang Y., Li J., Peng W., Zhang C., Zhang D., Sun Y. (2020). TGM3 functions as a tumor suppressor by repressing epithelial-to-mesenchymal transition and the PI3K/AKT signaling pathway in colorectal cancer. Oncol. Rep..

[288] Zhou K., Wu C., Cheng W., Zhang B., Wei R., Cheng D., Li Y., Cao Y., Zhang W., Yao Z. (2024). Transglutaminase 3 regulates cutaneous squamous carcinoma differentiation and inhibits progression via PI3K-AKT signaling pathway-mediated Keratin 14 degradation. Cell Death Dis..

[289] Dubbink H.J., de Waal L., van Haperen R., Verkaik N.S., Trapman J., Romijn J.C. (1998). The human prostate-specific transglutaminase gene (TGM4): Genomic organization, tissue-specific expression, and promoter characterization. Genomics.

[290] Drabovich A.P., Saraon P., Drabovich M., Karakosta T.D., Dimitromanolakis A., Hyndman M.E., Jarvi K., Diamandis E.P. (2019). Multi-omics Biomarker Pipeline Reveals Elevated Levels of Protein-glutamine Gamma-glutamyltransferase 4 in Seminal Plasma of Prostate Cancer Patients. Mol. Cell Proteom..

[291] Yang C., Qu J., Wu J., Cai S., Liu W., Deng Y., Meng Y., Zheng L., Zhang L., Wang L. (2024). Single-cell dissection reveals immunosuppressive F13A1+ macrophage as a hallmark for multiple primary lung cancers. Clin. Transl. Med..

[292] Porrello A., Leslie P.L., Harrison E.B., Gorentla B.K., Kattula S., Ghosh S.K., Azam S.H., Holtzhausen A., Chao Y.L., Hayward M.C. (2018). Factor XIIIA-expressing inflammatory monocytes promote lung squamous cancer through fibrin cross-linking. Nat. Commun..

[293] Palumbo J.S., Barney K.A., Blevins E.A., Shaw M.A., Mishra A., Flick M.J., Kombrinck K.W., Talmage K.E., Souri M., Ichinose A. (2008). Factor XIII transglutaminase supports hematogenous tumor cell metastasis through a mechanism dependent on natural killer cell function. J. Thromb. Haemost..

[294] Shi Q., Ruan J., Yang Y.C., Shi X.Q., Liu S.D., Wang H.Y., Zhang S.J., Wang S.Q., Zhong L., Sun C. (2023). rs66651343 and rs12909095 confer lung cancer risk by regulating CCNDBP1 expression. PLoS ONE.

[295] Yongjun Zhang M.M., Zhang A., Hua Shi M.M., Xiangming Kong M.M. (2013). Association between TGM5, PPAP2B and PSMA4 polymorphisms and NSCLC in never-smoking Chinese population. J. Cancer Res. Ther..

[296] Zhou Y., Zang Y., Yang Y., Xiang J., Chen Z. (2019). Candidate genes involved in metastasis of colon cancer identified by integrated analysis. Cancer Med..

[297] Ha H.J., Kwon S., Jeong E.M., Kim C.M., Lee K.B., Kim I.G., Park H.H. (2018). Structure of natural variant transglutaminase 2 reveals molecular basis of gaining stability and higher activity. PLoS ONE.

[298] Forss A., Sotoodeh A., van Vollenhoven R.F., Ludvigsson J.F. (2024). Prevalence of coeliac disease in patients with rheumatoid arthritis and juvenile idiopathic arthritis: A systematic review and meta-analysis. Clin. Exp. Rheumatol..

[299] Sotoodeh A., Nguyen Hoang M., Hellgren K., Forss A. (2024). Prevalence of coeliac disease in patients with systemic lupus erythematosus: A systematic review and meta-analysis. Lupus Sci. Med..

[300] Marai I., Shoenfeld Y., Bizzaro N., Villalta D., Doria A., Tonutti E., Tozzoli R. (2004). IgA and IgG tissue transglutaminase antibodies in systemic lupus erythematosus. Lupus.

[301] Haggard L., Glimberg I., Lebwohl B., Sharma R., Verna E.C., Green P.H.R., Ludvigsson J.F. (2021). High prevalence of celiac disease in autoimmune hepatitis: Systematic review and meta-analysis. Liver Int..

[302] van Gerven N.M., Bakker S.F., de Boer Y.S., Witte B.I., Bontkes H., van Nieuwkerk C.M., Mulder C.J., Bouma G., Dutch A.I.H.w.g. (2014). Seroprevalence of celiac disease in patients with autoimmune hepatitis. Eur. J. Gastroenterol. Hepatol..

