# Supplementary material for: Human Transglutaminases: Updated Insights into Activation Mechanisms, Allosteric Regulation and Disease
Source: Int J Mol Sci. 2026 Mar 25;27(7):2976. doi: 10.3390/ijms27072976 (PMC13073648; doi:10.3390/ijms27072976)
Supplement: Supplementary file 1 [file ijms-27-02976-s001.zip › ijms-4175653-supplementary.pdf]

Table S1: Summary of available structural data for human transglutaminases (TG1, TG2, TG3 and FXIII)

Protein (Gene) /UNIPROT	PDB ID	Year	Experimental method / Expression system	Resolution (Å)	State/Ligands	Residue Range (% coverage)	Identity (%)	Missing residues	Ref
**TG1** (*TGM1*) / P22735	2XZZ	2011	X-ray / *E. coli*	2.30	Beta-barrel domain	693-787 (11.6%)	100%	-	To be published
**TG2** (*TGM2*) / P21980	8TR9	2024	cryo-EM / *E. coli*	3.20	Closed state /GDP	1-687 (100%)	100%	1-3	[1]
	9BC2	2024	X-ray / *E. coli*	2.75	Open state / inhibitor HB-225	1-687 (100%)	100%	307-308, 319-327, 362-366, 407-413, 462-471, 684-687	[2]
	9BC3	2024	X-ray / *E. coli*	2.52	Alternate state / Ca^{2+} & inhibitor HB-225	1-687 (100%)	100%	Chain A: 81-86, 318-327, 448-687<br>Chain B: 81-86, 318-327, 448-687	[2]
	9BC4	2024	X-ray / *E. coli*	1.84	Intermediate state / Ca^{2+} & inhibitor HB-225	1-687 (100%)	100%	1, 81-86, 320-327, 448-687	[2]
	6KZB	2020	X-ray / *E. coli*	3.55	Intermediate state/ Ca^{2+} & GDP	1-687 (100%)	100%	Chain A: 1, 84-85, 465, 469-470<br>Chain B: 1-2, 275, 327-328, 367-368, 466-469<br>Chain C: 1-8, 46-47, 52-56, 85-86, 120-130, 327-328, 363-367, 468-470	[3]
Mutant G224V (natural variant)	6A8P	2018	X-ray / *E. coli*	2.54	Closed state / GTP	1-695 (C-term his tag) (100%)	100%	Chain A: 464-470, 689-695<br>Chain B: 1-7, 122-127, 363-368, 464-470, 693-695<br>Chain C: 464-470, 688-695	[4]
	4PYG	2015	X-ray / *E. coli*	2.80	Closed state / GTP	1-687 (100%)	100%	Chain A: 1-3, 689-695<br>Chain B: 1-3, 689-695	[5]

							Chain C: 1-3, 689-695	
3S3J	2012	X-ray / *E. coli*	2.25	Open state / Z-DON (modified with 6-diazonio-5-oxo-L-norleucine)	2-687 (99.9%)	100%	306-309, 319-327, 362-366, 407-413, 462-470, 686-687	To be published
3S3P	2012	X-ray / *E. coli*	2.50	Open state / ZED754 (modified with (2S)-2-amino-7-ethoxy-7-oxoheptanoic acid)	2-687 (99.9%)	100%	240-252, 267-276, 305-308, 318-327, 362-369, 407-413, 462-471, 595-603, 684-687	To be published
3S3S	2012	X-ray / *E. coli*	2.30	Open state / ZED754 (modified with (2S)-2-amino-7-ethoxy-7-oxoheptanoic acid)	2-687 (99.9%)	100%	232, 239-252, 267-276, 306-308, 318-327, 362-369, 407-413, 462-471, 684-687	To be published
3LY6	2010	X-ray / *E. coli*	3.14	Closed state / ATP	1-687 (100%)	100%	Chain A: 1-3, 84-85, 689-697<br>Chain B: 1-3, 84-85, 689-697<br>Chain C: 1-3, 84-85, 689-697	[6]
2Q3Z	2007	X-ray / *E. coli*	2.00	Open state/ inhibitor (DP3-3)	1-687 (100%)	99.0% E51Q, E186Q, V224G, N533T, L655V	307-308, 319-327, 407-413, 462-471, 684-687	[7]

	1KV3	2002	X-ray / *E. coli*	2.80	Closed state / GDP	1-687 (100%)	99.0% E51Q, E186Q, V224G, N533T, L655V	Chain A: 1-14, 44-55, 123-132<br>Chain B: 1-14, 44-55, 123-132<br>Chain C: 1-14, 44-55, 123-132<br>Chain D: 1-14, 44-55, 123-132<br>Chain E: 1-14, 44-55, 123-132<br>Chain F: 1-14, 44-55, 123-132	[8]
**TG3** (*TGM3*) / Q08188	8RMX	2025	X-ray / *S. frugiperda*	2.80	Open state / Ca^{2+} & anti-TG3 Fab	1-464 (67.0%)	100%	Chain A: 1, 463-464<br>Chain D: 463-464	[9]
	8RMY	2025	X-ray / *S. frugiperda*	2.90	Open state / (Ca^{2+} & Z-DON & anti-TG3 Fab	1-464 (67.0%)	100%	Chain A: 1, 464<br>Chain D: 461-464	[9]
	8OXV	2023	X-ray / *S. frugiperda*	1.80	Closed state (zymogen) / Ca^{2+} & anti-TG3 Fab	1-693 (100%)	100%	462-473	[10]
	8OXW	2023	X-ray / *S. frugiperda*	1.70	Closed state (dispase-cleaved) / Ca^{2+}& anti-TG3 Fab	1-693 (100%)	100%	1, 461-473	[10]
	8OXX	2023	X-ray / *S. frugiperda*	2.50	Missing state (dispase-cleaved) / Ca^{2+} & (Z-DON & anti-TG3 Fab	2-461 (66.4%)	100%	-	[10]
	8OXY	2023	X-ray / *S. frugiperda*	2.00	Closed state (dispase-cleaved) / anti-TG3 Fab	1-693 (100%)	100%	1, 461-474	[10]

	1NUD	2003	X-ray / *S. frugiperda*	2.70	Open state (dispase-cleaved) / Ca^{2+}	2-693 (99.9%)	99.0% N251D, F265L, K562R, G654R	Chain A: 461-479<br>Chain B: 461-479	[11]
	1NUF	2003	X-ray / *S. frugiperda*	2.70	Closed state (dispase-cleaved) / Ca^{2+}	2-693 (99.9%)	99.0% N251D, F265L, K562R, G654R	462-478	[11]
	1NUG	2003	X-ray / *S. frugiperda*	2.40	Closed state (dispase-cleaved) / Ca^{2+} & Mg^{2+}	2-693 (99.9%)	99.0% N251D, F265L, K562R, G654R	Chain A: 462-478<br>Chain B: 460-472	[11]
	1L9M	2002	X-ray / *S. frugiperda*	2.10	Closed (zymogen) / Ca^{2+}	2-693 (99.9%)	99.0% N251D, F265L, K562R, G654R	Chain A: 462-478<br>Chain B: 461-471	[12]
	1L9N	2002	X-ray / *S. frugiperda*	2.10	Active state / Ca^{2+}	2-693 (99.9%)	99.0% 99.0% N251D, F265L, K562R, G654R	Chain A: 461-479<br>Chain B: 461-479	[12]
**FXIIIA** (*F13A1*)/ P00488 (FXIIIA);	8CMT	2024	cryo-EM / human plasma	3.04	A_2B_2 state (Inactive pro-enzyme heterotetrameric state)	Chain A: 1-732 (100%)	Chain A: 100%<br>Chain B: 100%	Chain A: 1-8, 731-732<br>Chain B: 1-8, 731-732<br>Chain C: 1-23, 331-661<br>Chain D: 1-23, 331-661	[13]

P05160 (FXIIIB)						Chain B: 1-661 (100%)		*(A and B are FXIIIA; C and D are FXIIB)*	
	8CMU	2024	cryo-EM / human plasma	2.41	A_2B_2 state (Inactive pro-enzyme heterotetrameric state)	Chain A: 1-732 (100%)<br><br>Chain B: 1-661 (100%)	Chain A: 100%<br><br>Chain B: 100%	Chain A: 1-8, 731-732<br>Chain B: 1-8, 731-732<br>Chain C: 1-23, 149-661<br>Chain D: 1-23, 149-661<br><br>*(A and B are FXIIIA; C and D are FXIIB)*	[13]
	5MHL	2017	X-ray / *S. frugiperda*	2.40	Active state / inhibitor Mi0621	Chain A: 2-732 (99.9%)	Chain A: 100%	Chain A: 1-14, 444-449, 502-515, 727-731<br>Chain B: 1-14, 445-449, 502-514, 643, 731	To be published
	5MH [illegible]	2017	X-ray / *S. frugiperda*	2.12	Active state / ZED1630	Chain A: 2-732 (99.9%)	Chain A: 100%	Chain A: 1–14, 354–359, 443–450, 502–515, 727–731<br>Chain B: 1–14, 355–358, 445–449, 502–515, 641–645, 726–731	To be published
	5MHN	2017	X-ray / *S. frugiperda*	2.48	Active state/ ZED2360	Chain A: 2-732 (99.9%)	Chain A: 100%	Chain A: 1–14, 355–359, 447–450, 502–515, 726–731<br>Chain B: 1–14, 355–359, 444–450, 502–515, 726–731	To be published
	5MHO	2017	X-ray / *S. frugiperda*	2.92	Active state / ZED2369	Chain A: 2-732 (99.9%)	Chain A: 100%	Chain A:<br>1–14, 354–359, 443–449, 501–515, 698–702, 726–731<br>Chain B:<br>1–14, 354–359, 444–449, 502–515, 641–645, 699–702, 726–731	To be published

	4KTY	2013	X-ray / *S. frugiperda*	1.98	Active state / Ca^{2+} & ZED1301	Chain A: 2-732 (99.9%)	Chain A: 100%	Chain A: 1–14, 447–448, 502–515, 726–731<br>Chain B: 1–14, 445–449, 502–514, 731	[14]
Mutant (W279F)	1EX0	2003	X-ray / *S. cerevisiae*	2.00	Inactive state (zymogen)/ Ca^{2+}	Chain A: 2-732 (99.9%)	Chain A: 99.0% V36N, P37L, W280F, E510S, G511R, V512S, 5652Q	Chain A: 1–6, 31–41, 511–515, 728–731<br>Chain B: 1–4, 37–41, 512–516, 731	To be published
	1EVU	2000	X-ray / *S. cerevisiae*	2.01	Closed state/ Ca^{2+}	Chain A: 2-732 (99.9%)	Chain A: 100%	Chain A: 31-42, 509-515, 728-731<br>Chain B: 1-7, 36-41, 509-514, 728-731	To be published
	1GGU	1999	X-ray / *S. cerevisiae*	2.10	Closed state/ Ca^{2+} bound	Chain A: 2-732 (99.9%)	Chain A: 100%	Chain A: 1-7, 31-42, 509-515, 728-731<br>Chain B: 1-7, 36-41, 509-515, 728-731	[15]
	1QRK	1999	X-ray / *S. cerevisiae*	2.50	Closed state/ Sr^{2+}	Chain A: 2-732 (99.9%)	Chain A: 100%	Chain A: 1-8, 31-44, 512-516, 728-731<br>Chain B: 1-9, 36-40, 508-515, 728-731	[15]
	1GGY	1999	X-ray / *S. cerevisiae*	2.50	Closed state / Yb^{3+}	Chain A: 2-732 (99.9%)	Chain A: 100%	Chain A: 1-7, 32-42, 509-515, 728-731<br>Chain B: 1-7, 34-41, 509-515, 728-731	[15]
	1F13	1998	X-ray / *S. cerevisiae*	2.10	Closed state (zymogen)	Chain A: 2-732 (99.9%)	Chain A: 100%	Chain A: 1-4, 37-38, 729-731<br>Chain B: 1-5, 37-40, 729-731	[16]
	1FIE	1997	X-ray / *S. cerevisiae*	2.50	Closed state (thrombin-cleaved)	Chain A: 2-732 (99.9%)	Chain A: 100%	Chain A: 1-8, 30-43, 728-731<br>Chain B: 1-9, 37-39, 728-731	[17]

	1GGT	1995	X-ray / *S. cerevisiae*	2.65	Closed state (zymogen)	Chain A: 2-732 (99.9%)	Chain A: 100%	Chain A: 1-7, 31-42, 730-731<br>Chain B: 1-7, 31-42, 728-731	[18]

Note: All structural annotations (bound ligands, missing residues, and sequence differences relative to UniProt) were extracted from the corresponding PDB coordinate/mmCIF files and the primary literature cited for each structure; any remaining discrepancies are unintentional

Table S2: Autoimmune and comorbid disorders involving TGs (non-gluten-driven)

Disease	TG isoform	Immune role*	Evidence strength	Key clinical message	Ref.
Acquired factor XIII A-subunit autoimmunity (AiF13D)	FXIIIA	Autoantigen	High	Severe bleeding despite normal PT/aPTT. Diagnosis requires FXIII activity/antigen and inhibitor testing. Treat with FXIII replacement plus immunosuppression	[19,20]
APS-1 (male subfertility / autoimmune prostatitis)	TG4	Autoantigen	High	Anti-TG4 frequent in adult male APS-1. Associated with autoimmune prostatitis and subfertility. Research biomarker but not used in routine diagnostics	[21,22]
IgA nephropathy (IgAN)	TG2	Pathway involvement	Moderate	TG2 participates in mesangial injury. Renal anti-TG2 deposits have been described in CeD linked subsets, supporting CeD evaluation in that context	[23,24]
Type 1 diabetes (T1D)	TG2	Comorbidity marker	Moderate	Anti-TG2 indicates coexisting CeD. Useful for CeD case-finding, not for pancreatic autoimmunity	[25,26]
Autoimmune thyroid disease (AITD)	TG2	Comorbidity marker	Moderate	Increased CeD prevalence. Anti-TG2 positivity should prompt CeD evaluation	[27]
Primary Sjögren's syndrome (pSS)	TG2	Comorbidity marker	Moderate	CeD prevalence increased. Anti-TG2 reflects CeD overlap, not salivary-gland autoimmunity	[28]
Rheumatoid arthritis (RA)	TG2	Comorbidity marker	Low-Moderate	Biopsy-confirmed CeD prevalence in RA is comparable to the general population. Routine CeD screening is not recommended in the absence of clinical suspicion	[29]
Juvenile idiopathic arthritis (JIA)	TG2	Comorbidity marker	Moderate	Overall CeD prevalence is not increased. Targeted CeD screening may be considered in JIA patients with additional risk factors or suggestive features	[29]
Systemic lupus erythematosus (SLE)	TG2	Comorbidity marker	Low-Moderate	Anti-TG2 positivity is more frequent than biopsy-confirmed CeD. Routine screening is not supported without clinical suspicion	[30,31]
Autoimmune hepatitis (AIH)	TG2	Comorbidity marker	Low-Moderate	Anti-TG2 positivity may reflect coexisting celiac disease rather than liver-specific TG autoimmunity	[32,33]

Multiple sclerosis (MS; progressive forms)	TG6	Exploratory	Preliminary	Low-frequency anti-TG6 seropositivity reported in small cohorts. Interpret as exploratory and not for routine testing	[34,35]
Amyotrophic lateral sclerosis (ALS)	TG6	Exploratory	Preliminary/ not supported	Anti-TG6 higher in one study. Conflicting serology reports. Insufficient evidence for clinical use.	[36,37]

**** Immune role categories:*** Autoantigen (pathogenic antibodies to a TG isoform); Comorbidity marker (anti-TG reflects coexisting CeD or broad autoimmunity, not organ targeting); Pathway involvement (TG participates functionally in lesion biology); Exploratory (inconsistent/early signals).

Table S3. Mendelian TG disorders at a glance

Gene / ORPHA	Disorder (common name)	Inheritance	Variants Spectrum (ClinVar*)	Main mechanism	Hallmark clinical	Key Ref
TGM1 / 313	Autosomal recessive congenital ichthyosis (ARCI). Predominantly LI	AR	143 P<br>112 LP<br>52 P/LP<br>253 VUS	TG1 deficiency impairs protein and lipid crosslinking during cornified envelope assembly, resulting in epidermal barrier failure	Typically starts with a collodion baby at birth and lifelong scaling; the broader clinical spectrum includes congenital ichthyosiform erythroderma, thermosensitive bathing-suit ichthyosis, self-healing collodion variants, and acral-limited forms	[38-40]
TGM3 / 1410	Uncombable hair syndrome type 2 (UHS2)	AR	23 P<br>1 LP<br>128 VUS	LOF mutations in *TGM3* impair TG3-mediated crosslinking of structural hair shaft proteins, leading to defective hair shaft formation and mechanical fragility	Marked by dry, frizzy, "spun-glass" hair with triangular or reniform shafts and longitudinal grooves, often ameliorating over time	[41,42]
TGM5 / 263534	Acral peeling skin syndrome (APSS)	AR	17 P<br>12 LP<br>2 P/LP<br>145 VUS	TG5 deficiency compromises cornified-envelope crosslinking in the outer epidermis and corneodesmosome stability, causing reduced stratum corneum cohesion and superficial epidermal splitting	Characterized by painless, recurrent superficial peeling of the skin, predominantly on the hands and feet	[43]
TGM6 / 276193	Spinocerebellar ataxia type 35 (SCA35)	AD	25 P<br>7 LP<br>274 VUS	LOF variants in TG6 reduce enzyme activity and cause protein mislocalization, triggering unfolded protein response and endoplasmic reticulum stress pathways that increase Purkinje cell vulnerability	Manifestations include gait and limb incoordination, dysarthria, variable tremor, and cerebellar atrophy	[44,45]
F13A1 / 331	Congenital factor XIII A (FXIIIA) -	AR	62 P<br>24 LP<br>2 P/LP	Loss of FXIIIA impairs fibrin cross-linking, resulting in mechanically	Neonatal umbilical bleeding, soft-tissue or muscle hemorrhage, easy bruising, poor wound healing; in women, menorrhagia or	[46]

	subunit deficiency		158 VUS	weak clots that form normally but are prone to delayed re-bleeding	recurrent pregnancy loss; life-threatening intracranial bleeding is the most serious complication	

* The ClinVar database was retrieved in February 2026. No confirmed Mendelian disorders have been reported to date for *TGM2*, *TGM4*, or *TGM7*.

Abbreviations: (alphabetical): AD autosomal dominant, AR autosomal recessive, aSHCB acral self-healing collodion baby, BSI bathing-suit ichthyosis, CIE congenital ichthyosiform erythroderma, LI lamellar ichthyosis, LOF Lost-of-function, LP likely pathogenic, P pathogenic, P/LP pathogenic or likely pathogenic, SHCB/SICB self-healing or self-improving collodion baby, VUS variant of uncertain significance.

REFERENCES:

1. Aplin, C.; Zielinski, K.A.; Pabit, S.; Ogunribido, D.; Katt, W.P.; Pollack, L.; Cerione, R.A.; Milano, S.K. Distinct conformational states enable transglutaminase 2 to promote cancer cell survival versus cell death. *Commun Biol* **2024**, *7*, 982, doi:10.1038/s42003-024-06672-x.
2. Sewa, A.S.; Besser, H.A.; Mathews, II; Khosla, C. Structural and mechanistic analysis of Ca(2+)-dependent regulation of transglutaminase 2 activity using a Ca(2+)-bound intermediate state. *Proc Natl Acad Sci U S A* **2024**, *121*, e2407066121, doi:10.1073/pnas.2407066121.
3. Jeong, E.M.; Lee, K.B.; Kim, G.E.; Kim, C.M.; Lee, J.H.; Kim, H.J.; Shin, J.W.; Kwon, M.A.; Park, H.H.; Kim, I.G. Competitive Binding of Magnesium to Calcium Binding Sites Reciprocally Regulates Transamidase and GTP Hydrolysis Activity of Transglutaminase 2. *Int J Mol Sci* **2020**, *21*, doi:10.3390/ijms21030791.
4. Ha, H.J.; Kwon, S.; Jeong, E.M.; Kim, C.M.; Lee, K.B.; Kim, I.G.; Park, H.H. Structure of natural variant transglutaminase 2 reveals molecular basis of gaining stability and higher activity. *PLoS One* **2018**, *13*, e0204707, doi:10.1371/journal.pone.0204707.
5. Jang, T.H.; Lee, D.S.; Choi, K.; Jeong, E.M.; Kim, I.G.; Kim, Y.W.; Chun, J.N.; Jeon, J.H.; Park, H.H. Crystal structure of transglutaminase 2 with GTP complex and amino acid sequence evidence of evolution of GTP binding site. *PLoS One* **2014**, *9*, e107005, doi:10.1371/journal.pone.0107005.
6. Han, B.G.; Cho, J.W.; Cho, Y.D.; Jeong, K.C.; Kim, S.Y.; Lee, B.I. Crystal structure of human transglutaminase 2 in complex with adenosine triphosphate. *Int J Biol Macromol* **2010**, *47*, 190-195, doi:10.1016/j.ijbiomac.2010.04.023.
7. Pinkas, D.M.; Strop, P.; Brunger, A.T.; Khosla, C. Transglutaminase 2 undergoes a large conformational change upon activation. *PLoS Biol* **2007**, *5*, e327, doi:10.1371/journal.pbio.0050327.
8. Liu, S.; Cerione, R.A.; Clardy, J. Structural basis for the guanine nucleotide-binding activity of tissue transglutaminase and its regulation of transamidation activity. *Proc Natl Acad Sci U S A* **2002**, *99*, 2743-2747, doi:10.1073/pnas.042454899.
9. Iversen, R.; Heggelund, J.E.; Das, S.; Hoydahl, L.S.; Sollid, L.M. Enzyme-activating B-cell receptors boost antigen presentation to pathogenic T cells in gluten-sensitive autoimmunity. *Nat Commun* **2025**, *16*, 2387, doi:10.1038/s41467-025-57564-5.
10. Heggelund, J.E.; Das, S.; Stamnaes, J.; Iversen, R.; Sollid, L.M. Autoantibody binding and unique enzyme-substrate intermediate conformation of human transglutaminase 3. *Nat Commun* **2023**, *14*, 6216, doi:10.1038/s41467-023-42004-z.
11. Ahvazi, B.; Boeshans, K.M.; Idler, W.; Baxa, U.; Steinert, P.M. Roles of calcium ions in the activation and activity of the transglutaminase 3 enzyme. *J Biol Chem* **2003**, *278*, 23834-23841, doi:10.1074/jbc.M301162200.
12. Ahvazi, B.; Kim, H.C.; Kee, S.H.; Nemes, Z.; Steinert, P.M. Three-dimensional structure of the human transglutaminase 3 enzyme: binding of calcium ions changes structure for activation. *EMBO J* **2002**, *21*, 2055-2067, doi:10.1093/emboj/21.9.2055.

13. Singh, S.; Hagelueken, G.; Ugurlar, D.; Urs, S.U.R.; Sharma, A.; Mahapatra, M.; Drepper, F.; Imhof, D.; Huesgen, P.F.; Oldenburg, J.; et al. Cryo-EM structure of the human native plasma coagulation factor XIII complex. *Blood* **2024**, doi:10.1182/blood.2024025369.
14. Stieler, M.; Weber, J.; Hils, M.; Kolb, P.; Heine, A.; Buchold, C.; Pasternack, R.; Klebe, G. Structure of active coagulation factor XIII triggered by calcium binding: basis for the design of next-generation anticoagulants. *Angew Chem Int Ed Engl* **2013**, *52*, 11930-11934, doi:10.1002/anie.201305133.
15. Fox, B.A.; Yee, V.C.; Pedersen, L.C.; Le Trong, I.; Bishop, P.D.; Stenkamp, R.E.; Teller, D.C. Identification of the calcium binding site and a novel ytterbium site in blood coagulation factor XIII by x-ray crystallography. *J Biol Chem* **1999**, *274*, 4917-4923, doi:10.1074/jbc.274.8.4917.
16. Weiss, M.S.; Metzner, H.J.; Hilgenfeld, R. Two non-proline cis peptide bonds may be important for factor XIII function. *FEBS Lett* **1998**, *423*, 291-296, doi:10.1016/s0014-5793(98)00098-2.
17. Yee, V.C.; Pedersen, L.C.; Bishop, P.D.; Stenkamp, R.E.; Teller, D.C. Structural evidence that the activation peptide is not released upon thrombin cleavage of factor XIII. *Thromb Res* **1995**, *78*, 389-397, doi:10.1016/0049-3848(95)00072-y.
18. Yee, V.C.; Pedersen, L.C.; Le Trong, I.; Bishop, P.D.; Stenkamp, R.E.; Teller, D.C. Three-dimensional structure of a transglutaminase: human blood coagulation factor XIII. *Proc Natl Acad Sci U S A* **1994**, *91*, 7296-7300, doi:10.1073/pnas.91.15.7296.
19. Duranteau, O.; Tatar, G.; Demulder, A.; Tuna, T. Acquired factor XIII deficiency: A scoping review. *Eur J Anaesthesiol Intensive Care* **2023**, *2*, e0035, doi:10.1097/EA9.0000000000000035.
20. Souri, M.; Osaki, T.; Ichinose, A. Detection of factor XIII inhibitors in 33 patients with autoimmune factor XIII deficiency in Japan. *Int J Hematol* **2024**, *120*, 472-481, doi:10.1007/s12185-024-03807-y.
21. Lopez-Bujanda, Z.A.; Obradovic, A.; Nirschl, T.R.; Crowley, L.; Macedo, R.; Papachristodoulou, A.; O'Donnell, T.; Laserson, U.; Zarif, J.C.; Reshef, R.; et al. TGM4: an immunogenic prostate-restricted antigen. *J Immunother Cancer* **2021**, *9*, doi:10.1136/jitc-2020-001649.
22. Iismaa, S.E. The prostate-specific protein, transglutaminase 4 (TG4), is an autoantigen associated with male subfertility. *Ann Transl Med* **2016**, *4*, S35, doi:10.21037/atm.2016.10.02.
23. Dutta, R.; Rawat, R.; Das, P.; Singh, G.; Kumari, A.; Ahmad, M.; Chauhan, A.; Ahuja, V.; Agrawal, S.K.; Makharia, G.K. Identification of celiac disease associated IgA nephropathy by IgA anti-tissue transglutaminase2 antibody deposits in archived formalin-fixed tissues. *Saudi J Gastroenterol* **2023**, *29*, 59-65, doi:10.4103/sjg.sjg_326_22.
24. Nurmi, R.; Korponay-Szabo, I.; Laurila, K.; Huhtala, H.; Niemela, O.; Mustonen, J.; Makela, S.; Kaukinen, K.; Lindfors, K. Celiac Disease-Type Tissue Transglutaminase Autoantibody Deposits in Kidney Biopsies of Patients with IgA Nephropathy. *Nutrients* **2021**, *13*, doi:10.3390/nu13051594.
25. Elfstrom, P.; Sundstrom, J.; Ludvigsson, J.F. Systematic review with meta-analysis: associations between coeliac disease and type 1 diabetes. *Aliment Pharmacol Ther* **2014**, *40*, 1123-1132, doi:10.1111/apt.12973.
26. Karimzadhagh, S.; Abbaspour, E.; Shahriarinamin, M.; Shamsi, P.; Poursadrolah, S.; Khorasani, M.; Daghighi, M.; Malek, A.; Talesh, J.T.; Makharia, G.K.; et al. Meta-Analysis: Global Prevalence of Coeliac Disease in Type 1 Diabetes. *Aliment Pharmacol Ther* **2025**, *61*, 8-31, doi:10.1111/apt.18373.
27. Roy, A.; Laszkowska, M.; Sundstrom, J.; Lebwohl, B.; Green, P.H.; Kampe, O.; Ludvigsson, J.F. Prevalence of Celiac Disease in Patients with Autoimmune Thyroid Disease: A Meta-Analysis. *Thyroid* **2016**, *26*, 880-890, doi:10.1089/thy.2016.0108.
28. Bartoloni, E.; Bistoni, O.; Alunno, A.; Cavagna, L.; Nalotto, L.; Baldini, C.; Priori, R.; Fischetti, C.; Fredi, M.; Quartuccio, L.; et al. Celiac Disease Prevalence is Increased in Primary Sjogren's Syndrome and Diffuse Systemic Sclerosis: Lessons from a Large Multi-Center Study. *J Clin Med* **2019**, *8*, doi:10.3390/jcm8040540.
29. Forss, A.; Sotoodeh, A.; van Vollenhoven, R.F.; Ludvigsson, J.F. Prevalence of coeliac disease in patients with rheumatoid arthritis and juvenile idiopathic arthritis: a systematic review and meta-analysis. *Clin Exp Rheumatol* **2024**, *42*, 608-618, doi:10.55563/clinexprheumatol/b92b8a.

30. Sotoodeh, A.; Nguyen Hoang, M.; Hellgren, K.; Forss, A. Prevalence of coeliac disease in patients with systemic lupus erythematosus: a systematic review and meta-analysis. *Lupus Sci Med* **2024**, *11*, doi:10.1136/lupus-2023-001106.
31. Marai, I.; Shoenfeld, Y.; Bizzaro, N.; Villalta, D.; Doria, A.; Tonutti, E.; Tozzoli, R. IgA and IgG tissue transglutaminase antibodies in systemic lupus erythematosus. *Lupus* **2004**, *13*, 241-244, doi:10.1191/0961203304lu1004oa.
32. Haggard, L.; Glimberg, I.; Lebwohl, B.; Sharma, R.; Verna, E.C.; Green, P.H.R.; Ludvigsson, J.F. High prevalence of celiac disease in autoimmune hepatitis: Systematic review and meta-analysis. *Liver Int* **2021**, *41*, 2693-2702, doi:10.1111/liv.15000.
33. van Gerven, N.M.; Bakker, S.F.; de Boer, Y.S.; Witte, B.I.; Bontkes, H.; van Nieuwkerk, C.M.; Mulder, C.J.; Bouma, G.; Dutch, A.I.H.w.g. Seroprevalence of celiac disease in patients with autoimmune hepatitis. *Eur J Gastroenterol Hepatol* **2014**, *26*, 1104-1107, doi:10.1097/MEG.0000000000000172.
34. Cristofanilli, M.; Gratch, D.; Pagano, B.; McDermott, K.; Huang, J.; Jian, J.; Bates, D.; Sadiq, S.A. Transglutaminase-6 is an autoantigen in progressive multiple sclerosis and is upregulated in reactive astrocytes. *Mult Scler* **2017**, *23*, 1707-1715, doi:10.1177/1352458516684022.
35. Emami, M.H.; Najafi, M.R.; Allahdadian, S.; Mohammadzadeh, S.; Jamali, N.; Lalazarian, A.; Shaygan Nejad, V.; Maghool, F. Evaluation of the Prevalence of Anti-transglutaminase 2 and 6 Antibodies in Patients with Sero-Positive Multiple Sclerosis. *Middle East J Dig Dis* **2024**, *16*, 47-51, doi:10.34172/mejdd.2024.368.
36. Gadoth, A.; Nefussy, B.; Bleiberg, M.; Klein, T.; Artman, I.; Drory, V.E. Transglutaminase 6 Antibodies in the Serum of Patients With Amyotrophic Lateral Sclerosis. *JAMA Neurol* **2015**, *72*, 676-681, doi:10.1001/jamaneurol.2015.48.
37. Visser, A.E.; Pazoki, R.; Pulit, S.L.; van Rheenen, W.; Raaphorst, J.; van der Kooi, A.J.; Ricano-Ponce, I.; Wijmenga, C.; Otten, H.G.; Veldink, J.H.; et al. No association between gluten sensitivity and amyotrophic lateral sclerosis. *J Neurol* **2017**, *264*, 694-700, doi:10.1007/s00415-017-8400-8.
38. Sun, Q.; Burgren, N.M.; Cheraghlou, S.; Paller, A.S.; Larralde, M.; Bercovitch, L.; Levinsohn, J.; Ren, I.; Hu, R.H.; Zhou, J.; et al. The Genomic and Phenotypic Landscape of Ichthyosis: An Analysis of 1000 Kindreds. *JAMA Dermatol* **2022**, *158*, 16-25, doi:10.1001/jamadermatol.2021.4242.
39. Surbek, M.; Van de Steene, T.; Sachslehner, A.P.; Golabi, B.; Griss, J.; Eyckerman, S.; Gevaert, K.; Eckhart, L. Cornification of keratinocytes is associated with differential changes in the catalytic activity and the immunoreactivity of transglutaminase-1. *Sci Rep* **2023**, *13*, 21550, doi:10.1038/s41598-023-48856-1.
40. Freedman, J.C.; Parry, T.J.; Zhang, P.; Majumdar, A.; Krishnan, S.; Regula, L.K.; O'Malley, M.; Coghlan, S.; Yogesha, S.D.; Ramasamy, S.; et al. Preclinical Evaluation of a Modified Herpes Simplex Virus Type 1 Vector Encoding Human TGM1 for the Treatment of Autosomal Recessive Congenital Ichthyosis. *J Invest Dermatol* **2021**, *141*, 874-882 e876, doi:10.1016/j.jid.2020.07.035.
41. Chermnykh, E.S.; Alpeeva, E.V.; Vorotelyak, E.A. Transglutaminase 3: The Involvement in Epithelial Differentiation and Cancer. *Cells* **2020**, *9*, doi:10.3390/cells9091996.
42. FB, U.B.; Cau, L.; Tafazzoli, A.; Mechin, M.C.; Wolf, S.; Romano, M.T.; Valentin, F.; Wiegmann, H.; Huchenq, A.; Kandil, R.; et al. Mutations in Three Genes Encoding Proteins Involved in Hair Shaft Formation Cause Uncombable Hair Syndrome. *Am J Hum Genet* **2016**, *99*, 1292-1304, doi:10.1016/j.ajhg.2016.10.004.
43. van der Velden, J.J.; van Geel, M.; Nellen, R.G.; Jonkman, M.F.; McGrath, J.A.; Nanda, A.; Sprecher, E.; van Steensel, M.A.; McLean, W.H.; Cassidy, A.J. Novel TGM5 mutations in acral peeling skin syndrome. *Exp Dermatol* **2015**, *24*, 285-289, doi:10.1111/exd.12650.
44. Guan, W.J.; Wang, J.L.; Liu, Y.T.; Ma, Y.T.; Zhou, Y.; Jiang, H.; Shen, L.; Guo, J.F.; Xia, K.; Li, J.D.; et al. Spinocerebellar ataxia type 35 (SCA35)-associated transglutaminase 6 mutants sensitize cells to apoptosis. *Biochem Biophys Res Commun* **2013**, *430*, 780-786, doi:10.1016/j.bbrc.2012.11.069.
45. Tripathy, D.; Vignoli, B.; Ramesh, N.; Polanco, M.J.; Coutelier, M.; Stephen, C.D.; Canossa, M.; Monin, M.L.; Aeschlimann, P.; Turberville, S.; et al. Mutations in TGM6 induce the unfolded protein response in SCA35. *Hum Mol Genet* **2017**, *26*, 3749-3762, doi:10.1093/hmg/ddx259.

46. Dorgalaleh, A.; Jozdani, S.; Zadeh, M.K. Factor XIII Deficiency: Laboratory, Molecular, and Clinical Aspects. *Semin Thromb Hemost* **2025**, *51*, 155-169, doi:10.1055/s-0044-1796673.
